# Upside and downside correlated jump risk premia of currency options and expected returns

**DOI:** 10.1186/s40854-023-00493-3

**Published:** 2023-05-03

**Authors:** Jie-Cao He, Hsing-Hua Chang, Ting-Fu Chen, Shih-Kuei Lin

**Affiliations:** 1 Department of Money and Banking, National Chengchi University, Taipei, Taiwan; 2 Department of Mathematics, National Central University, Taoyuan, Taiwan

**Keywords:** Jump-diffusion process, Currency option, Risk premia, Correlated jumps

## Abstract

This research explores upside and downside jumps in the dynamic processes of three rates: domestic interest rates, foreign interest rates, and exchange rates. To fill the gap between the asymmetric jump in the currency market and the current models, a correlated asymmetric jump model is proposed to capture the co-movement of the correlated jump risks for the three rates and identify the correlated jump risk premia. The likelihood ratio test results show that the new model performs best in 1-, 3-, 6-, and 12-month maturities. The in- and out-of-sample test results indicate that the new model can capture more risk factors with relatively small pricing errors. Finally, the risk factors captured by the new model can explain the exchange rate fluctuations for various economic events.

## Introduction

The extant literature has thoroughly examined the risk premia in financial markets. For example, regarding the variance risk premium of currency options, Bates (1996a) reported that it is positive for the currency options of United States dollars/German marks (USD/DEM) and USD/Japanese yen (JPY), while Low and Zhang (2005) reported that it is negative for euros/Swiss francs (EUR/CHF), JPY/Great British pounds (GBP), and JPY/EUR. Jurek and Xu (2014) calibrated a non-Gaussian model of pricing kernel dynamics employing G10 currency options as the basis for the estimation of conditional currency risk premia. Their results reveal that the skewness and higher-order moments of the pricing kernel merely account for an average of 15% of the conditional currency risk premia; however, jumps play an essential role.

**Fig. 1:**
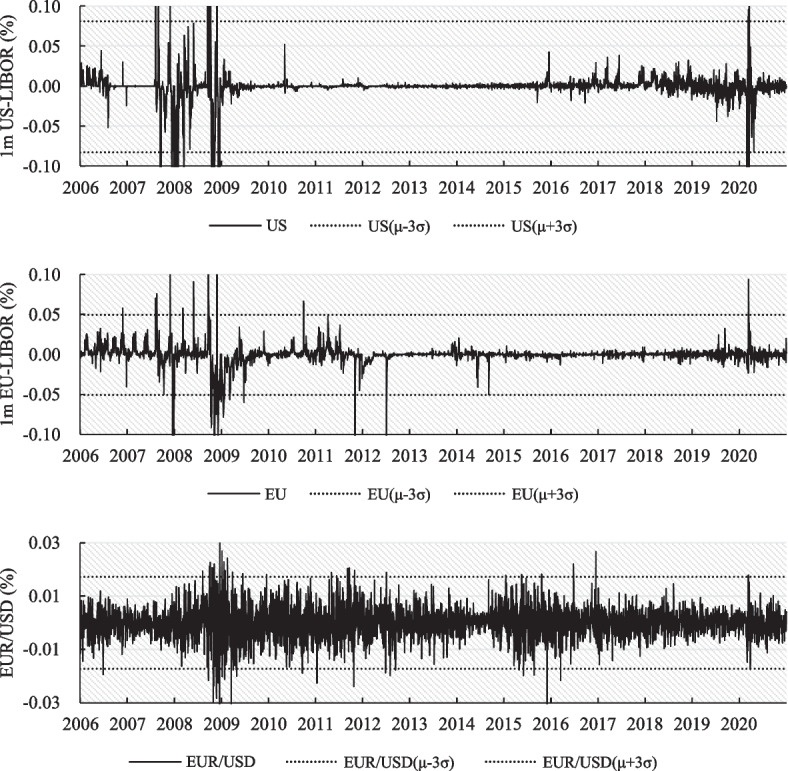
Time series of LIBORs and exchange rate. These data are the first-order differences for daily 1-month LIBOR and the log-return of the daily spot exchange rate with a sample period from January 2, 2006, to December 31, 2020

Figure 1 presents the returns of the 1-month United States (U.S.) and European Union (EU) London Interbank Offered Rates (LIBORs) and the log-return of the spot EUR/USD exchange rates. The figure indicates horizontal lines with plus or minus three standard deviations of these three assets’ returns. The returns of the 1-month U.S. and EU LIBORs and the log-return of EUR/USD were outside three standard deviations in 2008, 2009, and 2020. Fluctuations in exchange rates can occur for many reasons; however, interest rate parity (IRP) indicates a strong connection between interest rates and exchange rates. Changes in interest rates (U.S. and EU LIBORs) are likely to be the main driver of the exchange rate (EUR/USD) fluctuations.

Chinn (2006) provided evidence that the interest differential between the two countries predicts changes in the exchange rate of G7 countries and 14 emerging market countries. Du et al. (2018) also yielded similar empirical results from an analysis of the interest rates of the LIBOR, overnight indexed swap (OIS) rate, and the repo rate from the G10 currencies; the asymmetric volatility between the upside and downside can also be observed among these three assets, as illustrated in Fig. 1. For example, the figure shows that during the 2008 to 2009 financial crisis, the U.S. LIBOR’s jump frequency and amplitude in the downside area are more significant than those in the upside area. Conversely, during the 2020 COVID-19 pandemic, the EU LIBOR exhibited the opposite pattern, with a jump frequency and amplitude in the downside area smaller than those in the upside area.

The asymmetry in the frequency and amplitude of jumps in the upside and downside areas is also evident in different exchange rates; however, the volatility clustering of the jumps occurs simultaneously, indicating a strong connection among the three assets. This phenomenon represents volatility clustering and indicates that the jumps occurring in the three returns are highly correlated and asymmetric. Investors require compensation for undertaking such high volatility risks, called the jump risk premium.

In the empirical data, jumps among the three rates are correlated and asymmetrical in the upside and downside. Figure 1 indicates that the daily increments or daily log-return jumps outside three standard deviations are driven by the subprime mortgage crisis in 2008 and 2009, the EU debt crisis in 2010, and the COVID-19 pandemic in 2020; however, the occurrences of jumps on the upside and downside appear to be asymmetrical. Furthermore, we extend the model of Chuang et al. (2020) by assuming that jump frequencies and amplitudes are correlated but asymmetrical in the upside and downside among the three rates (domestic interest rates, foreign interest rates, and exchange rates). Based on this assumption, the correlated asymmetrical jump risk premia can be investigated in currency options, and the predictability of the risk premia can be assessed.

This paper provides four core research contributions, first by filling the asymmetric jump model gap in the currency option pricing models. This research derives a closed-form currency option pricing model considering the asymmetric correlation between upside and downside jumps. The decomposition helps to capture the risk premia of the upside and downside jumps among the domestic and foreign interest rates and the exchange rate. Second, the empirical results of the LRT demonstrate that the upside and downside correlated asymmetric jump model yields larger likelihood scores than the other four reduced models in all three currency pairs and all four maturities. Third, the in-sample and out-of-sample empirical results indicate that this paper’s proposed model still obtains minor pricing errors while capturing more risk factors. Finally, this research provides a broader scope for the economic interpretations of factors that drive currency risk premia. The risk factors captured by the asymmetric jump model can explain the exchange rate fluctuations in the occurrence of events, such as the subprime mortgage crisis.

This study is structured as follows. Section Literature review is a literature review, and Sect. The model presents the model’s assumptions and martingale conditions. Section Model assumption derives the pricing formula of the currency call options and introduces the parametric calibration method. Section Martingale conditions compares the empirical results with four reduced models and analyzes the upside and downside jump risk premia in different periods, and Sect. Pricing and calibration concludes.

## Literature review

Several studies have examined the role of jump risk premia in pricing financial instruments, such as the Standard & Poor’s 500 (S&P 500) index and options. For example, Pan (2002) constructed an arbitrage-free model to capture the stochastic volatility and jumps inherent in the S&P 500 index and near-the-money short-dated option prices. They presented substantiating evidence for a jump risk premium that strongly correlated with the volatility witnessed in the market. Chan and Feng (2012) tested the jumps in stock index futures (including the DAX, FTSE 100, Nikkei 225, and S&P 500 indices) with a series of GARCH models, finding significant associations between the jump risk premia and news events. Ornthanalai (2014) used a Lévy jump model to demonstrate the considerable role of the risk premium implied by infinite-activity jumps in S&P 500 index options. Additionally, Byun et al. (2015) developed a discrete-time option pricing model to examine the variance premium and jump risk premium in S&P 500 index option prices and returns over several decades. Li and Zinna (2018) proposed a pricing model to capture the co-jumps in prices, volatility, and self-exciting jump clustering. They examined the model with S&P 500 index returns and variance swap rates. These aforementioned studies examined the role of jump risk premia in the pricing of financial instruments and provided valuable insights into the dynamics of financial markets. Furthermore, Lin et al. (2014) developed a generalized pricing formula for European gold options via the Esscher transform, which considers the Markov-modulated jump-diffusion process. The study identified varying jump behavior of gold prices across different periods.

The jump risk premium for foreign exchange (FX) markets has also been well explored. Because the jump risk premium is not directly observable, it is extracted from the asset prices using the model-free or parametric approach, which is based on the volatility implied by options under the diffusion assumption in the model proposed by Britten‐Jones and Neuberger (2000). The option implied volatility forecasts the risk-neutral variance in different comparable maturity horizons (Jiang and Tian 2005; Wu and Carr 2009). Furthermore, the parametric approach extracts the jump risk premium using the dynamic pricing process of asset returns and derivatives (Chuang et al. 2020; Li and Zinna 2018). Because both methods have advantages and disadvantages, no consensus has been reached concerning which method is superior. The method selection depends on the researcher’s specific objectives. This study investigates the risk factors contributing to jumps in the upside and downside, adopting the parametric approach. Specifically, the parametric approach allows us to assess the impact of the jump components and to determine each impact factor’s explanatory and predictive power.

The parametric approach can be traced back to the classic closed-form European currency option pricing model proposed by Garman and Kohlhagen (1983), known as the GK model; however, Cookson (1992) provided evidence of the mispricing of currency options using the GK model. The main reasons are that currencies differ from stocks in some essential aspects and that the geometric Brownian motion cannot capture the behavior of currency returns (Ekvall et al. 1997). Many methodologies for currency option pricing have subsequently been proposed using modifications of the GK model, such as those of Chesney and Scott (1989), Amin and Jarrow (1991), Heston (1993), Bates (1996b), Sarwar and Krehbiel (2000), and Carr and Wu (2007).

Domestic and foreign interest rates play essential roles in pricing currency options, and Amin and Jarrow (1991) are pioneers in deriving a currency option pricing model that integrates domestic and foreign interest rate dynamic processes. The correlated Brownian pricing model (CB model) is based on the Heath–Jarrow–Morton (HJM) model (Heath et al. 1992) and constructs a general framework for pricing contingent claims on currency options.

With the development of stochastic models, Doffou and Hilliard (2001) extend the model of Bates (1992, 1996b) o account for the impact of interest rate and exchange rate jumps on currency futures, forward contracts, and futures options’ pricing with stochastic interest rate and stochastic volatility processes. According to Merton’s (1976) theory, Bo et al. (2010), Shokrollahi and Kılıçman (2015), Kim et al. (2019), and Chuang et al. (2020) consider the problem of pricing currency options under jump-diffusion processes in different settings. To capture the jump risk premium, Chuang et al. (2020) proposed a correlated Brownian motion with a correlated jump-diffusion model based on Merton (1973) and Amin and Jarrow (1991). More precisely, based on the CB model with the symmetrical jump assumption, Chuang et al. (2020) added the jump processes to the dynamic processes of the three assets simultaneously. Regarding the symmetrical jump models in their research, we define the model with independent jump processes in the three assets as the CB–ISJ (Correlated Brownian–Independent Symmetric Jump) model and that with the correlated jump processes as the CB–CSJ (Correlated Brownian–Correlated Symmetric Jump) model. The empirical results for EUR/USD, GBP/USD, and JPY/USD indicate a Poisson jump in all domestic interest rates, foreign interest rates, and exchange rates, all of which are correlated.

Numerous studies have established the prevalence of jumps in various financial assets (stock, stock index, futures, and options), leading scholars to investigate the asymmetry of upside and downside jumps. Frame and Ramezani (2014) used the affine jump-diffusion model to assess the asymmetric features of the S&P 500 index, the NASDAQ index, and selected stocks from 2007 to 2010, discovering that parameter estimates differ significantly under different economic conditions. Lau et al. (2019) utilized Gibbs sampling to estimate the asymmetric jump-diffusion process on five indexes (the DJIA, NASDAQ, FTSE 100, S&P 500, and NYSE ARCA Oil & Gas Index) from 2005 to 2014, indicating that the asymmetric jump-diffusion model is more accurate than the symmetric models in estimating fair prices of European call options. Alexeev et al. (2019) analyzed high-frequency data for the S&P 500 index from 2003 to 2017, finding that downside jumps significantly impact portfolio returns more than upside jumps, especially during extreme events. Ignoring these asymmetric jumps can increase exposure to severe negative market shifts.

Unlike stock derivatives, currency derivatives must consider dynamic processes for three assets: the exchange rate, domestic interest rate, and foreign interest rate. These three assets have their own parameters and interact with each other, significantly increasing the complexity of the estimation models. The current literature has yet to develop an asymmetric model for analyzing the exchange rate market, which commonly exhibits asymmetric jumps. Therefore, this research proposes the CB–CAJ (Correlated Brownian–Correlated Asymmetric Jump) model, which integrates correlated Brownian motions, correlated jump-diffusion processes, and asymmetric jumps in the upside and downside for the three assets simultaneously. We apply this model to capture such asymmetric jumps, analyze the risk premium of asymmetric jumps, and explain some economic phenomena.

## The model

According to the transformation discussed by Esscher (1932) and Gerber and Shiu (1994), the adjusted terms represent the risk premia of the variation for risk-neutral pricing. When applying the Esscher transform in a jump-diffusion process, the adjusted terms can capture the risk premia of both the continuous and jump parts; therefore, this research first defines the dynamic process for domestic and foreign interest rates and exchange rates with correlated Brownian motions and Poisson processes. This study subsequently examines the adjusted term of the continuous and jump risk with an Esscher transform derivation under the martingale condition of the correlated dynamic process.

### Model assumption

Based on the models proposed by Garman and Kohlhagen (1983), Amin and Jarrow (1991), and Chuang et al. (2020), this research proposes the dynamic processes of the correlated Brownian motions with a correlated asymmetric jump process (CB–CAJ) model. Under the ${\mathbb{P}}$-measure, the dynamic processes of a domestic forward interest rate (${f}_{D}$), foreign forward interest rate (${f}_{F}$), and exchange rate ($X$) are obtained by the following equations.

1 $$d{f}_{D}\left(t,T\right)={\alpha }_{D}\left(t,T\right)dt+{\sigma }_{D}\left(t,T\right)d{W}_{D}^{\mathbb{P}}\left(t\right)+{Y}_{D,u}\left(t,T\right)d{N}_{u}\left(t\right)+{Y}_{D,d}\left(t,T\right)d{N}_{d}\left(t\right),$$

2 $$d{f}_{F}\left(t,T\right)={\alpha }_{F}\left(t,T\right)dt+{\sigma }_{F}\left(t,T\right)d{W}_{F}^{\mathbb{P}}\left(t\right)+{Y}_{F,u}\left(t,T\right)d{N}_{u}\left(t\right)+{Y}_{F,d}\left(t,T\right)d{N}_{d}\left(t\right),$$

3 $$dX\left(t\right)=X\left(t-\right)\left\{{\mu }_{X}-{\lambda }_{u}\left[\mathrm{exp}\left({\theta }_{X,u}+\frac{{\nu }_{X,u}^{2}}{2}\right)-1\right]-{\lambda }_{d}\left[\mathrm{exp}\left({\theta }_{X,d}+\frac{{\nu }_{X,d}^{2}}{2}\right)-1\right]\right\}dt +X\left(t-\right){\sigma }_{X}d{W}_{X}^{\mathbb{P}}\left(t\right)+X\left(t-\right)\left[{Y}_{X,u}d{N}_{u}\left(t\right)+{Y}_{X,d}d{N}_{d}\left(t\right)\right],$$

where ${W}_{D}^{\mathbb{P}}\left(t\right)$, ${W}_{F}^{\mathbb{P}}\left(t\right)$, and ${W}_{X}^{\mathbb{P}}\left(t\right)$ are standard Brownian motions, and the correlations between them are ${\rho }_{DF}$, ${\rho }_{DX}$, and ${\rho }_{FX}$. ${Y}_{D,u}\left(t,T\right)d{N}_{u}\left(t\right)$, ${Y}_{F,u}\left(t,T\right)d{N}_{u}\left(t\right)$, and ${Y}_{X,u}d{N}_{u}\left(t\right)$ are the compound Poisson processes of upside jumps with the counting process ${N}_{u}\left(t\right)\sim Poisson\left({\lambda }_{u}t\right)$; the correlation coefficients of the correlated jump amplitudes are ${\phi }_{DF,u}$, ${\phi }_{DX,u}$, and ${\phi }_{FX,u}$. ${Y}_{D,d}\left(t,T\right)d{N}_{d}\left(t\right)$, ${Y}_{F,d}\left(t,T\right)d{N}_{d}\left(t\right)$, and ${Y}_{X,d}d{N}_{d}\left(t\right)$ are the compound Poisson processes of downside jumps with synchronous jumps ${N}_{d}\left(t\right)\sim Poisson\left({\lambda }_{d}t\right)$; the correlation coefficients of the correlated jump amplitudes are ${\phi }_{DF,d}$, ${\phi }_{DX,d}$, and ${\phi }_{FX,d}$.

The CB–CAJ model has four special conditions (reduced models). First, suppose the jump amplitudes of the upside and downside jumps are equal among the three assets, and the jump intensities of the upside and downside jumps are equal, that is, ${Y}_{D,u}\left(t,T\right)={Y}_{D,d}\left(t,T\right)$, ${Y}_{F,u}\left(t,T\right)={Y}_{F,d}\left(t,T\right)$, ${Y}_{X,u}={Y}_{X,d}$, and $d{N}_{u}\left(t\right)=d{N}_{d}\left(t\right)$. In this case, the model becomes a correlated Brownian model with correlated symmetric jump risks (CB–CSJ) (Chuang et al. 2020). Second, based on the first condition, if the jump amplitudes are independent, that is, ${\phi }_{DF}={\phi }_{FX}={\phi }_{XD}=0$, then the model will become a correlated Brownian model with independent symmetric jump risks (CB–ISJ). Third, if all three assets have no jump risk, that is, ${dN}_{u}\left(t\right)=d{N}_{d}\left(t\right)=0$, then the model will become a correlated Brownian model without a jump (CB) (Amin and Jarrow 1991). Finally, if all three assets have no jump risk and the two interest rates are constant, that is, ${dN}_{u}\left(t\right)=d{N}_{d}\left(t\right)=0$, ${f}_{D}\left(t,T\right)={r}_{D}$, and ${f}_{F}\left(t,T\right)={r}_{F}$, then the model will become that proposed by Garman and Kohlhagen (1983)—the GK model.

The impact of stochastic interest rate risk is pervasive and significant and has been the subject of considerable scholarly inquiry across various markets. Specifically, Kou et al. (2021) found that interest rates are crucial in predicting bankruptcy and default among small and medium-sized enterprises. As a result, this stochasticity can have significant implications for the valuation and pricing of financial instruments, including stocks, bonds, and derivatives.

For a given filtration ${\mathbb{F}}\left(t\right)$, according to Itô’s lemma, for a $T$-maturity zero-coupon bond (ZCB), the dynamic process is obtained by using the following equation.

4 $$\frac{d{P}_{k}\left(t,T\right)}{{P}_{k}\left(t,T\right)}=\left[{r}_{k}\left(t\right)-{\alpha }_{k}^{*}\left(t,T\right)+\frac{1}{2}{{\sigma }_{k}^{*}}^{2}\left(t,T\right)\right]dt-{\sigma }_{k}^{*}\left(t,T\right)d{W}_{k}^{\mathbb{P}}\left(t\right)+\left\{\mathrm{exp}\left[-{Y}_{k,u}^{*}\left(t,T\right)\right]-1\right\}d{N}_{u}\left(t\right)+\left\{\mathrm{exp}\left[-{Y}_{k,d}^{*}\left(t,T\right)\right]-1\right\}d{N}_{d}\left(t\right),$$

where $k\in \left\{D,F\right\}$ represents the domestic and foreign interest rates. Additionally, ${r}_{k}\left(t\right)={f}_{k} (t,t)$ is the instantaneous spot rate, ${\alpha }_{k}^{*}\left(t,T\right)={\int }_{t}^{T}{\alpha }_{k}\left(t,{t}_{\alpha }\right)d{t}_{\alpha }$ is the integration of the drift term, ${\sigma }_{k}^{*}\left(t,T\right)={\int }_{t}^{T}{\sigma }_{k}\left(t,{t}_{\alpha }\right)d{t}_{\alpha }$ is the integration of the diffusion term, ${Y}_{k,u}^{*}\left(t,T\right)={\int }_{t}^{T}{Y}_{k,u}\left(t,{t}_{\alpha }\right)d{t}_{\alpha }$ is the integration of the upside jump amplitude, and ${Y}_{k,d}^{*}\left(t,T\right)={\int }_{t}^{T}{Y}_{k,d}\left(t,{t}_{\alpha }\right)d{t}_{\alpha }$ is the integration of the downside jump amplitude. The numbers of upside and downside jumps during the period $\left[t,T\right)$ are $d{N}_{u}\left(t\right)\sim Poisson\left[{\lambda }_{u}\left(T-t\right)\right]$ and $d{N}_{d}\left(t\right)\sim Poisson\left[{\lambda }_{d}\left(T-t\right)\right]$, respectively.

The jump amplitudes in the three assets’ dynamic processes are assumed to have different distributions. This research assumes that the jump amplitudes follow a normal distribution to construct a straightforward model, with ${Y}_{D,u}^{*}\left(t,T\right)\sim Normal\left({\theta }_{D,u},{\nu }_{D,u}^{2}\right)$, ${Y}_{D,b}^{*}\left(t,T\right)\sim Normal\left({\theta }_{D,b},{\nu }_{D,b}^{2}\right)$, ${Y}_{F,u}^{*}\left(t,T\right)\sim Normal\left({\theta }_{F,u},{\nu }_{F,u}^{2}\right)$, ${Y}_{F,d}^{*}\left(t,T\right)\sim Normal\left({\theta }_{F,d},{\nu }_{F,d}^{2}\right)$, ${Y}_{X,u}\sim Normal\left({\theta }_{X,u},{\nu }_{X,u}^{2}\right)$, and ${Y}_{X,d}\sim Normal\left({\theta }_{X,d},{\nu }_{X,d}^{2}\right)$. Appendix 1 presents detailed proof of the dynamic processes concerning domestic and foreign interest rates.

Let the relative price be the relationship among the foreign ZCB, exchange rate $X\left(t\right){P}_{F}\left(t,T\right)$, and the domestic ZCB ${P}_{D}\left(t,T\right)$. Under the ${\mathbb{P}}$-measure, the dynamic process of the relative price $\frac{X\left(t\right){P}_{F}\left(t,T\right)}{{P}_{D}\left(t,T\right)}$ can be obtained using the following equation.

5 $$d\frac{X\left(t\right){P}_{F}\left(t,T\right)}{{P}_{D}\left(t,T\right)}=\frac{X\left(t\right){P}_{F}\left(t,T\right)}{{P}_{D}\left(t,T\right)}\left\{U\left(t,T\right)dt+\xi \left(t,T\right)d{\widetilde{W}}^{\mathbb{P}}\left(t\right)+{J}_{u}d{N}_{u}\left(t\right)+{J}_{d}\left(t\right)d{N}_{d}\left(t\right)+{J}_{u,d}\left(t\right)d{N}_{u,d}\left(t\right)\right\},$$

where

$$U\left(t,T\right)={\mu }_{X}-{r}_{D}\left(t\right)+{r}_{F}\left(t\right)+{\alpha }_{D}^{*}\left(t,T\right)-{\alpha }_{F}^{*}\left(t,T\right)-\frac{1}{2}{\sigma }_{X}^{2}+\frac{1}{2}{\xi }^{2}\left(t,T\right)-{\lambda }_{u}\left[\mathrm{exp}\left({\theta }_{X,u}+\frac{{\nu }_{X,u}^{2}}{2}\right)-1\right]-{\lambda }_{d}\left[\mathrm{exp}\left({\theta }_{X,d}+\frac{{\nu }_{X,d}^{2}}{2}\right)-1\right],$$

$${\xi }^{2}\left(t,T\right)={\sigma }_{X}^{2}+{{\sigma }_{D}^{*}}^{2}\left(t,T\right)+{{\sigma }_{F}^{*}}^{2}\left(t,T\right)-2{\rho }_{XF}{\sigma }_{X}{\sigma }_{F}^{*}\left(t,T\right)+2{\rho }_{XD}{\sigma }_{X}{\sigma }_{D}^{*}\left(t,T\right)-2{\rho }_{DF}{\sigma }_{F}^{*}\left(t,T\right){\sigma }_{D}^{*}\left(t,T\right),$$

$$\xi \left(t,T\right)d{\widetilde{W}}^{\mathbb{P}}\left(t\right)={\sigma }_{X}d{W}_{X}^{\mathbb{P}}\left(t\right)-{\sigma }_{F}^{*}\left(t,T\right)d{W}_{F}^{\mathbb{P}}\left(t\right)+{\sigma }_{D}^{*}\left(t,T\right)d{W}_{D}^{\mathbb{P}}\left(t\right),$$

$${J}_{u}={Y}_{X,u}-{Y}_{F,u}^{*}+{Y}_{D,u}^{*}-{Y}_{X,u}{Y}_{F,u}^{*}+{Y}_{X,u}{Y}_{D,u}^{*}-{Y}_{F,u}^{*}{Y}_{D,u}^{*}+{{Y}_{D,u}^{*}}^{2},$$

$${J}_{d}={Y}_{X,d}-{Y}_{F,d}^{*}+{Y}_{D,d}^{*}-{Y}_{X,d}{Y}_{F,d}^{*}+{Y}_{X,d}{Y}_{D,d}^{*}-{Y}_{F,d}^{*}{Y}_{D,d}^{*}+{{Y}_{D,d}^{*}}^{2},$$

$${J}_{u,d}=-{Y}_{X,d}{Y}_{F,u}^{*}-{Y}_{X,u}{Y}_{F,d}^{*}+{Y}_{X,u}{Y}_{D,d}^{*}+{Y}_{X,d}{Y}_{D,u}^{*}-{Y}_{F,u}^{*}{Y}_{D,d}^{*}-{Y}_{F,d}^{*}{Y}_{D,u}^{*}+2{Y}_{D,u}^{*}{Y}_{D,d}^{*}.$$

Our dynamic model considers numerous partitions of the time interval to obtain the differential of $t$. The occurrence of the Poisson process is a rare event because the intensity $\lambda dt$ is small; therefore, the probability of a simultaneous upside jump and downside jump occurring is negligible. Consequently, this study disregards the quadratic Poisson, $d{N}_{u}\left(t\right)d{N}_{d}\left(t\right)$; hence, ${J}_{u,d}d{N}_{u,d}\left(t\right)=0$.^1^

Let ${Y}_{T,u}$ and ${Y}_{T,d}$ be the total upside jump size and downside jump size, respectively. The aggregate jump size is a mixed distribution comprising the jump sizes in the foreign exchange and interest rate models.

6 $${J}_{u}d{N}_{u}\left(t\right)=\left({Y}_{X,u}-{Y}_{F,u}^{*}+{Y}_{D,u}^{*}-{Y}_{X,u}{Y}_{F,u}^{*}+{Y}_{X,u}{Y}_{D,u}^{*}-{Y}_{F,u}^{*}{Y}_{D,u}^{*}+{{Y}_{D,u}^{*}}^{2}\right)d{N}_{u}\left(t\right) \approx {Y}_{T,u} d{N}_{u}\left(t\right),$$

7 $${J}_{d}d{N}_{d}\left(t\right)=\left({Y}_{X,d}-{Y}_{F,d}^{*}+{Y}_{D,d}^{*}-{Y}_{X,d}{Y}_{F,d}^{*}+{Y}_{X,d}{Y}_{D,d}^{*}-{Y}_{F,d}^{*}{Y}_{D,d}^{*}+{{Y}_{D,d}^{*}}^{2}\right)d{N}_{d}\left(t\right) \approx {Y}_{T,d} d{N}_{d}\left(t\right),$$

8 $$d\frac{X\left(t\right){P}_{F}\left(t,T\right)}{{P}_{D}\left(t,T\right)}=\frac{X\left(t\right){P}_{F}\left(t,T\right)}{{P}_{D}\left(t,T\right)}\left\{U\left(t,T\right)dt+\xi \left(t,T\right)d{\widetilde{W}}^{\mathbb{P}}\left(t\right)+{Y}_{T,u} d{N}_{u}\left(t\right)+{Y}_{T,d} d{N}_{d}\left(t\right)\right\},$$

9 $$\frac{X\left(t\right){P}_{F}\left(t,T\right)}{{P}_{D}\left(t,T\right)}=\frac{X\left(0\right){P}_{F}\left(0,T\right)}{{P}_{D}\left(0,T\right)}\cdot \mathrm{exp}\left\{{\int }_{0}^{t}\left[U\left({t}_{\alpha },T\right)-\frac{1}{2}{\xi }^{2}\left({t}_{\alpha },T\right)\right]d{t}_{\alpha }+{\int }_{0}^{t}\xi \left({t}_{\alpha },T\right)d{\widetilde{W}}^{\mathbb{P}}\left({t}_{\alpha }\right)\right\}\mathrm{exp}\left[{Y}_{T,u}d{N}_{u}\left(t\right)\right]\cdot \mathrm{exp}\left[{Y}_{T,d}d{N}_{d}\left(t\right)\right],$$

10 $$d\mathrm{ln}\frac{X\left(t\right){P}_{F}\left(t,T\right)}{{P}_{D}\left(t,T\right)}=\left[U\left(t,T\right)-\frac{1}{2}{\xi }^{2}\left(t,T\right)\right]dt+\xi \left(t,T\right)d{\widetilde{W}}^{\mathbb{P}}\left(t\right)+{Y}_{T,u} d{N}_{u}\left(t\right)+{Y}_{T,d} d{N}_{d}\left(t\right).$$

This paper sets a normal distribution as the target for approximating the aggregate distribution of the total jump sizes. In other words, this research assumes that ${Y}_{T,u}$ and ${Y}_{T,d}$ follow normal distributions, that is, ${Y}_{T,u}\sim Normal\left({\theta }_{T,u},{\nu }_{T,u}^{2}\right)$ and ${Y}_{T,d}\sim Normal\left({\theta }_{T,d},{\nu }_{T,d}^{2}\right)$. Appendix 2 presents detailed proof of the dynamic process concerning the relative price.

### Martingale conditions

Because the underlying asset dynamics driven by a jump-diffusion process create an incomplete market, the number of equivalent martingale measures is infinite. The Esscher transform, developed by Esscher (1932) and Gerber and Shiu (1994), is widely used in finance to determine an equivalent martingale measure for the domestic interest rate, foreign interest rate, and exchange rate process. In the present paper, the Radon-Nikodým derivative is applied separately in the continuous and jump parts. Through the use of the frameworks employed in Gerber and Shiu (1996) and Lian et al. (2021), he Esscher transform for the exchange rate transform—under the measure ${\mathbb{Q}}^{{h}_{X}}\sim {\mathbb{P}}$ on the filtration $\mathcal{F}\left(t\right)$—with respect to ${h}_{1,X}$, ${h}_{X,u}$, and ${h}_{X,d}$ is obtained by using the following equation.

11 $$\left.\frac{d{\mathbb{Q}}^{{h}_{X}}}{d{\mathbb{P}}}\right|\mathcal{F}\left(t\right)=\mathrm{exp}\left\{{h}_{1,X}\xi \left(t,T\right)d{\widetilde{W}}^{\mathbb{P}}\left(t\right)-\frac{1}{2}{h}_{1,X}^{2}{\xi }^{2}\left(t,T\right)dt\right\}\mathrm{exp}\left\{{h}_{X,u}\cdot {Y}_{T,u} d{N}_{u}\left(t\right)-{\lambda }_{u}dt\left[\mathrm{exp}\left({h}_{X,u}{\theta }_{T,u}+\frac{1}{2}{h}_{X,u}^{2}{\nu }_{T,u}^{2}\right)-1\right]\right\} \mathrm{exp}\left\{{h}_{X,d}\cdot {Y}_{T,d} d{N}_{d}\left(t\right)-{\lambda }_{d}dt\left[\mathrm{exp}\left({h}_{X,d}{\theta }_{T,d}+\frac{1}{2}{h}_{X,d}^{2}{\nu }_{T,d}^{2}\right)-1\right]\right\}$$

where ${h}_{1,X}$, ${h}_{X,u}$, and ${h}_{X,d}$ represent the Esscher transform parameters of the continuous process, upside jump, and downside jump, respectively. These parameters are associated with the risk premia between the two equivalent measures ${\mathbb{Q}}^{{h}_{D}}$ and ${\mathbb{P}}$. Appendix 2 presents the detailed derivation of Eq. (11).

When deriving the pricing formula for the exchange rate derivatives, the domestic and foreign interest rates are the essential discounted factors for the pricing kernel, which must obtain the interest rate dynamics under a risk-neutral measure. Applying the Esscher transform again for the ZCB dynamics in Eq. (4), allows us to obtain the Radon–Nikodým derivatives for the domestic and foreign interest rates using the following equations.

12 $$\left.\frac{d{\mathbb{Q}}^{{h}_{D}}}{d{\mathbb{P}}}\right|\mathcal{F}\left(t\right)=\mathrm{exp}\left\{{h}_{1,D}{\sigma }_{D}^{*}\left(t,T\right)d{W}_{D}^{\mathbb{P}}\left(t\right)-\frac{1}{2}{h}_{1,D}^{2}{{\sigma }_{D}^{*}}^{2}\left(t,T\right)dt+{h}_{D,u}\cdot {Y}_{D,u}^{*}d{N}_{u}\left(t\right)+{h}_{D,d}\cdot {Y}_{D,d}^{*}d{N}_{d}\left(t\right)-{\lambda }_{u}dt\left[\mathrm{exp}\left({h}_{D,u}{\theta }_{D,u}+\frac{1}{2}{h}_{D,u}^{2}{\nu }_{D,u}^{2}\right)-1\right]-{\lambda }_{d}dt\left[\mathrm{exp}\left({h}_{D,d}{\theta }_{D,d}+\frac{1}{2}{h}_{D,d}^{2}{\nu }_{D,d}^{2}\right)-1\right]\right\},$$

13 $$\left.\frac{d{\mathbb{Q}}^{{h}_{F}}}{d{\mathbb{P}}}\right|\mathcal{F}\left(t\right)=\mathrm{exp}\left\{{h}_{1,F}{\sigma }_{F}^{*}\left(t,T\right)d{W}_{F}^{\mathbb{P}}\left(t\right)-\frac{1}{2}{h}_{1,F}^{2}{{\sigma }_{F}^{*}}^{2}\left(t,T\right)dt+{h}_{F,u}\cdot {Y}_{F,u}^{*}d{N}_{u}\left(t\right)+{h}_{F,d}\cdot {Y}_{F,d}^{*}d{N}_{d}\left(t\right)-{\lambda }_{u}dt\left[\mathrm{exp}\left({h}_{F,u}{\theta }_{F,u}+\frac{1}{2}{h}_{F,u}^{2}{\nu }_{F,u}^{2}\right)-1\right]-{\lambda }_{d}dt\left[\mathrm{exp}\left({h}_{F,d}{\theta }_{F,d}+\frac{1}{2}{h}_{F,d}^{2}{\nu }_{F,d}^{2}\right)-1\right]\right\}.$$

Equation (12) represents the Esscher transform for the domestic interest rate—under the equivalent measure on the filtration $\mathcal{F}\left(t\right)$—regarding ${h}_{1,D}$, ${h}_{D,u}$, and ${h}_{D,\mathrm{d}}$, which are the risk premium parameters for continuous process, upside jumps, and downside jumps, respectively. Similarly, Eq. (13) represents the Esscher transform for the foreign interest rate under the equivalent measure ${\mathbb{Q}}^{{h}_{F}}\sim {\mathbb{P}}$ with the transform parameters ${h}_{1,F}$, ${h}_{F,u}$, and ${h}_{F,d}$.

By applying the Radon–Nikodým derivative in Eqs. (11)–(13), this research can further determine the dynamic processes of the three assets under the risk-neutral measure and specify how the transform parameters influence the distributions.

Under the ${\mathbb{Q}}^{{h}_{D}}$ measure, the distribution of random variables is obtained by using the following equations.

14 $$d{W}_{D}^{{\mathbb{Q}}^{{h}_{D}}}\left(t\right)=d{W}_{D}^{\mathbb{P}}\left(t\right)-{h}_{1,D}{\sigma }_{D}^{*}\left(t,T\right),$$

15 $$d{N}_{u}\left(t\right)\sim Poisson\left({\lambda }_{u}dt\mathrm{exp}\left({h}_{D,u}{\theta }_{D,u}+\frac{1}{2}{h}_{D,u}^{2}{\nu }_{D,u}^{2}\right)\right),$$

16 $$d{N}_{d}\left(t\right)\sim Poisson\left({\lambda }_{d}dt\mathrm{exp}\left({h}_{D,d}{\theta }_{D,d}+\frac{1}{2}{h}_{D,d}^{2}{\nu }_{D,d}^{2}\right)\right),$$

17 $${Y}_{D,u}^{*}\stackrel{IID}{\sim }Normal\left({\theta }_{D,u}+{h}_{D,u}{\nu }_{D,u}^{2},{\nu }_{D,u}^{2}\right),$$

18 $${Y}_{D,d}^{*}\stackrel{IID}{\sim }Normal\left({\theta }_{D,d}+{h}_{D,d}{\nu }_{D,d}^{2},{\nu }_{D,d}^{2}\right).$$

Under the ${\mathbb{Q}}^{{h}_{F}}$ measure, the distribution of random variables is obtained by using the following equations.

19 $$d{W}_{D}^{{\mathbb{Q}}^{{h}_{F}}}\left(t\right)=d{W}_{F}^{\mathbb{P}}\left(t\right)-{h}_{1,F}{\sigma }_{F}^{*}\left(t,T\right),$$

20 $$d{N}_{u}\left(t\right)\sim Poisson\left({\lambda }_{u}dt\mathrm{exp}\left({h}_{F,u}{\theta }_{F,u}+\frac{1}{2}{h}_{F,u}^{2}{\nu }_{F,u}^{2}\right)\right),$$

21 $$d{N}_{d}\left(t\right)\sim Poisson\left({\lambda }_{d}dt\mathrm{exp}\left({h}_{F,d}{\theta }_{F,d}+\frac{1}{2}{h}_{F,d}^{2}{\nu }_{F,d}^{2}\right)\right),$$

22 $${Y}_{F,u}^{*}\stackrel{IID}{\sim }Normal\left({\theta }_{F,u}+{h}_{F,u}{\nu }_{F,u}^{2},{\nu }_{F,u}^{2}\right),$$

23 $${Y}_{F,d}^{*}\stackrel{IID}{\sim }Normal\left({\theta }_{F,d}+{h}_{F,d}{\nu }_{F,d}^{2},{\nu }_{F,d}^{2}\right).$$

Under the ${\mathbb{Q}}^{{h}_{X}}$-measure, the distribution of random variables is obtained by using the following equations.

24 $$d{\widetilde{W}}^{{\mathbb{Q}}^{{h}_{X}}}\left(t\right)=d{\widetilde{W}}^{\mathbb{P}}\left(t\right)-{h}_{1,X}\xi \left(t,T\right),$$

25 $$d{N}_{u}\left(t\right)\sim Poisson\left({\lambda }_{u}dt\mathrm{exp}\left({h}_{X,u}{\theta }_{T,u}+\frac{1}{2}{h}_{X,u}^{2}{\nu }_{T,u}^{2}\right)\right),$$

26 $$d{N}_{d}\left(t\right)\sim Poisson\left({\lambda }_{d}dt\mathrm{exp}\left({h}_{X,d}{\theta }_{T,d}+\frac{1}{2}{h}_{X,d}^{2}{\nu }_{T,d}^{2}\right)\right),$$

27 $${Y}_{T,u}\stackrel{IID}{\sim }Normal\left({\theta }_{T,u}+{h}_{X,u}{\nu }_{T,u}^{2}, {\nu }_{T,u}^{2}\right),$$

28 $${Y}_{T,d}\stackrel{IID}{\sim }Normal\left({\theta }_{T,d}+{h}_{X,d}{\nu }_{T,d}^{2},{\nu }_{T,d}^{2}\right).$$

Appendices A and B present detailed proofs for the change of measure regarding the interest rates and relative price, respectively.

According to the asset pricing theory discussed by Harrison and Pliska (1981), Musiela and Rutkowski (1998), and Brigo and Mercurio (2006), without arbitrage opportunities in domestic and foreign bond markets, the relative price follows a martingale under the T-forward probability measure (${\mathbb{Q}}_{D}^{T}$). By selecting ${B}_{D}\left(t\right)=\mathrm{exp}\left[{\int }_{0}^{t}{r}_{D}\left(u\right)du\right]$ as the domestic money market account, the martingale conditions of the three assets are obtained by using the following equations:

29 $$\left[-{\alpha }_{D}^{*}\left(t,T\right)+\frac{1}{2}{{\sigma }_{D}^{*}}^{2}\left(t,T\right)-{h}_{1,D}{{\sigma }_{D}^{*}}^{2}\left(t,T\right)\right]-{\lambda }_{u}\left[\mathrm{exp}\left({h}_{D,u}{\theta }_{D,u}+\frac{1}{2}{h}_{D,u}^{2}{\nu }_{D,u}^{2}\right)-1\right]-{\lambda }_{d}\left[\mathrm{exp}\left({h}_{D,d}{\theta }_{D,d}+\frac{1}{2}{h}_{D,d}^{2}{\nu }_{D,d}^{2}\right)-1\right]=0,$$

30 $$\left\{\left[{r}_{F}\left(t\right)-{r}_{D}\left(t\right)-{\alpha }_{F}^{*}\left(u,T\right)\right]+\frac{1}{2}{{\sigma }_{F}^{*}}^{2}\left(t,T\right)-{h}_{1,F}{{\sigma }_{F}^{*}}^{2}\left(t,T\right)\right\}-{\lambda }_{u}\left[\mathrm{exp}\left({h}_{F,u}{\theta }_{F,u}+\frac{1}{2}{h}_{F,u}^{2}{\nu }_{F,u}^{2}\right)-1\right]-{\lambda }_{d}\left[\mathrm{exp}\left({h}_{F,d}{\theta }_{F,d}+\frac{1}{2}{h}_{F,d}^{2}{\nu }_{F,d}^{2}\right)-1\right]=0,$$

31 $$\left\{\left[U\left(t,T\right)-{r}_{D}\right]-\frac{1}{2}{h}_{1,X}^{2}{\xi }^{2}\left(t,T\right)\right\}-{\lambda }_{u}\left[\mathrm{exp}\left({h}_{X,u}{\theta }_{T,u}+\frac{1}{2}{h}_{X,u}^{2}{\nu }_{T,u}^{2}\right)-1\right]-{\lambda }_{d}\left[\mathrm{exp}\left({h}_{X,d}{\theta }_{T,d}+\frac{1}{2}{h}_{X,d}^{2}{\nu }_{T,d}^{2}\right)-1\right]=0.$$

The Esscher transform has nine parameters (i.e., ${h}_{1,D}$, ${h}_{D,u}$, ${h}_{D,d}$,${h}_{1,F}$, ${h}_{F,u}$, ${h}_{F,d}$,${h}_{1,X}$, ${h}_{X,u}$, and ${h}_{X,d}$), which represent the three assets’ continuous and jump risks. That is, if the domestic bond market, foreign bond market, or exchange rate is exposed to substantial risks, the parameters above are the risk premia that serve as compensation for associated continuous risks and jump risks.

## Pricing and calibration

Based on the assumptions and the martingale conditions in Sect. Literature review , the first part derives the pricing formula of the correlated Brownian motions using the asymmetric jump process model with a closed-form. The second part presents the parametric calibration method of the expectation–maximization (EM) algorithm associated with the maximum weighted joint log-likelihood.

### Option pricing formula

For a currency call option with a maturity $T$ and a strike price $K$, and for filtration ${\mathbb{F}}\left(t\right)$, the martingale method and risk-neutral measure are applied to derive the pricing formula with the CB–CSJ model using the following equation.

32 $$C\left(t\right)=X\left(t\right){P}_{F}\left(t,T\right){\sum }_{{n}_{u}=0}^{\infty }{\sum }_{{n}_{d}=0}^{\infty }\frac{{e}^{-{q}_{u}\left(t,T\right)}{\left[{q}_{u}\left(t,T\right)\right]}^{{n}_{u}}}{{n}_{u}!}\cdot \frac{{e}^{-{q}_{d}\left(t,T\right)}{\left[{q}_{d}\left(t,T\right)\right]}^{{n}_{d}}}{{n}_{d}!}{e}^{\Gamma \left({n}_{u},{n}_{d},t,T\right)} N\left({d}_{1}\right)-K{P}_{D}\left(t,T\right){\sum }_{{n}_{u}=0}^{\infty }{\sum }_{{n}_{d}=0}^{\infty }\frac{{e}^{-{q}_{u}\left(t,T\right)}{\left[{q}_{u}\left(t,T\right)\right]}^{{n}_{u}}}{{n}_{u}!}\cdot \frac{{e}^{-{q}_{d}\left(t,T\right)}{\left[{q}_{d}\left(t,T\right)\right]}^{{n}_{d}}}{{n}_{d}!}{e}^{\Gamma \left({n}_{u},{n}_{d},t,T\right)}N\left({d}_{2}\right),$$

where $N\left(\cdot \right)$ is the cumulative distribution function (CDF) of a standard normal distribution,

$${d}_{1}=\frac{\mathrm{ln}\frac{X\left(t\right){P}_{F}\left(t,T\right)}{K{P}_{D}\left(t,T\right)}+\Gamma \left({n}_{u},{n}_{d},t,T\right)+\frac{1}{2}\Phi \left({n}_{u},{n}_{d},t,T\right)}{\sqrt{\Phi \left({n}_{u},{n}_{d},t,T\right)}}{d}_{2}={d}_{1}-\sqrt{\Phi \left({n}_{u},{n}_{d},t,T\right)},$$

and

$${q}_{u}\left(t,T\right)={\lambda }_{u}\left(T-t\right)\mathit{exp}\left({h}_{X,u}{\theta }_{T,u}+\frac{1}{2}{h}_{X,u}^{2}{\nu }_{T,u}^{2}\right),{q}_{d}\left(t,T\right)={\lambda }_{d}\left(T-t\right)\mathit{exp}\left({h}_{X,d}{\theta }_{T,d}+\frac{1}{2}{h}_{X,d}^{2}{\nu }_{T,d}^{2}\right).$$

${q}_{u}\left(t,T\right)$ and ${q}_{d}\left(t,T\right)$ are the upside and downside jump intensity under ${\mathbb{Q}}_{D}^{T}$ measure, and $\Theta \left({n}_{u},{n}_{d},{h}_{X,\mathrm{u}}\right)$ and $\Phi \left({n}_{u},{n}_{d},t,T\right)$ are, respectively, the mean and variance of the relative price $\frac{X\left(t\right){P}_{F}\left(t,T\right)}{{P}_{D}\left(t,T\right)}$ under ${\mathbb{Q}}_{D}^{T}$ measure, defined in Eq. (107).Appendix 3 presents detailed proof of the pricing formula.

The four reduced models can also be derived from Eq. (32). First, suppose the jump amplitudes of upside jumps and downside jumps among the three assets are equal, and the jump intensities of an upside jump and a downside jump are equal (CB–CSJ model); that is, ${Y}_{D,u}\left(t,T\right)={Y}_{D,\mathrm{d}}\left(t,T\right)$, ${Y}_{F,u}\left(t,T\right)={Y}_{F,\mathrm{d}}\left(t,T\right)$, ${Y}_{X,\mathrm{u}}={Y}_{X,\mathrm{d}}$, and $d{N}_{u}\left(t\right)=d{N}_{d}\left(t\right)$.In this case, the pricing formula of a currency call option is as follows

33 $$C\left(t\right)=X\left(t\right){P}_{F}\left(t,T\right){\sum }_{n=0}^{\infty }\frac{{e}^{-q\left(t,T\right)}{\left[q\left(t,T\right)\right]}^{n}}{n!}{e}^{\Gamma \left(n,t,T\right)} N\left({d}_{1}\right)-K{P}_{D}\left(t,T\right){\sum }_{n=0}^{\infty }\frac{{e}^{-q\left(t,T\right)}{\left[q\left(t,T\right)\right]}^{n}}{n!}{e}^{\Gamma \left(n,t,T\right)}N\left({d}_{2}\right),$$

where

$${d}_{1}=\frac{\mathrm{ln}\frac{X\left(t\right){P}_{F}\left(t,T\right)}{K{P}_{D}\left(t,T\right)}+\Gamma \left(n,t,T\right)+\frac{1}{2}\Phi \left(n,t,T\right)}{\sqrt{\Phi \left(n,t,T\right)}},{d}_{2}={d}_{1}-\sqrt{\Phi \left(n,t,T\right)},q\left(t,T\right)=\lambda \left(T-t\right)\mathrm{exp}\left({h}_{X}{\theta }_{T}+\frac{1}{2}{h}_{X}^{2}{\nu }_{T}^{2}\right).$$

Second, suppose the jump amplitudes of upside and downside jumps among the three assets are equal, the jump intensities of an upside jump and a downside jump are equal, and all the jump amplitudes are independent, that is,${Y}_{D,u}\left(t,T\right)={Y}_{D,\mathrm{d}}\left(t,T\right)$, ${Y}_{F,\mathrm{u}}\left(t,T\right)={Y}_{F,\mathrm{d}}\left(t,T\right)$, ${Y}_{X,u}={Y}_{X,d}$, $d{N}_{u}\left(t\right)=d{N}_{d}\left(t\right)$, and ${\phi }_{DF}={\phi }_{FX}={\phi }_{XD}=0$. In this case, the model becomes a correlated Brownian model with independent symmetric jump risks (CB–ISJ). The pricing formula for a currency call option is as follows.

34 $$C\left(t\right)=X\left(t\right){P}_{F}\left(t,T\right){\sum }_{n=0}^{\infty }\frac{{e}^{-q\left(t,T\right)}{\left[q\left(t,T\right)\right]}^{n}}{n!}{e}^{\Gamma \left(n,t,T\right)} N\left({d}_{1}\right)-K{P}_{D}\left(t,T\right){\sum }_{n=0}^{\infty }\frac{{e}^{-q\left(t,T\right)}{\left[q\left(t,T\right)\right]}^{n}}{n!}{e}^{\Gamma \left(n,t,T\right)}N\left({d}_{2}\right),$$

where

$${d}_{1}=\frac{\mathrm{ln}\frac{X\left(t\right){P}_{F}\left(t,T\right)}{K{P}_{D}\left(t,T\right)}+\Gamma \left(n,t,T\right)+\frac{1}{2}\Phi \left(n,t,T\right)}{\sqrt{\Phi \left(n,t,T\right)}},{d}_{2}={d}_{1}-\sqrt{\Phi \left(n,t,T\right)},q\left(t,T\right)=\lambda \left(T-t\right)\mathrm{exp}\left({h}_{X}{\theta }_{T}+\frac{1}{2}{h}_{X}^{2}{\nu }_{T}^{2}\right).$$

Third, if all three assets have no jump risk, that is ${dN}_{u}\left(t\right)=d{N}_{d}\left(t\right)=0$, then the model becomes a correlated Brownian model without jumps. The pricing formula for the currency call option is as follows.

35 $$C\left(t\right)=X\left(t\right){P}_{F}\left(t,T\right)N\left({d}_{1}\right)-K{P}_{D}\left(t,T\right)N\left({d}_{2}\right),$$

where

$${d}_{1}=\frac{\mathrm{ln}\frac{X\left(t\right){P}_{F}\left(t,T\right)}{K{P}_{D}\left(t,T\right)}+\frac{1}{2}\Phi \left(t,T\right)}{\sqrt{\Phi \left(t,T\right)}},{d}_{2}={d}_{1}-\sqrt{\Phi \left(t,T\right)}.$$

Finally, if all three assets have no jump risk, and the two interest rates are constant, that is, ${dN}_{u}\left(t\right)=d{N}_{d}\left(t\right)=0$, ${f}_{D}\left(t,T\right)={r}_{D}$, and ${f}_{F}\left(t,T\right)={r}_{F}$, then the model becomes the GK model proposed by Garman and Kohlhagen (1983). The pricing formula of a currency call option is as follows.

36 $$C\left(t\right)=X\left(t\right){P}_{F}\left(t,T\right) N\left({d}_{1}\right)-K{P}_{D}\left(t,T\right)N\left({d}_{2}\right),$$

where

$${d}_{1}=\frac{\mathrm{ln}\frac{X\left(t\right)}{K}+\frac{1}{2}\Phi \left(t,T\right)}{\sqrt{\Phi \left(t,T\right)}},{d}_{2}={d}_{1}-\sqrt{\Phi \left(t,T\right)}.$$

### Parametric calibration

#### Joint estimation with returns and option prices

Parametric estimation is a major component of the this current research. Extant studies that examine the risk premium in currency options have mainly used single measures (only return data) to calibrate poorly-identified risk premium parameters.

Following Christoffersen et al. (2012) and Ornthanalai (2014), we calibrate the parameters by maximizing the weighted joint log-likelihood function through returns and option prices.

37 $$L\left(\Theta \right)=\frac{{T}_{Ret}+{T}_{Opt}}{2}\left(\frac{{L}_{Ret}\left(\Theta \right)}{{T}_{Ret}}+\frac{{L}_{Opt}\left(\Theta \right)}{{T}_{Opt}}\right),$$

where $\Theta$ is the parameter set of the model. $L\left(\Theta \right)$, ${L}_{Ret}\left(\Theta \right)$, and ${L}_{Opt}\left(\Theta \right)$ are the log-likelihood of joint estimation, returns, and option prices, respectively. ${T}_{Ret}$ and ${T}_{Opt}$ are the total number of observed returns and option prices, respectively.

$\widetilde{R}=\left\{\widetilde{R}\left(1\right),\cdots ,\widetilde{R}\left(T\right)\right\}$ is denoted as the daily return, and $\widetilde{N}=\left\{N\left(1\right),\cdots ,N\left(T\right)\right\}$ is denoted as the jump intensity of daily return; therefore, the likelihood function is obtained by using the following equation.

38 $${f}^{\mathbb{P}}\left[\left.\widetilde{R}\left(t\right)\right|{N}_{u}\left(t\right)={n}_{u},{N}_{d}\left(t\right)={n}_{d}\right]=\frac{\mathrm{exp}\left\{-\frac{1}{2}{\left[\widetilde{R}\left(t\right)-\left(\widetilde{\mu }+{n}_{u}\widetilde{{\theta }_{u}}+{n}_{d}\widetilde{{\theta }_{d}}\right)\right]}^{\mathrm{^{\prime}}}{\left({\sum }_{1}+{n}_{u}^{2}{\sum }_{d}+{n}_{u}^{2}{\sum }_{d}\right)}^{-1}\left[\widetilde{R}\left(t\right)-\left(\widetilde{\mu }+{n}_{u}\widetilde{{\theta }_{d}}+{n}_{u}\widetilde{{\theta }_{d}}\right)\right]\right\}}{\sqrt{{\left(2\pi \right)}^{3}\left|{\sum }_{1}+{n}_{u}^{2}{\sum }_{u}+{n}_{d}^{2}{\sum }_{d}\right|}},$$

where

$$\widetilde{R}\left(t\right)=\left[\begin{array}{c}{R}_{D}\left(t\right)\\ {R}_{F}\left(t\right)\\ {R}_{X}\left(t\right)\end{array}\right], \widetilde{\mu }=\left[\begin{array}{c}{\mu }_{D}\\ {\mu }_{F}\\ {\mu }_{X}\end{array}\right], \widetilde{\theta }=\left[\begin{array}{c}-{\theta }_{D}\left(T-t\right)\\ -{\theta }_{F}\left(T-t\right)\\ {\theta }_{X}\end{array}\right],{\sum }_{1}=\left[\begin{array}{ccc}{\sigma }_{D}^{2}& {\sigma }_{D}{\sigma }_{F}{\rho }_{DF}& {\sigma }_{D}{\sigma }_{X}{\rho }_{DX}\\ {\sigma }_{D}{\sigma }_{F}{\rho }_{DF}& {\sigma }_{F}^{2}& {\sigma }_{D}{\sigma }_{X}{\rho }_{DX}\\ {\sigma }_{D}{\sigma }_{X}{\rho }_{DX}& {\sigma }_{D}{\sigma }_{X}{\rho }_{DX}& {\sigma }_{X}^{2}\end{array}\right]{\sum }_{g}=\left[\begin{array}{ccc}{\nu }_{D,u}^{2}& {\nu }_{D,u}{\nu }_{F,u}{\phi }_{DF,u}& {\nu }_{D,u}{\nu }_{X,u}{\phi }_{DX,u}\\ {\nu }_{D,u}{\nu }_{F,u}{\phi }_{DF,u}& {\nu }_{F,u}^{2}& {\nu }_{F,u}{\nu }_{X,u}{\phi }_{FX,u}\\ {\nu }_{D,u}{\nu }_{X,u}{\phi }_{DX,u}& {\nu }_{F,u}{\nu }_{X,u}{\phi }_{FX,u}& {\nu }_{X,u}^{2}\end{array}\right],{\sum }_{b}=\left[\begin{array}{ccc}{\nu }_{D,d}^{2}& {\nu }_{D,d}{\nu }_{F,d}{\phi }_{DF,d}& {\nu }_{D,d}{\nu }_{X,d}{\phi }_{DX,d}\\ {\nu }_{D,d}{\nu }_{F,d}{\phi }_{DF,d}& {\nu }_{F,d}^{2}& {\nu }_{F,d}{\nu }_{X,d}{\phi }_{FX,d}\\ {\nu }_{D,d}{\nu }_{X,d}{\phi }_{DX,d}& {\nu }_{F,d}{\nu }_{X,d}{\phi }_{FX,d}& {\nu }_{X,d}^{2}\end{array}\right].$$

By applying the parameter set of the model, $\Theta$, the log-likelihood function of daily return can be obtained by using the following equation.

39 $${L}_{Ret}\left(\Theta \right)=\mathrm{log}\left\{\prod_{t=1}^{T}{f}^{\mathbb{P}}\left[{N}_{u}\left(t\right)={n}_{u}\right]\cdot {f}^{\mathbb{P}}\left[{N}_{d}\left(t\right)={n}_{d}\right]\cdot {f}^{\mathbb{P}}\left[\left.\widetilde{R}\left(t\right)\right|{N}_{u}\left(t\right)={n}_{u},{N}_{d}\left(t\right)={n}_{d}\right]\right\} =\sum_{t=1}^{T}\left\{\begin{array}{c}-{\lambda }_{u}+{n}_{u}ln{\lambda }_{u}-ln{n}_{u}!-{\lambda }_{d}+{n}_{d}ln{\lambda }_{d}-ln{n}_{d}!-\frac{3}{2}ln2\pi -\frac{1}{2}ln\left|{\Sigma }_{1}+{n}_{u}{\Sigma }_{u}+{n}_{d}{\Sigma }_{d}\right|\\ -\frac{1}{2}{\left[\widetilde{R}\left(t\right)-\left(\widetilde{\mu }+{n}_{u}\widetilde{{\theta }_{u}}+{n}_{d}\widetilde{{\theta }_{d}}\right)\right]}^{\mathrm{^{\prime}}}{\left({\sum }_{1}+{n}_{u}{\sum }_{u}+{n}_{d}{\sum }_{d}\right)}^{-1}\left[\widetilde{R}\left(t\right)-\left(\widetilde{\mu }+{n}_{u}\widetilde{{\theta }_{u}}+{n}_{d}\widetilde{{\theta }_{d}}\right)\right]\end{array}\right\},$$

where $${f}^{\mathbb{P}}\left[{N}_{u}\left(t\right)={n}_{u}\right]=\frac{{\lambda }_{u}^{{n}_{u}}\mathrm{exp}\left(-{\lambda }_{u}\right)}{{n}_{u}!}$$ and $${f}^{\mathbb{P}}\left[{N}_{d}\left(t\right)={n}_{d}\right]=\frac{{\lambda }_{d}^{{n}_{d}}\mathrm{exp}\left(-{\lambda }_{d}\right)}{{n}_{d}!}$$.

Consistent with Bakshi et al. (2008), Christoffersen et al. (2012), and Ornthanalai (2014), the relative implied volatility pricing errors are used to compute the log-likelihood of option prices. In particular, the price of a currency option is quoted as implied volatility. With the parameter set $\Theta$, the relative implied volatility error for option $n$ is obtained by using the following equation.

40 $${e}_{\Theta ,n}=\frac{I{V}_{\Theta ,n}^{Model}-I{V}_{\Theta ,n}^{MKT}}{I{V}_{\Theta ,n}^{MKT}},$$

where ${e}_{\Theta ,K}$ is the relative implied volatility error, and $I{V}_{\Theta ,n}^{Model}$ and $I{V}_{\Theta ,n}^{MKT}$ are the implied volatility of the model and market, respectively.

We assume that the relative implied volatility error has a normal distribution of ${e}_{\Theta ,n}\sim Normal\left(0,{\sigma }_{e}^{2}\right)$, and that it does not correlate with shocks in returns; therefore, the log-likelihood function of option prices is obtained by using the following equation:

41 $${L}_{Opt}\left(\Theta \right)=-\frac{1}{2}{\sum }_{n=1}^{{T}_{Opt}}\left[\mathrm{log}\left(2\pi {\sigma }_{e}^{2}\right)+\frac{{e}_{\Theta ,n}^{2}}{{\sigma }_{e}^{2}}\right].$$

#### EM algorithm

The financial literature has widely reported the existence of volatility clusters, a phenomenon where high volatility periods follow initial periods of high volatility. These clusters are often associated with market turbulence and significant price movements; however, Li et al. (2021) noted that the timing and intensity of these clusters and the occurrence of systematic jumps are difficult to predict in advance. To address parameters that are not directly observable, such as the intensity of jumps and volatility clusters, the EM algorithm has become the most common method employed by financial researchers and practitioners (Kou et al. 2014). This algorithm is a two-step process that involves computing expected values and maximizing the likelihood of observed data. The EM algorithm’s expectation step is responsible for calculating the probability of jumps, while the maximization step computes the distributional parameters that maximize the likelihood of observed data. The two steps of the EM algorithm comprise the following.

Taking expectation of the complete log-likelihood function is calculated (*j* − 1) times for the observed data. ${\mathbb{E}}\left[\left.\Theta \right|{\Theta }^{\left(j-1\right)}\right]$ is denoted as the expectation of the complete log-likelihood function, obtained using the following equation.

42 $${\mathbb{E}}\left[\Theta |{\Theta }^{\left(j-1\right)}\right]={\mathbb{E}}\left[{L}_{Ret}\left(\Theta |{\widetilde{R}}_{1:T},{N}_{u,1:T},{N}_{d,1:T}\right)|{\widetilde{R}}_{1:T},{N}_{u}\left(t\right),{N}_{d}\left(t\right)\right] ={\sum }_{{n}_{u}=0}^{\infty }{\sum }_{{n}_{d}=0}^{\infty }{L}_{Ret}\left(\Theta |{\widetilde{R}}_{1:T},{N}_{u,1:T},{N}_{d,1:T}\right)\cdot P\left({N}_{u}\left(t\right)={n}_{u},{N}_{d}\left(t\right)={n}_{d}|{\widetilde{R}}_{1:T},{\Theta }^{\left(j-1\right)}\right),$$

where $P\left(\left.{N}_{u}\left(t\right)={n}_{u},{N}_{d}\left(t\right)={n}_{d}\right|{\widetilde{R}}_{1:T},{\Theta }^{\left(j-1\right)}\right)$ is the posterior probability of jump intensity for each iteration of the calculation, determined using the following equation.

43 $$P\left(\left.{N}_{u}\left(t\right)={n}_{u}, {N}_{d}\left(t\right)={n}_{d}\right|{\widetilde{R}}_{1:T},{\Theta }^{\left(j-1\right)}\right) =\frac{P\left({N}_{u}\left(t\right)={n}_{u}\right)\cdot P\left({N}_{d}\left(t\right)={n}_{d}\right)\cdot {L}_{Ret}\left(\left.\Theta \right|{\widetilde{R}}_{1:T},{\Theta }^{\left(j-1\right)}\right)}{{\iint }_{-\infty }^{\infty }P\left({N}_{u}\left(s\right)={n}_{u}\right)\cdot P\left({N}_{d}\left(s\right)={n}_{d}\right)\cdot {L}_{Ret}\left(\left.\Theta \right|{\widetilde{R}}_{1:T},{\Theta }^{\left(j-1\right)}\right)ds}.$$

As a set of parameters that maximize ${\mathbb{E}}\left[\left.\Theta \right|{\Theta }^{\left(j-1\right)}\right]$ is identified and the calibrated parameter set $\Theta$ is calculated *j*th times. Let ${\Theta }^{ (j)}$ be the initial parameters and repeat the expectation step to obtain the parameter set (*j* + 1) times. The two steps are repeated until the parameters converge.

## Empirical results

The empirical results were calibrated from three currency pairs: EUR/USD, GBP/USD, and JPY/USD. The sample period is from October 1, 2003 (i.e., the first trading date of currency options) to December 31, 2020. Because of data limitations, additional currency pairs could not be used. For example, quoting ceased for the Canadian and Australian LIBORs in 2010, and specific currency options with underlying exchange rates involving currencies from the U.S. and emerging countries are not quoted.

### Data description

This study’s empirical data comprise the daily spot exchange rates for EUR/USD, GBP/USD, and JPY/USD; the daily 1-, 3-, 6-, and 12-month LIBORs for the U.S., the EU, the United Kingdom (U.K.), and Japan; and the daily 1-, 3-, 6-, and 12-month option prices with delta-neutral straddle (ATMV), 10-delta risk reversals (RR10), 25-delta risk reversals (RR25), 10-delta butterfly spreads (BF10), and 25-delta butterfly spreads (BF25) for EUR/USD, GBP/USD, and JPY/USD. The spot exchange rates and LIBORs were downloaded from the Federal Reserve Economic Data database. The option prices were obtained from the Bloomberg database. Table 1 presents the descriptive statistics, revealing that the LIBOR returns for the U.S., EU, and U.K. at four different maturities are all negative. The descriptive statistics reveal that the LIBOR returns for the U.S., EU, and UK at four different maturities are all negative, indicating that the interest rates in these three regions are decreasing in the long term. However, the means of the LIBOR returns for JP are positive but close to zero, indicating that the interest rates are highly stable. Furthermore, the countries with the fastest to slowest decline rates are Japan, the U.S., Europe, and the U.K., respectively. Among the three different exchange rates, only EUR/USD and JPY/USD have a positive mean of log-returns, while GBP/USD has a negative mean of log-returns, illustrating that EUR and JPY appreciate against the USD in the longer term, while the GBP depreciates against the USD. Except for Japan, the volatility ($\sigma$) of the LIBOR returns in the U.S., Europe, and the U.K. is larger at 1- and 12-month, and smaller at 3- and 6-month, while the volatility of the log-returns on the exchange rate is larger than the LIBOR returns’ volatility.

Because Bloomberg quotes the prices of the currency options as implied volatility (IV), the daily closed IV from the five options above (RR10, RR25, ATMV, BF25, and BF10) must be extracted with the method proposed by Carr and Wu (2007). When a currency option has a maturity $T$, the IV of 50-delta is equal to the IV of a delta-neutral straddle quote. The IVs of a 25-delta call option ($IV\left(25c\right)$) and 25-delta put option ($IV\left(25p\right)$) are calculated using the RR10 quote and BF10 quote by applying Eq. (44), which is as follows:

44 $$IV\left(25c\right)=BF25+ATMV+RR25/2, IV\left(25p\right)=BF25+ATMV-RR25/2.$$

After calculating the IVs of the 10-delta, 25-delta, 50-delta, 75-delta, and 90-delta call options, the model proposed by Garman and Kohlhagen (1983) is employed to determine the daily strike prices (K) and daily option prices, for which Eqs. (45) and (46), respectively, are applied. The equations are as follows.

45 $$K={X}_{t}\cdot \mathrm{exp}\left[\left({r}_{D}-{r}_{F}+\frac{1}{2}{\sigma }^{2}\right)\tau \right],$$

46 $$C\left({X}_{t},\tau \right)=\mathrm{exp}\left[-{r}_{F}\tau \right]\cdot {X}_{t}N\left({d}_{1}\right)-\mathrm{exp}\left[-{r}_{D}\tau \right]\cdot KN\left({d}_{2}\right),$$

where the parameters correspond to the notations used by Garman and Kohlhagen (1983).

### Joint estimation results

This section compares the calibrated parameters and log-likelihood function values of the CB, CB–ISJ, CB–CSJ, and CB–CAJ models. For the CB–ISJ, CB–CSJ, and CB–CAJ models, the parameters are difficult to calibrate by maximizing the incomplete log-likelihood function with the unobservable component of the jump part. The EM algorithm is the conventional method for estimating the parameters within an unobservable jump component; the parameters of the CB model are also calibrated through a maximum likelihood estimation.

The parametric estimation results for EUR/USD, GBP/USD, and JPY/USD are presented in Tables 2, 3, and 4, respectively. From our daily data for each currency pair and each maturity, only 3 observations in the return data and 20 observations in the options data are employed to calibrate the 42 parameters. Due to limited daily observations, the daily data for each month (approximately 20 trading days) are used to calibrate the parameters. In other words, this research assumes that the parameters are constant each month for each maturity and uses approximately 60 and 100 observations of the return and option data, respectively, to calibrate the parameters.

Table 2 presents the calibrated parameters and likelihood function level for the paired LIBOR and the exchange rate (i.e., EU vs. the U.S.). Panels (a) and (b) of Table 2 show the estimation results of the parameters of EUR/USD in the continuous and the jump parts, respectively, while Panel (c) shows the results of the likelihood and LRT of each model.

In the continuous part (Panel (a)), the CB–CAJ model calibrated negative means for the domestic and foreign interest rates for four different maturities. The means of the domestic interest rate (${\mu }_{D}$) are $-$ 0.000775, $-$ 0.000083, $-$ 0.001298, and $-$ 0.000815 for 1-, 3-, 6-, and 12-month respectively, while the means of the foreign interest rate (${\mu }_{F}$) are $-$ 0.004944, $-$ 0.006107, $-$ 0.006794, and $-$ 0.011111, respectively. The estimation results show that the trend for domestic and foreign interest rates decreases in the long term, and the interest rate declines accelerate as their maturity increases. The means of the exchange rate (${\mu }_{X}$) are positive, which are 0.036623, 0.032122, 0.032705, and 0.010433, respectively, indicating the depreciation of the foreign currency (EUR) against the domestic currency (USD). According to the IRP, if the domestic interest rate declines less than the foreign interest rate, investors prefer to hold the domestic currency; therefore, the domestic currency appreciates relative to the foreign currency. In other words, the foreign currency depreciates relative to the domestic currency. Our parameter estimation results are consistent with the IRP.

The volatility of the domestic interest rate (${\sigma }_{D}$), foreign interest rate (${\sigma }_{F}$), and exchange rate (${\sigma }_{X}$) are 0.006984, 0.004120, and 0.118903, respectively. The ranking of volatility from highest to lowest is the exchange rate, domestic interest rate, and foreign interest rate. This ranking is consistent with the result in the descriptive statistics. The correlation coefficient between the domestic and foreign interest rates (${\rho }_{DF}$) is significantly positive, indicating that the central banks of the domestic and foreign countries adopt similar monetary policies. This finding is also true in reality, as the European Central Bank (ECB) and the Federal Reserve (Fed) adopted similar monetary policies in the sample periods. After the subprime crisis in 2008, the Fed adopted an accommodative monetary policy, quantitative easing (QE), and the ECB quickly adopted an accommodative monetary policy similar to QE. Most of the risk premia in the continuous part (${h}_{1,D}$, ${h}_{1,F}$, ${h}_{1,X}$) are negative, and a few are positive; however, few of the positive risk premia are significant. According to the empirical results of Dahlquist and Pénasse (2022), De Santis and Gerard (1998), Doukas et al. (1999), and Tai (2003), the risk premium of the exchange rate is a time-varying value, which may change between positive and negative; consequently, it is confirmed that our estimation results are reasonable.

In the jump part (Panel (b)), the means of the upside jump amplitude (${\theta }_{X,u}$) are 0.057719, 0.093479, 0.096152, and 0.077248 at 1–, 3–, 6–, and 12–month, respectively, all of which are significantly positive; the means of the downside jump amplitude (${\theta }_{X,d}$) are $-$ 0.065160, $-$ 0.115084, $-$ 0.113685, and $-$ 0.171123, respectively, which are significantly negative. ${\theta }_{X,u}$ and ${\theta }_{X,d}$ decrease gradually with an extension of maturity; for the same maturity, the absolute value of ${\theta }_{X,u}$ is smaller than that of ${\theta }_{X,d}$, indicating that the intensity of an upside jump is smaller than that of a downside jump when jumps occur. The significant estimation results of the means of the upside and downside jump intensity (${\theta }_{X,u}$ and ${\theta }_{X,d}$) are consistent with the economic implications of the upside and downside jumps. The standard deviation of an upside jump (${\nu }_{X,u}$) is higher than that of a downside jump (${\nu }_{X,d}$), indicating that when a jump occurs, the dispersion of the jump intensity of an upside jump is higher than that of a downside jump.

For the jump frequency ($\uplambda$), the upside jump frequencies (${\lambda }_{u}$) of CB–CAJ at 1–, 3–, 6–, and 12–month are 0.0106294, 0.096217, 0.100483, and 0.076891, respectively, and the downside jump frequencies (${\lambda }_{d}$) are 0.105518, 0.109510, 0.129113, and 0.169682. Thus, the average intervals between the occurrences of each upside jump are 9.41 ($\approx 1/\uplambda$), 9.13, 9.95, and 13.01 days, and the average interval between the occurrences of each downside jump is 9.48, 9.13, 7.75, and 5.89 days, respectively. The jump frequencies ($\uplambda$) of the CB–ISJ and CB–CSJ models assuming symmetric jumps are very close to the sum of the upside and downside jump frequencies (${\lambda }_{u}$ and ${\lambda }_{d}$) of the CB–CAJ model, which assumes asymmetric jumps, and the jump frequencies decrease gradually with an extension of maturity. Combining upside and downside jumps in terms of the mean and standard deviation of jump intensities and frequencies show that upside jumps and downside jumps have a significant difference. This result is consistent with the analysis in the introduction and demonstrates the necessity of decomposing upside and downside jumps. Most of the upside and downside jump risk premia of the exchange rates (${h}_{X,u}$ and ${h}_{X,d}$) are positive for the jump risk premium, indicating that the investors gain positive returns for undertaking the risk of a jump. Moreover, the calibrated parameters for the U.K. versus the U.S. (Table 3) and Japan versus the U.S. (Table 4) reveal similar empirical results.

Three hypotheses were constructed to compare these models and discuss the performance of each model. First, the hypothesis for comparing the performance of the CB and CB–ISJ models is as follows. The null hypothesis is that the domestic and foreign interest and exchange rates would follow the CB model. In contrast, the alternative hypothesis is that the three assets would follow the CB–ISJ model. The second and third hypotheses relate to the performance of the CB–ISJ and CB–CSJ models and that of the CB–CSJ and CB–CAJ models, respectively. Applying the parameter set $\Theta$, the observation set ${\mathbb{D}}$, the parameter space $\Omega$, and the maximized joint log-likelihood function $L\left(\Theta \in\Omega |{\mathbb{D}}\right)$, for the null hypothesis (${H}_{0}:\Theta \in {\Omega }_{0}$) against the alternative hypothesis (${H}_{1}:\Theta \in\Omega -{\Omega }_{0}$), allows us to obtain the LRT statistics using the following equation.

47 $$LRT=2\left[L\left(\Theta \in\Omega -{\Omega }_{0}|{\mathbb{D}}\right)- L\left(\Theta \in {\Omega }_{0}|{\mathbb{D}}\right)\right]\sim {x}^{2}\left(k\right),$$

where $k$ is the difference in the number of parameters between the two models. Under these null and alternative hypotheses with a large sample, the LRT statistic follows a chi-square distribution with a degree of freedom $k$. Furthermore, at a 1% significance level, the critical values of rejection are $LR{T}_{1}\ge {x}_{0.01}^{2}\left(10\right)=23.209$, $LR{T}_{2}\ge {x}_{0.01}^{2}\left(3\right)=11.345$, and $LR{T}_{3}\ge {x}_{0.01}^{2}\left(15\right)=30.578$ for the three tests.

To avoid large-sample size bias, we follow Michaelides (2021) and recalculate the LRT test’s critical values. We used a sample of 4502 observations for each currency pair in the parametric calibration. Because of the 1% significance level, the p-value must be changed from 0.01 to 0.00149;^2^ therefore, the critical values of rejection are ${LRT}_{1}\ge {x}_{0.00149}^{2}\left(10\right)=28.518$, ${LRT}_{2}\ge {x}_{0.00149}^{2}\left(3\right)=15.421$, and ${LRT}_{3}\ge {x}_{0.00149}^{2}\left(15\right)=36.512$

In Panel (c) of Tables 2, 3, and 4, the log-likelihood results for all four tested models have the following magnitude relationship, regardless of maturity: CB $<$ CB–ISJ $<$ CB–CSJ $<$ CB–CAJ. The LRT test results reveal that, despite potential large-sample bias, each of the more complex models outperforms the previous simpler model at the 1% significance level. The relationships among the four tested models are also consistent with the model assumptions. Because the CB–CAJ model has the largest LRT statistics for all currency pairs, the CB–CAJ model is the most appropriate for capturing market characteristics.

### In-sample and out-of-sample pricing performance

This section compares the in-sample and out-of-sample pricing performance of the GK, CB, CB–ISJ, CB–CSJ, and CB–CAJ models by summing the means of squares pricing errors (MSEs). The three currency pairs were tested with four maturities and five option risk levels. The MSEs are calculated by using the following equations.

48 $$MS{E}_{IN}^{i}=\frac{1}{T\cdot N}{\sum }_{t=1}^{T}{\sum }_{n=1}^{N}{\left\{Cal{l}_{i,n}^{Model}\left[{\Theta }^{i}\left(t\right),t\right]-Cal{l}_{i,n}^{Market}\left(t\right)\right\}}^{2},$$

49 $$MS{E}_{OUT}^{i}=\frac{1}{T\cdot N}{\sum }_{t=1}^{T}{\sum }_{n=1}^{N}{\left\{Cal{l}_{i,n}^{Model}\left[{\Theta }^{i}\left(t-1\right),t\right]-Cal{l}_{i,n}^{Market}\left(t\right)\right\}}^{2},$$

where $i$ is the type of currency option maturity ($i=\mathrm{1,3},\mathrm{6,12}$); $T$ is the number of available trading days ($T=4051$); $N$ is the number of available currency options at time $t$ for type $i (N=5$); $Cal{l}_{i,n}^{Model}\left[{\Theta }^{i}\left(t\right),t\right]$ is the model price calculated by the parameter set ${\Theta }^{i}\left(t\right)$ of the $n$th currency option, which has an $i$ maturity date at time $t$. Furthermore, $Cal{l}_{i,n}^{Market}\left(t\right)$ is the market price of the $n$th currency option with an $i$ maturity date at time $t$, and ${\Theta }^{i}\left(t\right)$ is the parameter set for the $n$th currency option with an $i$ maturity date at time $t$. A smaller MSE value indicates a better pricing performance of a model.

The left and right panels of Table 5 represent the in-sample and out-of-sample pricing performance of the five delta levels for the EUR/USD exchange rate options. The CB–CAJ model has the best in-sample average pricing error performance at an average of 10-delta and a 6-month average of 25-delta. For the out-of-sample results, the CB–CAJ model has the best performance of only 3-month at the 10-delta and 6-month at the 25-delta. The best pricing error performance is not obtained in other cases, but the difference from the best result is negligible. The CB–CAJ model parameters are simultaneously calibrated with bond and option market data, so the complex models do not outperform the simple ones. Although the CB–CAJ model cannot obtain the best pricing performance in all cases, its pricing error differs little from the best model, providing a broader scope for the economic implications of the currency risk factors. Tables 6 and 7 present similar empirical results for the GBP/USD and JPY/USD options.

In previous research, Park (2016) used asymmetric volatility and jump to price volatility index (VIX) derivatives and applied the root mean square error to evaluate the pricing performance, testing the model using long and short positions of VIX futures, call options, and put options. However, the asymmetric volatility and jump model fail to outperform the upside jump and no jump models for futures (long position) and call options. Some researchers, such as Ornthanalai (2014), Chang et al. (2019), and Cheng et al. (2020), used GARCH family models to analyze the asymmetric volatility and jumps in stock options. Their empirical results indicate that the upside and downside jump components asymmetrically affect returns. Furthermore, their jump model’s volatility level does not significantly increase after days with moderately positive returns. This finding may explain why the CB–CAJ model does not have the smallest MSEs for each maturity and delta level.

**Fig. 2:**
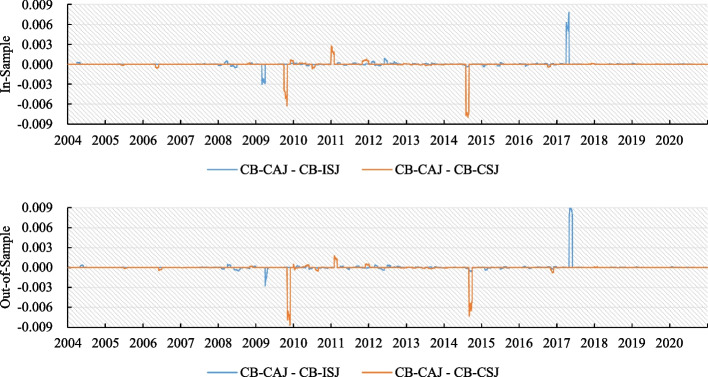
Time series of the differences in pricing error between models. This figure depicts the time series of the difference of pricing errors between the models for EUR/USD with a 12-month maturity and 50 delta level (at the money) from 2004/1/1 to 2020/12/31. “CB–CAJ − CB–ISJ” indicates the value of the pricing error of the CB–CAJ model minus the CB–ISJ model, and “CB–CAJ − CB–CSJ” indicates the value of the pricing error of the CB–CAJ model minus the CB–CSJ model

The pricing performances in Tables 5, 6, and 7 indicate that the CB–CAJ model did not exhibit superior performance in all maturity and delta levels. Although CB–CAJ is not the best-performing model, it is not the worst. The CB–CAJ model’s pricing errors are close to those of the two best-performing models. Among the pricing performance of the three currencies, the better-performing models are CB–ISJ, CB–CSJ, and CB–CAJ. To illustrate the differences in the performance of these three models, we plotted the time series (Fig. 2) of EUR/USD at a maturity of 12-month and the delta level of 50 (at the money). The four lines in Fig. 2 indicate the differences between the CB–CAJ and the other two models. A value larger (smaller) than zero indicates that the CB–CAJ model underperforms (outperforms) another model. The means of the pricing errors of the CB–CAJ model do not reflect superior performance; however, on some specific dates, this model still outperformed the other models. Option pricing errors are one of the criteria for evaluating models; however, our proposed CB–CAJ model outperforms the other models when we use FX returns and options market data for a comprehensive assessment.

### Risk premium analysis

This study aims to capture the time-varying risk premium, especially the upside and downside jump risk premia. Moreover, the CB–CAJ model can obtain the continuous, upside, and downside jump risk premia. According to IRP, the interest rate differential between domestic and foreign countries is equal to the differential between the forward and spot exchange rates. When shock occurs, the market price of the interest and exchange rates cannot satisfy the IRP, and jumps occur due to the arbitrage opportunities that are grabbed by the traders. Therefore, the upside and downside jump risk premia are tested for the periods when different economic events occur and explore whether the jump risk premia can explain the exchange rate returns. Following Aliber (1973), Balke and Wohar (1998), Chinn (2006), Cosandier and Lang (1981), and Du et al. (2018), this research uses $\left(FWD/SPOT-1\right)$ as the return of the exchange rate and proposes the regression as Eq. (50).

50 $$\left(\frac{{FWD}_{t}^{Cur,i}}{{SPOT}_{t}^{Cur}}-1\right)=\alpha +{\beta }_{1}\left({R}_{D,t}^{i}-{R}_{F,t}^{i}\right)+{\beta }_{2}{h}_{1,X,t}^{i}+{\beta }_{3}{h}_{2,X,u,t}^{i}+{\beta }_{4}{h}_{2,X,d,t}^{i}+{\varepsilon }_{t},$$

where

$$Cur=\left\{EURUSD, GBPUSD, JPYUSD\right\},i=\left\{1\mathrm{m}, 3m, 6m, 12m\right\},$$

$Cur$ is denoted by the currency pair of the contract, $i$ is denoted by the duration of the contract, ${FWD}_{t}^{Cur,i}$ is denoted by the forward exchange rate (indirect quoted rates with the U.S. as the domestic country) for the duration of $i$ concerning the currency pair $Cur$ at time $t$, and ${SPOT}_{t}^{Cur}$ is denoted by the spot exchange rate (indirect quoted rates with the U.S. as a domestic country) regarding the currency pair $Cur$ at time $t$. ${R}_{D,t}^{i}$ and ${R}_{F,t}^{i}$ are denoted by the interest rates for the duration of $i$ at time $t$ in the domestic and foreign countries, and ${h}_{1,X,t}^{i}$, ${h}_{2,X,u,t}^{i}$, and ${h}_{2,X,d,t}^{i}$ are denoted by the risk premia of the exchange rate in the continuous, upside, and downside jump for the duration of $i$ at time $t$, respectively.

Extensive tests were conducted, and Table 8 shows the four periods when the EUR/USD results were relatively significant. The years 2008 and 2009 were when the subprime crisis occurred, 2011 was when the EU debt crisis occurred, and 2018 was when the trade war between the U.S. and China took place. The coefficient of interest spread, ${\beta }_{1}$, is significant in most cases, indicating that the interest spread can explain the return of the exchange rate. Comparing the significance level of 2008 and 2009 among the upside (${\beta }_{3}$) and downside (${\beta }_{4}$) jump risk premia shows that the significance level was higher in 2008 than in 2009. This finding implies that although the subprime crisis still influenced the exchange rate in 2009, the jump frequency and amplitude in the exchange rate diminished, and the jump risk premia that could be obtained became less significant. Comparing the coefficient of the upside and downside jump risk premia in 2008 indicates that the upside jump risk premium is 1% significant in the maturities of 3-, 6- and 12-month; in contrast, the downside jump risk premium is 1% significant only in the maturity of 12-month. When there is an upside jump in the exchange rate, the rate rises, representing an appreciation of the USD against foreign currencies. The high significance level of upside jumps indicates that investors prefer to hold the USD to hedge against risk. In contrast, at 12-month, upside jumps are equally significant as downside jumps, suggesting more divergence in investors’ preferences for currency holdings in the long run.

In the results of 2011, only the jump risk premium is significant in the 6- and 12-month, indicating that the overall market prefers to hold euros, which may be due to the willingness of importers and exporters to take more significant market risks for medium- to long-term currency demands.

In the results of 2018, 1-, 3- and 6-month are more significant for the downside jump risk premium, while 12-month is more significant for the upside jump risk premium. This result indicates that investors prefer to hold EUR in the short- to medium-term and USD in the long-term. This empirical result demonstrates the strength and weakness of the relationship between the EUR and the USD at different maturities.

## Conclusions

Regarding the models proposed by Garman and Kohlhagen (1983), Amin and Jarrow (1991), and Chuang et al. (2020), this research proposes a model—the CB–CAJ—that simultaneously considers a correlated asymmetric jump in the three rates. This model fills in the gap of the asymmetric jump model in the currency option pricing models. Under the assumption of the CB–CAJ model, this study applies the martingale method to derive the closed-form of the currency call options and uses the EM algorithm to calibrate the parameters. From October 1, 2003, onward, the research period spans events that involve financial crises, political risks, monetary policies, and global pandemic threats; all of these developments can influence interest and exchange rates.

The empirical study considers the correlated upside jump and downside jump risk premia in currency options with four maturities (1-, 3-, 6-, and 12-month), five delta levels (10-, 25-, 50-, 75-, and 90-delta), and three currency pairs (EUR/USD, GBP/USD, and JPY/USD) in the past 17 years.

The results of the parametric estimation reveal that the CB–CAJ model has the largest log-likelihood in all four maturities and the three currency pairs, suggesting that the CB–CAJ model favorably fits the bond and options markets. Comparing the LRT results indicates that the CB–CAJ model outperforms the CB, CB–ISJ, and CB–CSJ models in capturing the dynamic movements of the interest and exchange rates in all three currency pairs. The jump-related parameters demonstrate that the CB–CAJ model captures the frequency and amplitude of asymmetric jumps and effectively supports the analysis of the risk in the upside and downside jumps for a given period. Furthermore, the presence of asymmetric jumps is evidenced by the significant difference in parameters between the upside and downside jumps.

The results relating to in- and out-of-sample pricing errors indicate that although the CB–CAJ model cannot obtain the best pricing performance in all cases, its pricing error differs very little from the best model, providing a broader scope for the economic implications of the currency risk factors.

Furthermore, in the analysis of risk premia, the upside and downside jump risk premia can explain investors’ preferences for currency holdings during periods of subprime crises, EU debt crises, and other economic events. Additionally, a significant difference in the explanatory power of the jump risk premia was observed between the upside and downside across economic events. The CB–CAJ model provides an extended scope for the economic interpretations of the factors that drive currency risk premia and helps explain the exchange rate fluctuations.

## Data Availability

Data is available from Federal Reserve Economic Data (FRED) and Bloomberg.

## Appendix 1: Dynamic process of domestic and foreign interest rates

### Dynamic process of domestic interest rate

By assuming the dynamic process of domestic forward interest rate as Eq. (1), it has:

51 $${f}_{D}\left(t,{t}_{\beta }\right)={f}_{D}\left(0,{t}_{\beta }\right)+{\int }_{0}^{t}{\alpha }_{D} ({t}_{\alpha },{t}_{\beta })d{t}_{\alpha }+{\int }_{0}^{t}{\sigma }_{D}\left({t}_{\alpha },{t}_{\beta }\right)d{W}_{D}^{\mathbb{P}}\left({t}_{\alpha }\right)+{\int }_{0}^{t}{Y}_{D,u}\left({t}_{\alpha },{t}_{\beta }\right)d{N}_{u}\left({t}_{\alpha }\right)+{\int }_{0}^{t}{Y}_{D,d}\left({t}_{\alpha },{t}_{\beta }\right)d{N}_{d}\left({t}_{\alpha }\right).$$

The domestic bond price in units of the domestic currency can be written as follows:

52 $${P}_{D}\left(t,T\right)=\mathrm{exp}\left[-{\int }_{t}^{T}{f}_{D}\left(t,{t}_{\beta }\right)d{t}_{\beta }\right].$$

By applying the stochastic Fubini theorem and switching the order of integration, it has:

53 $${\int }_{t}^{T}{f}_{D}\left(t,{t}_{\beta }\right)d{t}_{\beta }={\int }_{0}^{T}{f}_{D}\left(0,{t}_{\beta }\right)d{t}_{\beta }+{\int }_{0}^{t}{\int }_{{t}_{\alpha }}^{T}{\alpha }_{D}\left({t}_{\alpha },{t}_{\beta }\right)d{t}_{\beta }d{t}_{\alpha }+{\int }_{0}^{t}{\int }_{{t}_{\alpha }}^{T}{\sigma }_{D}\left({t}_{\alpha },{t}_{\beta }\right)d{t}_{\beta }d{W}_{D}^{\mathbb{P}}\left({t}_{\alpha }\right)+{\int }_{0}^{t}{\int }_{{t}_{\alpha }}^{T}{Y}_{D,u}\left({t}_{\alpha },{t}_{\beta }\right)d{t}_{\beta }d{N}_{u}\left({t}_{\alpha }\right)+{\int }_{0}^{t}{\int }_{{t}_{\alpha }}^{T}{Y}_{D,d}\left({t}_{\alpha },{t}_{\beta }\right)d{t}_{\beta }d{N}_{d}\left({t}_{\alpha }\right)-{\int }_{0}^{t}{f}_{D}\left(0,{t}_{\beta }\right)d{t}_{\beta }-{\int }_{0}^{t}{\int }_{{t}_{\alpha }}^{t}{\alpha }_{D}\left({t}_{\alpha },{t}_{\beta }\right)d{t}_{\beta }d{t}_{\alpha }-{\int }_{0}^{t}{\int }_{{t}_{\alpha }}^{t}{\sigma }_{D}\left({t}_{\alpha },{t}_{\beta }\right)d{t}_{\beta }d{W}_{D}^{\mathbb{P}}\left({t}_{\alpha }\right) -{\int }_{0}^{t}{\int }_{{t}_{\alpha }}^{t}{Y}_{D,u}\left({t}_{\alpha },{t}_{\beta }\right)d{t}_{\beta }d{N}_{u}\left({t}_{\alpha }\right)-{\int }_{0}^{t}{\int }_{{t}_{\alpha }}^{t}{Y}_{D,d}\left({t}_{\alpha },{t}_{\beta }\right)d{t}_{\beta }d{N}_{d}\left({t}_{\alpha }\right).$$

Let ${\alpha }_{D}^{*}\left(t,T\right)={\int }_{t}^{T}{\alpha }_{D}\left(t,{t}_{\beta }\right)d{t}_{\beta }$, ${\sigma }_{D}^{*}\left(t,T\right)={\int }_{t}^{T}{\sigma }_{D}\left(t,{t}_{\beta }\right)d{t}_{\beta }$, ${Y}_{D,u}^{*}\left(t,T\right)={\int }_{t}^{T}{Y}_{D,u}\left(t,{t}_{\beta }\right)d{t}_{\beta }$, and ${Y}_{D,d}^{*}\left(t,T\right)={\int }_{t}^{T}{Y}_{D,d}\left(t,{t}_{\beta }\right)d{t}_{\beta }$, Eq. (52) can be written as follows:

54 $${P}_{D}\left(t,T\right)=\mathrm{exp}\left[{\int }_{0}^{t}{r}_{D}\left({t}_{\alpha }\right)d{t}_{\alpha }-{\int }_{0}^{T}{f}_{D}\left(0,{t}_{\alpha }\right)d{t}_{\alpha }-{\int }_{0}^{t}{\alpha }_{D}^{*}\left({t}_{\alpha },T\right)d{t}_{\alpha }-{\int }_{0}^{t}{\sigma }_{D}^{*}\left({t}_{\alpha },T\right)d{W}_{D}^{\mathbb{P}}\left({t}_{\alpha }\right)-{\int }_{0}^{t}{Y}_{D,u}^{*}\left({t}_{\alpha },T\right)d{N}_{u}\left({t}_{\alpha }\right)-{\int }_{0}^{t}{Y}_{D,d}^{*}\left({t}_{\alpha },T\right)d{N}_{d}\left({t}_{\alpha }\right)\right].$$

Let $A\left(t,T\right)=-{\int }_{t}^{T}{f}_{D}\left(t,{t}_{\alpha }\right)d{t}_{\alpha }$ and ${P}_{D}\left(t,T\right)=\mathrm{exp}\left[A\left(t,T\right)\right]$, by Itô formula for jump-diffusion processes, it has:

55 $$d{P}_{D}\left(t,T\right)=\frac{\partial {P}_{D}\left(t,T\right)}{\partial t}dt+\frac{\partial {P}_{D}\left(t,T\right)}{\partial A\left(t,T\right)}dA\left(t,T\right)+\frac{1}{2}\frac{{\partial }^{2}{P}_{D}\left(t,T\right)}{\partial {A}^{2}\left(t,T\right)}{\left[dA\left(t,T\right)\right]}^{2}+\left\{{P}_{D}\left({A}_{t-}+\Delta {A}_{t}\right)-{P}_{D}\left({A}_{t-}\right)\right\} ={P}_{D}\left(t,T\right)\left\{\left[{r}_{D}\left(t\right)-{\alpha }_{D}^{*}\left(t,T\right)+\frac{1}{2}{{\sigma }_{D}^{*}}^{2}\left(t,T\right)\right]dt-{\sigma }_{D}^{*}\left(t,T\right)d{W}_{D}^{\mathbb{P}}\left(t\right)\right\}+\left\{\mathrm{exp}\left[A\left(t,T\right)-{Y}_{D,u}^{*}\left(t,T\right)\right]-\mathrm{exp}\left[A\left(t,T\right)\right]\right\}d{N}_{u}\left(t\right)+\left\{\mathrm{exp}\left[A\left(t,T\right)-{Y}_{D,d}^{*}\left(t,T\right)\right]-\mathrm{exp}\left[A\left(t,T\right)\right]\right\}d{N}_{d}\left(t\right).$$

Therefore, it has:

56 $$\frac{d{P}_{D}\left(t,T\right)}{{P}_{D}\left(t,T\right)}=\left[{r}_{D}\left(t\right)-{\alpha }_{D}^{*}\left(t,T\right)+\frac{1}{2}{{\sigma }_{D}^{*}}^{2}\left(t,T\right)\right]dt-{\sigma }_{D}^{*}\left(t,T\right)d{W}_{D}^{\mathbb{P}}\left(t\right)+\left\{\mathrm{exp}\left[-{Y}_{D,u}^{*}\left(t,T\right)\right]-1\right\}d{N}_{u}\left(t\right)+\left\{\mathrm{exp}\left[-{Y}_{D,d}^{*}\left(t,T\right)\right]-1\right\}d{N}_{d}\left(t\right),$$

57 $${P}_{D}\left(t,T\right)={P}_{D}\left(0,T\right)\cdot \mathrm{exp}\left\{{\int }_{0}^{t}\left[{r}_{D}\left({t}_{\alpha }\right)-{\alpha }_{D}^{*}\left({t}_{\alpha },T\right)\right]d{t}_{\alpha }-{\int }_{0}^{t}{\sigma }_{D}^{*}\left({t}_{\alpha },T\right)d{W}_{D}^{\mathbb{P}}\left({t}_{\alpha }\right)\right\}\cdot \mathrm{exp}\left[-{Y}_{D,u}^{*}\left(t,T\right)d{N}_{u}\left(t\right)\right]\cdot \mathrm{exp}\left[-{Y}_{D,d}^{*}\left(t,T\right)d{N}_{d}\left(t\right)\right].$$

The jump size of domestic upside jump and downside jump are assumed to be random numbers, which follow the normal distribution, ${Y}_{D,u}^{*}\left(t,T\right)\sim Normal\left({\theta }_{D,u},{\nu }_{D,u}^{2}\right)$ and ${Y}_{D,d}^{*}\left(t,T\right)\sim Normal\left({\theta }_{D,d},{\nu }_{D,\mathrm{d}}^{2}\right)$. Therefore, the dynamic process of domestic zero-coupon bond will be

58 $$\frac{d{P}_{D}\left(t,T\right)}{{P}_{D}\left(t,T\right)}=\left[{r}_{D}\left(t\right)-{\alpha }_{D}^{*}\left(t,T\right)+\frac{1}{2}{{\sigma }_{D}^{*}}^{2}\left(t,T\right)\right]dt-{\sigma }_{D}^{*}\left(t,T\right)d{W}_{D}^{\mathbb{P}}\left(t\right)+\left[\mathrm{exp}\left(-{Y}_{D,u}^{*}\right)-1\right]d{N}_{u}\left(t\right)+\left[\mathrm{exp}\left(-{Y}_{D,d}^{*}\right)-1\right]d{N}_{d}\left(t\right),$$

59 $${P}_{D}\left(t,T\right)={P}_{D}\left(0,T\right)\cdot \mathrm{exp}\left\{{\int }_{0}^{t}\left[{r}_{D}\left({t}_{\alpha }\right)-{\alpha }_{D}^{*}\left({t}_{\alpha },T\right)\right]d{t}_{\alpha }-{\int }_{0}^{t}{\sigma }_{D}^{*}\left({t}_{\alpha },T\right)d{W}_{D}^{\mathbb{P}}\left({t}_{\alpha }\right)\right\}\cdot \mathrm{exp}\left[-{Y}_{D,u}^{*}d{N}_{u}\left(t\right)\right]\cdot \mathrm{exp}\left[-{Y}_{D,d}^{*}d{N}_{d}\left(t\right)\right],$$

60 $$d\mathrm{ln}{P}_{D}\left(t,T\right)=\left[{r}_{D}\left(t\right)-{\alpha }_{D}^{*}\left(t,T\right)\right]dt-{\sigma }_{D}^{*}\left(t,T\right)d{W}_{D}^{\mathbb{P}}\left(t\right)-{Y}_{D,u}^{*}d{N}_{u}\left(t\right)-{Y}_{D,d}^{*}d{N}_{d}\left(t\right).$$

The moment generating function (MGF) of the compound Poisson process ${J}_{D,u}={Y}_{D,u}^{*}d{N}_{u}\left(t\right)$ with parameter ${h}_{D,\mathrm{u}}$ is

61 $${M}_{{J}_{D,u}}\left({h}_{D,u}\right)={\mathbb{E}}\left[\mathrm{exp}\left({h}_{D,u}\cdot {Y}_{D,u}^{*}d{N}_{u}\left(t\right)\right)\right]=\mathrm{exp}\left\{{\lambda }_{u}dt\left[{M}_{{Y}_{D,u}^{*}}\left({h}_{D,u}\right)-1\right]\right\},$$

where ${M}_{{Y}_{D,u}^{*}}\left({h}_{D,u}\right)=\mathit{exp}\left({h}_{D,u}{\theta }_{D,u}+\frac{1}{2}{h}_{D,u}^{2}{\nu }_{D,u}^{2}\right)$ is the MGF of the distribution $Normal\left({\theta }_{D,u},{\nu }_{D,u}^{2}\right)$. Similarly, the MGF of the compound Poisson process ${J}_{D,d}={Y}_{D,d}^{*}d{N}_{d}\left(t\right)$ with parameter ${h}_{D,d}$ is

62 $${M}_{{J}_{D,d}}\left({h}_{D,d}\right)={\mathbb{E}}\left[\mathrm{exp}\left({h}_{D,d}\cdot {Y}_{D,d}^{*}d{N}_{d}\left(t\right)\right)\right]=\mathrm{exp}\left\{{\lambda }_{d}dt\left[{M}_{{Y}_{D,d}^{*}}\left({h}_{D,d}\right)-1\right]\right\},$$

where ${M}_{{Y}_{D,d}^{*}}\left({h}_{D,d}\right)=\mathrm{exp}\left({h}_{D,d}{\theta }_{D,d}+\frac{1}{2}{h}_{D,d}^{2}{\nu }_{D,d}^{2}\right)$ is the MGF of the distribution $Normal\left({\theta }_{D,d},{\nu }_{D,d}^{2}\right)$.

The Radon–Nikodým derivative are discussed in continuous part and jump part separately. By employing the Esscher transform developed by Gerber and Shiu (1994), Gerber and Shiu (1996), and Lian et al. (2021) to select the martingale pricing measure for domestic zero-coupon bond, the Radon–Nikodým derivative of the Esscher transform is given by:

63 $${\xi }^{{h}_{D}}\left(t\right)=\left.\frac{d{\mathbb{Q}}^{{h}_{D}}}{d{\mathbb{P}}}\right|\mathcal{F}\left(t\right)={\Lambda }_{t}^{{C}_{D}}\times {\Lambda }_{t}^{{J}_{D}}$$

64 $$=\frac{\mathrm{exp}\left\{{h}_{1,D}{\sigma }_{D}^{*}\left(t,T\right)d{W}_{D}^{\mathbb{P}}\left(t\right)\right\}}{{\mathbb{E}}\left\{\mathrm{exp}\left[{h}_{1,D}{\sigma }_{D}^{*}\left(t,T\right)d{W}_{D}^{\mathbb{P}}\left(t\right)\right]\right\}}\cdot \frac{\mathrm{exp}\left\{{h}_{D,u}\cdot {Y}_{D,u}^{*}d{N}_{u}\left(t\right)\right\}}{{\mathbb{E}}\left\{\mathrm{exp}\left[{h}_{D,u}\cdot {Y}_{D,u}^{*}d{N}_{u}\left(t\right)\right]\right\}}\cdot \frac{\mathrm{exp}\left\{{h}_{D,d}\cdot {Y}_{D,d}^{*}d{N}_{d}\left(t\right)\right\}}{{\mathbb{E}}\left\{\mathrm{exp}\left[{h}_{D,d}\cdot {Y}_{D,d}^{*}d{N}_{d}\left(t\right)\right]\right\}}$$

65 $$=\mathrm{exp}\left[{h}_{1,D}{\sigma }_{D}^{*}\left(t,T\right)d{W}_{D}^{\mathbb{P}}\left(t\right)-\frac{1}{2}{h}_{1,D}^{2}{{\sigma }_{D}^{*}}^{2}\left(t,T\right)dt\right]\cdot \mathrm{exp}\left\{{h}_{D,u}\cdot {Y}_{D,u}^{*}d{N}_{u}\left(t\right)-{\lambda }_{u}dt\left[\mathrm{exp}\left({h}_{D,u}{\theta }_{D,u}+\frac{1}{2}{h}_{D,u}^{2}{\nu }_{D,u}^{2}\right)-1\right]\right\}\cdot \mathrm{exp}\left\{{h}_{D,d}\cdot {Y}_{D,d}^{*}d{N}_{d}\left(t\right)-{\lambda }_{d}dt\left[\mathrm{exp}\left({h}_{D,d}{\theta }_{D,d}+\frac{1}{2}{h}_{D,d}^{2}{\nu }_{D,d}^{2}\right)-1\right]\right\}.$$

The distributions of jump size and jump intensity under ${\mathbb{Q}}$ measure are still need to be found. For the jump part, it has

66 $${\Lambda }_{t}^{{J}_{D}}=\left.\frac{d{\mathbb{Q}}^{{J}_{D}}}{d{\mathbb{P}}^{{J}_{D}}}\right|\mathcal{F}\left(t\right)=\frac{\mathrm{exp}\left\{{h}_{D,u}\cdot {Y}_{D,u}^{*}d{N}_{u}\left(t\right)\right\}}{{\mathbb{E}}\left\{\mathrm{exp}\left[{h}_{D,u}\cdot {Y}_{D,u}^{*}d{N}_{u}\left(t\right)\right]\right\}}\cdot \frac{\mathrm{exp}\left[{h}_{D,d}\cdot {Y}_{D,d}^{*}d{N}_{d}\left(t\right)\right]}{{\mathbb{E}}^{\mathbb{P}}\left\{\mathrm{exp}\left[{h}_{D,d}\cdot {Y}_{D,d}^{*}d{N}_{d}\left(t\right)\right]\right\}},$$

67 $$d{\mathbb{Q}}^{{J}_{D}}=\frac{\mathrm{exp}\left[{h}_{D,u}\cdot {Y}_{D,u}^{*}d{N}_{u}\left(t\right)\right]}{{\mathbb{E}}^{\mathbb{P}}\left\{\mathrm{exp}\left[{h}_{D,u}\cdot {Y}_{D,u}^{*}d{N}_{u}\left(t\right)\right]\right\}}\cdot \frac{\mathrm{exp}\left[{h}_{D,d}\cdot {Y}_{D,d}^{*}d{N}_{d}\left(t\right)\right]}{{\mathbb{E}}^{\mathbb{P}}\left\{\mathrm{exp}\left[{h}_{D,d}\cdot {Y}_{D,d}^{*}d{N}_{d}\left(t\right)\right]\right\}}\cdot d{\mathbb{P}}^{{J}_{D}}$$

68 $$={\sum }_{{n}_{u}=0}^{\infty }\frac{\mathrm{exp}\left[-{\lambda }_{u}dt{M}_{{Y}_{D,u}^{*}}\left({h}_{D,u}\right)\right]\cdot {\left[{\lambda }_{u}dt\cdot {M}_{{Y}_{D,u}^{*}}\left({h}_{D,u}\right)\right]}^{{n}_{u}}}{{n}_{u}!}\cdot {\prod }_{n=0}^{{n}_{u}}\frac{\mathrm{exp}\left({h}_{D,u}\cdot {Y}_{D,u,n}^{*}\right)\cdot {f}_{{Y}_{D,u}^{*}}^{\mathbb{P}}\left(x\right)}{{M}_{{Y}_{D,u}^{*}}\left({h}_{D,u}\right)} \cdot {\sum }_{{n}_{d}=0}^{\infty }\frac{\mathrm{exp}\left[-{\lambda }_{d}dt{M}_{{Y}_{D,d}^{*}}\left({h}_{D,d}\right)\right]\cdot {\left[{\lambda }_{d}dt\cdot {M}_{{Y}_{D,d}^{*}}\left({h}_{D,d}\right)\right]}^{{n}_{d}}}{{n}_{d}!}\cdot {\prod }_{n=0}^{{n}_{d}}\frac{\mathrm{exp}\left({h}_{D,d}\cdot {Y}_{D,d,n}^{*}\right)\cdot {f}_{{Y}_{D,d}^{*}}^{\mathbb{P}}\left(x\right)}{{M}_{{Y}_{D,d}^{*}}\left({h}_{D,d}\right)} ,$$

where ${f}_{{Y}_{D,u}^{*}}^{\mathbb{P}}\left(x\right)=Normal\left(x;{\theta }_{D,u},{\nu }_{D,u}^{2}\right)$ and ${f}_{{Y}_{D,d}^{*}}^{\mathbb{P}}\left(x\right)=Normal\left(x;{\theta }_{D,d},{\nu }_{D,d}^{2}\right)$. Therefore, under the ${\mathbb{Q}}$ measure, the jump size of upside jump and downside jump are

$${f}_{{Y}_{D,u}^{*}}^{\mathbb{Q}}\left(x\right)=\frac{\mathrm{exp}\left({h}_{D,u}\cdot {Y}_{D,u,n}^{*}\right)\cdot {f}_{{Y}_{D,u}^{*}}^{\mathbb{P}}\left(x\right)}{{M}_{{Y}_{D,u}^{*}}\left({h}_{D,u}\right)}=\frac{1}{\sqrt{2\pi {\nu }_{D,u}^{2}}}\mathrm{exp}\left[-\frac{{\left(x-{\theta }_{D,u}-{h}_{D,u}{\nu }_{D,u}^{2}\right)}^{2}}{2{\nu }_{D,u}^{2}}\right]=Normal\left(x;{\theta }_{D,u}+{h}_{D,u}{\nu }_{D,u}^{2},{\nu }_{D,u}^{2}\right),{f}_{{Y}_{D,d}^{*}}^{\mathbb{Q}}\left(x\right)=\frac{\mathrm{exp}\left({h}_{D,d}\cdot {Y}_{D,d,n}^{*}\right)\cdot {f}_{{Y}_{D,d}^{*}}^{\mathbb{P}}\left(x\right)}{{M}_{{Y}_{D,d}^{*}}\left({h}_{D,d}\right)}=\frac{1}{\sqrt{2\pi {\nu }_{D,d}^{2}}}\mathrm{exp}\left[-\frac{{\left(x-{\theta }_{D,d}-{h}_{D,d}{\nu }_{D,d}^{2}\right)}^{2}}{2{\nu }_{D,d}^{2}}\right]=Normal\left(x;{\theta }_{D,d}+{h}_{D,d}{\nu }_{D,d}^{2},{\nu }_{D,d}^{2}\right).$$

Under the ${\mathbb{Q}}$ measure, the jump intensity of upside jump and downside jump are

$$d{N}_{u}\left(t\right)\sim Poisson\left({\lambda }_{u}dt{M}_{{Y}_{D,u}^{*}}\left({h}_{D,u}\right)\right)=Poisson\left({\lambda }_{u}dt\mathrm{exp}\left({h}_{D,u}{\theta }_{D,u}+\frac{1}{2}{h}_{D,u}^{2}{\nu }_{D,u}^{2}\right)\right)d{N}_{d}\left(t\right)\sim Poisson\left({\lambda }_{d}dt{M}_{{Y}_{D,d}^{*}}\left({h}_{D,d}\right)\right)=Poisson\left({\lambda }_{d}dt\mathrm{exp}\left({h}_{D,d}{\theta }_{D,d}+\frac{1}{2}{h}_{D,d}^{2}{\nu }_{D,d}^{2}\right)\right)$$

Define the domestic money market account is ${B}_{D}\left(t\right)=\mathrm{exp}\left[{\int }_{0}^{t}{r}_{D}\left(u\right)du\right]$. Therefore, the martingale condition is

69 $${\mathbb{E}}^{{\mathbb{Q}}_{D}}\left\{\left.\frac{{P}_{D}\left(t,T\right)}{{B}_{D}\left(t\right)}\right|{\mathcal{F}}_{0}\right\}=\frac{{P}_{D}\left(0,T\right)}{{B}_{D}\left(0\right)}$$

70 $$\iff \left[-{\alpha }_{D}^{*}\left(t,T\right)+\frac{1}{2}{{\sigma }_{D}^{*}}^{2}\left(t,T\right)-{h}_{1,D}{{\sigma }_{D}^{*}}^{2}\left(t,T\right)\right]-{\lambda }_{u}\left[\mathrm{exp}\left({h}_{D,u}{\theta }_{D,u}+\frac{1}{2}{h}_{D,u}^{2}{\nu }_{D,u}^{2}\right)-1\right]-{\lambda }_{d}\left[\mathrm{exp}\left({h}_{D,d}{\theta }_{D,d}+\frac{1}{2}{h}_{D,d}^{2}{\nu }_{D,d}^{2}\right)-1\right]=0$$

### Dynamic process of foreign interest rate

The foreign bond price in units of the foreign currency, can be written as follows.

71 $${P}_{F}\left(t,T\right)=\mathrm{exp}\left[-{\int }_{t}^{T}{f}_{F}\left(t,{t}_{\alpha }\right)d{t}_{\alpha }\right].$$

Similar to the domestic interest rate, the dynamic process of foreign zero-coupon bond can be written as follows.

72 $$\frac{d{P}_{F}\left(t,T\right)}{{P}_{F}\left(t,T\right)}=\left[{r}_{F}\left(t\right)-{\alpha }_{F}^{*}\left(t,T\right)+\frac{1}{2}{{\sigma }_{F}^{*}}^{2}\left(t,T\right)\right]dt-{\sigma }_{F}^{*}\left(t,T\right)d{W}_{D}^{\mathbb{P}}\left(t\right)+\left\{\mathrm{exp}\left[-{Y}_{F,u}^{*}\left(t,T\right)\right]-1\right\}d{N}_{u}\left(t\right)+\left\{\mathrm{exp}\left[-{Y}_{F,d}^{*}\left(t,T\right)\right]-1\right\}d{N}_{d}\left(t\right)$$

where ${r}_{F}\left(t\right)={f}_{F} (t,t)$ is foreign instantaneous spot rate, ${\alpha }_{F}^{*}\left(t,T\right)={\int }_{t}^{T}{\alpha }_{F}\left(t,{t}_{\alpha }\right)d{t}_{\alpha }$ is the integration of drift term, ${\sigma }_{F}^{*}\left(t,T\right)={\int }_{t}^{T}{\sigma }_{F}\left(t,{t}_{\alpha }\right)d{t}_{\alpha }$ is the integration of diffusion term, ${Y}_{F,u}^{*}\left(t,T\right)={\int }_{t}^{T}{Y}_{F,u}\left(t,{t}_{\alpha }\right)d{t}_{\alpha }$ is the integration of upside jump amplitude, and ${Y}_{F,d}^{*}\left(t,T\right)={\int }_{t}^{T}{Y}_{F,d}\left(t,{t}_{\alpha }\right)d{t}_{\alpha }$ is the integration of downside jump amplitude.

The jump size of domestic upside jump and downside jump are assumed to be random numbers, which follow the normal distribution, ${Y}_{F,u}^{*}\left(t,T\right)\sim Normal\left({\theta }_{F,u},{\nu }_{F,u}^{2}\right)$ and ${Y}_{F,d}^{*}\left(t,T\right)\sim Normal\left({\theta }_{F,d},{\nu }_{F,d}^{2}\right)$. Therefore, the dynamic process of domestic zero-coupon bond will be

73 $$\frac{d{P}_{F}\left(t,T\right)}{{P}_{F}\left(t,T\right)}=\left[{r}_{F}\left(t\right)-{\alpha }_{F}^{*}\left(t,T\right)+\frac{1}{2}{{\sigma }_{F}^{*}}^{2}\left(t,T\right)\right]dt-{\sigma }_{F}^{*}\left(t,T\right)d{W}_{F}^{\mathbb{P}}\left(t\right)+\left[\mathrm{exp}\left(-{Y}_{F,u}^{*}\right)-1\right]d{N}_{u}\left(t\right)+\left[\mathrm{exp}\left(-{Y}_{F,d}^{*}\right)-1\right]d{N}_{d}\left(t\right),$$

74 $${P}_{F}\left(t,T\right)={P}_{F}\left(0,T\right)\cdot \mathrm{exp}\left\{{\int }_{0}^{t}\left[{r}_{F}\left({t}_{\alpha }\right)-{\alpha }_{F}^{*}\left({t}_{\alpha },T\right)\right]d{t}_{\alpha }-{\int }_{0}^{t}{\sigma }_{F}^{*}\left({t}_{\alpha },T\right)d{W}_{F}^{\mathbb{P}}\left({t}_{\alpha }\right)\right\}\cdot \mathrm{exp}\left[-{Y}_{F,u}^{*}d{N}_{u}\left(t\right)\right]\cdot \mathrm{exp}\left[-{Y}_{F,d}^{*}d{N}_{d}\left(t\right)\right],$$

75 $$d\mathrm{ln}{P}_{F}\left(t,T\right)=\left[{r}_{F}\left(t\right)-{\alpha }_{F}^{*}\left(t,T\right)\right]dt-{\sigma }_{F}^{*}\left(t,T\right)d{W}_{F}^{\mathbb{P}}\left(t\right)-{Y}_{F,u}^{*}d{N}_{u}\left(t\right)-{Y}_{F,d}^{*}d{N}_{d}\left(t\right).$$

Similarly, the MGF of the compound Poisson process ${J}_{F,u}={Y}_{F,u}^{*}d{N}_{u}\left(t\right)$ with parameter ${h}_{F,u}$ is

76 $${M}_{{J}_{F,u}}\left({h}_{F,u}\right)=\mathrm{exp}\left\{{\lambda }_{u}dt\left[{M}_{{Y}_{F,u}^{*}}\left({h}_{F,u}\right)-1\right]\right\},$$

where ${M}_{{Y}_{F,u}^{*}}\left({h}_{F,u}\right)=\mathrm{exp}\left({h}_{F,u}{\theta }_{F,u}+\frac{1}{2}{h}_{F,u}^{2}{\nu }_{F,u}^{2}\right)$ is the MGF of the distribution $Normal\left({\theta }_{F,u},{\nu }_{F,u}^{2}\right)$.

The MGF of the compound Poisson process ${J}_{F,d}={Y}_{F,d}^{*}d{N}_{d}\left(t\right)$ with parameter ${h}_{F,d}$ is

77 $${M}_{{J}_{F,d}}\left({h}_{F,d}\right)=\mathrm{exp}\left\{{\lambda }_{d}dt\left[{M}_{{Y}_{F,d}^{*}}\left({h}_{F,d}\right)-1\right]\right\},$$

where ${M}_{{Y}_{F,d}^{*}}\left({h}_{F,d}\right)=\mathrm{exp}\left({h}_{F,d}{\theta }_{F,d}+\frac{1}{2}{h}_{F,d}^{2}{\nu }_{F,d}^{2}\right)$ is the MGF of the distribution $Normal\left({\theta }_{F,d},{\nu }_{F,d}^{2}\right)$.

Similar to the domestic interest rate, the Radon-Nikodým derivative of the Esscher transform is given by:

78 $${\xi }^{{h}_{F}}\left(t\right)=\left.\frac{d{\mathbb{Q}}^{{h}_{F}}}{d{\mathbb{P}}}\right|\mathcal{F}\left(t\right)$$

79 $$=\frac{\mathrm{exp}\left\{{h}_{1,F}{\sigma }_{F}^{*}\left(t,T\right)d{W}_{F}^{\mathbb{P}}\left(t\right)\right\}}{{\mathbb{E}}^{\mathbb{P}}\left\{\mathrm{exp}\left[{h}_{1,F}{\sigma }_{F}^{*}\left(t,T\right)d{W}_{F}^{\mathbb{P}}\left(t\right)\right]\right\}}\cdot \frac{\mathrm{exp}\left\{{h}_{F,u}\cdot {Y}_{F,u}^{*}d{N}_{u}\left(t\right)\right\}}{{\mathbb{E}}^{\mathbb{P}}\left\{\mathrm{exp}\left[{h}_{F,u}\cdot {Y}_{F,u}^{*}d{N}_{u}\left(t\right)\right]\right\}}\cdot \frac{\mathrm{exp}\left\{{h}_{F,d}\cdot {Y}_{F,d}^{*}d{N}_{d}\left(t\right)\right\}}{{\mathbb{E}}^{\mathbb{P}}\left\{\mathrm{exp}\left[{h}_{F,d}\cdot {Y}_{F,d}^{*}d{N}_{d}\left(t\right)\right]\right\}}$$

80 $$=\mathrm{exp}\left[{h}_{1,F}{\sigma }_{F}^{*}\left(t,T\right)d{W}_{F}^{\mathbb{P}}\left(t\right)-\frac{1}{2}{h}_{1,F}^{2}{{\sigma }_{F}^{*}}^{2}\left(t,T\right)dt\right]\cdot \mathrm{exp}\left\{{h}_{F,u}\cdot {Y}_{F,u}^{*}d{N}_{u}\left(t\right)-{\lambda }_{u}dt\left[\mathrm{exp}\left({h}_{F,u}{\theta }_{F,u}+\frac{1}{2}{h}_{F,u}^{2}{\nu }_{F,u}^{2}\right)-1\right]\right\}\cdot \mathrm{exp}\left\{{h}_{F,d}\cdot {Y}_{F,d}^{*}d{N}_{d}\left(t\right)-{\lambda }_{d}dt\left[\mathrm{exp}\left({h}_{F,d}{\theta }_{F,d}+\frac{1}{2}{h}_{F,d}^{2}{\nu }_{F,d}^{2}\right)-1\right]\right\}.$$

Let ${\Lambda }_{t}^{{h}_{F}}=\left.\frac{d{\mathbb{Q}}^{{h}_{F}}}{d{\mathbb{P}}}\right|\mathcal{F}\left(t\right)={\Lambda }_{t}^{{C}_{F}}\times {\Lambda }_{t}^{{J}_{F}}$. For the jump part, it has

81 $${\Lambda }_{t}^{{J}_{F}}=\left.\frac{d{\mathbb{Q}}^{{J}_{F}}}{d{\mathbb{P}}^{{J}_{F}}}\right|\mathcal{F}\left(t\right)=\frac{\mathrm{exp}\left\{{h}_{F,u}\cdot {Y}_{F,u}^{*}d{N}_{u}\left(t\right)\right\}}{{\mathbb{E}}^{\mathbb{P}}\left\{\mathrm{exp}\left[{h}_{F,u}\cdot {Y}_{F,u}^{*}d{N}_{u}\left(t\right)\right]\right\}}\cdot \frac{\mathrm{exp}\left\{{h}_{F,d}\cdot {Y}_{F,d}^{*}d{N}_{d}\left(t\right)\right\}}{{\mathbb{E}}^{\mathbb{P}}\left\{\mathrm{exp}\left[{h}_{F,d}\cdot {Y}_{F,d}^{*}d{N}_{d}\left(t\right)\right]\right\}},$$

82 $$d{\mathbb{Q}}^{{J}_{F}}={\sum }_{{n}_{u}=0}^{\infty }\frac{\mathrm{exp}\left[-{\lambda }_{u}dt{M}_{{Y}_{F,u}^{*}}\left({h}_{F,u}\right)\right]\cdot {\left[{\lambda }_{u}dt\cdot {M}_{{Y}_{F,u}^{*}}\left({h}_{F,u}\right)\right]}^{{n}_{u}}}{{n}_{u}!}\cdot {\prod }_{n=0}^{{n}_{u}}\frac{\mathrm{exp}\left({h}_{F,u}\cdot {Y}_{F,u,n}^{*}\right)\cdot {f}_{{Y}_{F,u}^{*}}^{\mathbb{P}}\left(x\right)}{{M}_{{Y}_{F,u}^{*}}\left({h}_{F,u}\right)} \cdot {\sum }_{{n}_{d}=0}^{\infty }\frac{\mathrm{exp}\left[-{\lambda }_{d}dt{M}_{{Y}_{F,d}^{*}}\left({h}_{F,d}\right)\right]\cdot {\left[{\lambda }_{d}dt\cdot {M}_{{Y}_{F,d}^{*}}\left({h}_{F,d}\right)\right]}^{{n}_{d}}}{{n}_{d}!}\cdot {\prod }_{n=0}^{{n}_{d}}\frac{\mathrm{exp}\left({h}_{F,d}\cdot {Y}_{F,d,n}^{*}\right)\cdot {f}_{{Y}_{F,d}^{*}}^{\mathbb{P}}\left(x\right)}{{M}_{{Y}_{F,d}^{*}}\left({h}_{F,d}\right)} ,$$

where $${f}_{{Y}_{F,u}^{*}}^{\mathbb{P}}\left(x\right)=Normal\left(x;{\theta }_{F,u},{\nu }_{F,u}^{2}\right)$$ and $${f}_{{Y}_{F,d}^{*}}^{\mathbb{P}}\left(x\right)=Normal\left(x;{\theta }_{F,d},{\nu }_{F,d}^{2}\right)$$.

Therefore, under the ${\mathbb{Q}}$ measure, the jump size of upside jump and downside jump are

$${f}_{{Y}_{F,u}^{*}}^{\mathbb{Q}}\left(x\right)=\frac{\mathrm{exp}\left({h}_{F,u}\cdot {Y}_{F,u,n}^{*}\right)\cdot {f}_{{Y}_{F,u}^{*}}^{\mathbb{P}}\left(x\right)}{{M}_{{Y}_{F,u}^{*}}\left({h}_{F,u}\right)}=Normal\left(x;{\theta }_{F,u}+{h}_{F,u}{\nu }_{F,u}^{2},{\nu }_{F,u}^{2}\right),{f}_{{Y}_{F,d}^{*}}^{\mathbb{Q}}\left(x\right)=\frac{\mathrm{exp}\left({h}_{F,d}\cdot {Y}_{F,d,n}^{*}\right)\cdot {f}_{{Y}_{F,d}^{*}}^{\mathbb{P}}\left(x\right)}{{M}_{{Y}_{F,d}^{*}}\left({h}_{F,d}\right)}=Normal\left(x;{\theta }_{F,d}+{h}_{F,d}{\nu }_{F,d}^{2},{\nu }_{F,d}^{2}\right).$$

Under the ${\mathbb{Q}}$ measure, the jump intensity of upside jump and downside jump are as follows:

$$d{N}_{u}\left(t\right)\sim Poisson\left({\lambda }_{u}dt{M}_{{Y}_{F,u}^{*}}\left({h}_{F,u}\right)\right)=Poisson\left({\lambda }_{u}dt\mathrm{exp}\left({h}_{F,u}{\theta }_{F,u}+\frac{1}{2}{h}_{F,u}^{2}{\nu }_{F,u}^{2}\right)\right),d{N}_{d}\left(t\right)\sim Poisson\left({\lambda }_{d}dt{M}_{{Y}_{F,d}^{*}}\left({h}_{F,d}\right)\right)=Poisson\left({\lambda }_{d}dt\mathrm{exp}\left({h}_{F,d}{\theta }_{F,d}+\frac{1}{2}{h}_{F,d}^{2}{\nu }_{F,d}^{2}\right)\right).$$

Since the domestic money market account is ${B}_{D}\left(t\right)=\mathrm{exp}\left[{\int }_{0}^{t}{r}_{D}\left(u\right)du\right]$, the martingale condition is

83 $${\mathbb{E}}^{{\mathbb{Q}}_{D}}\left\{\left.\frac{{P}_{F}\left(t,T\right)}{{B}_{D}\left(t\right)}\right|{\mathcal{F}}_{0}\right\}=\frac{{P}_{F}\left(0,T\right)}{{B}_{D}\left(0\right)}$$

84 $$\iff \left\{\left[{r}_{F}\left(t\right)-{r}_{D}\left(t\right)-{\alpha }_{F}^{*}\left(u,T\right)\right]+\frac{1}{2}{{\sigma }_{F}^{*}}^{2}\left(t,T\right)-{h}_{1,F}{{\sigma }_{F}^{*}}^{2}\left(t,T\right)\right\}-{\lambda }_{u}\left[\mathrm{exp}\left({h}_{F,u}{\theta }_{F,u}+\frac{1}{2}{h}_{F,u}^{2}{\nu }_{F,u}^{2}\right)-1\right]-{\lambda }_{d}\left[\mathrm{exp}\left({h}_{F,d}{\theta }_{F,d}+\frac{1}{2}{h}_{F,d}^{2}{\nu }_{F,d}^{2}\right)-1\right]=0.$$

See Tables 1, 2, 3, 4, 5, 6, 7, 8.

**Table 1:** Descriptive statistics

	US	EU	UK	JP
*Panel (a): 1-Month LIBOR*				
Mean (%)	− 0.0002	− 0.0006	− 0.0008	0.0000
Std. (%)	0.0257	0.0157	0.0260	0.0100
Skewness	− 2.1912	12.8803	− 19.8004	10.7682
Kurtosis	119.9268	683.7840	1095.9627	375.7140
*Panel (b): 3-Month LIBOR*				
Mean (%)	− 0.0002	− 0.0006	− 0.0008	0.0000
Std. (%)	0.0221	0.0103	0.0215	0.0050
Skewness	− 4.6322	− 1.6300	− 28.1122	1.3630
Kurtosis	103.9143	32.0530	1364.8970	41.9883
*Panel (c): 6-Month LIBOR*				
Mean (%)	− 0.0002	− 0.0006	− 0.0008	0.0000
Std. (%)	0.0238	0.0110	0.0218	0.0047
Skewness	− 2.4002	− 0.4917	− 27.2462	1.8057
Kurtosis	56.7922	34.9124	1334.2701	44.7464
*Panel (d): 12-Month LIBOR*				
Mean (%)	− 0.0002	− 0.0006	− 0.0009	0.0000
Std. (%)	0.0308	0.0146	0.0245	0.0050
Skewness	− 0.3537	1.7316	− 19.4862	0.8376
Kurtosis	25.0563	46.1529	873.4830	69.2170
*Panel (e): Exchange Rate*		EUR/USD	GBP/USD	JPY/USD
Mean (%)		− 0.0011	0.0044	− 0.0017
Std. (%)		0.5778	6.6601	0.6104
Skewness		− 0.1054	− 0.0028	− 0.3157
Kurtosis		3.2436	20.2684	5.1661

This table shows the descriptive statistics of the first-order difference of daily 1-, 3-, 6-, and 12-month LIBORs and the log-return of the daily spot exchange rate. The sample period starts from October 1, 2003 to December 31, 2020. The number of observable data for each type is 4502

**Table 2:** Parametric estimation results for EUR/USD

	1-Month				3-Month				6-Month				12-Month			
	CB	CB–ISJ	CB–CSJ	CB–CAJ	CB	CB–ISJ	CB–CSJ	CB–CAJ	CB	CB–ISJ	CB–CSJ	CB–CAJ	CB	CB–ISJ	CB–CSJ	CB–CAJ
*Panel (a): Continuous Part*																
${\mu }_{D}$	− 0.000921	0.002124	− 0.000727	− 0.000775	− 0.000135	0.003679	0.001183	− 0.000083	0.000055	0.006230	0.000152	− 0.001298	0.005301	0.003183	− 0.000608	− 0.000815
	(0.0006)	(0.0008)	(0.0004)	(0.0003)	(0.0003)	(0.0013)	(0.0007)	(0.0004)	(0.0000)	(0.0014)	(0.0010)	(0.0003)	(0.0009)	(0.0010)	(0.0006)	(0.0004)
${\mu }_{F}$	− 0.001714	− 0.004993	− 0.004543	− 0.004944	− 0.003632	− 0.005032	− 0.006675	− 0.006107	− 0.006200	− 0.005742	− 0.006905	− 0.006749	− 0.006774	− 0.008479	− 0.011742	− 0.011111
	(0.0005)	(0.0014)	(0.0010)	(0.0009)	(0.0007)	(0.0013)	(0.0008)	(0.0008)	(0.0009)	(0.0013)	(0.0010)	(0.0009)	(0.0010)	(0.0013)	(0.0011)	(0.0011)
${\mu }_{X}$	0.009624	0.066988	0.043084	0.036623	0.011472	0.059233	0.035017	0.032122	0.008565	0.058256	0.030661	0.032705	0.017788	0.033536	0.007475	0.010433
	(0.0017)	(0.0055)	(0.0041)	(0.0032)	(0.0017)	(0.0067)	(0.0056)	(0.0053)	(0.0010)	(0.0082)	(0.0058)	(0.0052)	(0.0020)	(0.0074)	(0.0059)	(0.0055)
${\sigma }_{D}$	0.000217	0.037925	0.013870	0.006984	0.000383	0.051679	0.020141	0.008219	0.000458	0.060723	0.019860	0.001230	0.051970	0.048572	0.016423	0.005987
	(0.0001)	(0.0052)	(0.0032)	(0.0022)	(0.0002)	(0.0069)	(0.0043)	(0.0025)	(0.0002)	(0.0072)	(0.0044)	(0.0005)	(0.0088)	(0.0060)	(0.0034)	(0.0020)
${\sigma }_{F}$	0.000233	0.012128	0.009276	0.004120	0.000799	0.017671	0.001741	0.000439	0.001475	0.015408	0.003728	0.000174	0.026883	0.014897	0.002050	0.000528
	(0.0001)	(0.0040)	(0.0034)	(0.0024)	(0.0002)	(0.0043)	(0.0008)	(0.0004)	(0.0002)	(0.0042)	(0.0017)	(0.0000)	(0.0042)	(0.0036)	(0.0006)	(0.0002)
${\sigma }_{X}$	0.005820	0.214761	0.141936	0.118903	0.005567	0.224697	0.083906	0.061079	0.006091	0.172552	0.084800	0.046469	0.057822	0.154126	0.065259	0.033368
	(0.0001)	(0.0119)	(0.0099)	(0.0080)	(0.0002)	(0.0128)	(0.0078)	(0.0065)	(0.0002)	(0.0136)	(0.0088)	(0.0050)	(0.0087)	(0.0124)	(0.0068)	(0.0037)
${\rho }_{DF}$	0.042803	− 0.084923	0.176238	0.155151	0.045667	− 0.117856	0.082364	0.055962	0.030065	− 0.054998	0.064719	0.097790	0.023745	0.013141	0.059020	0.094724
	(0.0220)	(0.0437)	(0.0364)	(0.0362)	(0.0222)	(0.0403)	(0.0332)	(0.0327)	(0.0227)	(0.0408)	(0.0338)	(0.0317)	(0.0204)	(0.0400)	(0.0324)	(0.0312)
${\rho }_{DX}$	− 0.076668	0.234528	0.347905	0.369591	− 0.119865	0.272173	0.277339	0.245178	− 0.114798	0.247017	0.318158	0.331476	− 0.274496	0.265379	0.281603	0.221663
	(0.0175)	(0.0307)	(0.0249)	(0.0248)	(0.0198)	(0.0293)	(0.0283)	(0.0287)	(0.0202)	(0.0303)	(0.0289)	(0.0263)	(0.0261)	(0.0299)	(0.0298)	(0.0292)
${\rho }_{FX}$	0.046973	− 0.213778	− 0.403137	− 0.403660	− 0.054756	− 0.159177	− 0.328638	− 0.317723	0.007442	− 0.143966	− 0.374716	− 0.319880	0.073694	− 0.134918	− 0.253538	− 0.264014
	(0.0161)	(0.0293)	(0.0241)	(0.0232)	(0.0229)	(0.0307)	(0.0277)	(0.0275)	(0.0266)	(0.0322)	(0.0293)	(0.0286)	(0.0272)	(0.0308)	(0.0316)	(0.0292)
${h}_{1,D}$	0.036477	− 0.138793	− 0.080802	− 0.076683	0.036340	− 0.125473	− 0.042385	− 0.022603	0.020723	− 0.097229	− 0.056506	− 0.011766	0.077461	− 0.062419	− 0.042461	− 0.015872
	(0.0061)	(0.0113)	(0.0124)	(0.0124)	(0.0105)	(0.0104)	(0.0130)	(0.0136)	(0.0124)	(0.0116)	(0.0138)	(0.0143)	(0.0141)	(0.0122)	(0.0126)	(0.0127)
${h}_{1,F}$	0.018294	− 0.122076	− 0.022409	− 0.017339	0.001183	− 0.053533	− 0.021341	− 0.016073	− 0.035795	− 0.053031	− 0.002279	0.011101	− 0.052295	− 0.025456	0.033677	0.026347
	(0.0057)	(0.0121)	(0.0129)	(0.0126)	(0.0113)	(0.0132)	(0.0124)	(0.0129)	(0.0117)	(0.0140)	(0.0136)	(0.0140)	(0.0146)	(0.0139)	(0.0133)	(0.0132)
${h}_{1,X}$	0.025029	0.005214	0.011537	0.015560	0.037336	0.003009	0.004603	− 0.003558	0.041968	− 0.004925	− 0.007003	− 0.006280	0.071737	− 0.000818	− 0.023318	− 0.025890
	(0.0052)	(0.0114)	(0.0109)	(0.0109)	(0.0050)	(0.0122)	(0.0119)	(0.0120)	(0.0063)	(0.0126)	(0.0128)	(0.0129)	(0.0115)	(0.0126)	(0.0129)	(0.0128)
*Panel (b): Jump Part*																
${\theta }_{D,u}\left({\theta }_{D}\right)$		− 0.100688	− 0.079268	− 0.041040		− 0.095572	− 0.055884	− 0.024121		− 0.076280	− 0.091150	− 0.036647		− 0.102691	− 0.086787	− 0.041951
		(0.0106)	(0.0088)	(0.0060)		(0.0111)	(0.0085)	(0.0049)		(0.0102)	(0.0084)	(0.0060)		(0.0120)	(0.0097)	(0.0097)
${\theta }_{F,u}\left({\theta }_{F}\right)$		− 0.107118	− 0.090019	− 0.048872		− 0.096301	− 0.121035	− 0.062113		− 0.092275	− 0.090156	− 0.052071		− 0.106841	− 0.133530	− 0.053116
		(0.0122)	(0.0093)	(0.0061)		(0.0108)	(0.0089)	(0.0088)		(0.0094)	(0.0066)	(0.0063)		(0.0113)	(0.0106)	(0.0091)
${\theta }_{X,u}\left({\theta }_{X}\right)$		0.010272	0.008520	0.057719		− 0.011493	− 0.007382	0.093479		0.007724	− 0.020850	0.096152		− 0.043176	− 0.113097	0.077248
		(0.0165)	(0.0125)	(0.0084)		(0.0221)	(0.0179)	(0.0113)		(0.0214)	(0.0208)	(0.0130)		(0.0228)	(0.0193)	(0.0127)
${\theta }_{T,u}\left({\theta }_{T}\right)$		− 0.127245	− 0.047148	− 0.021196		− 0.093518	− 0.027779	− 0.013091		− 0.073321	− 0.019149	− 0.009557		− 0.064898	− 0.008095	− 0.004291
		(0.0089)	(0.0052)	(0.0028)		(0.0066)	(0.0037)	(0.0026)		(0.0041)	(0.0023)	(0.0016)		(0.0045)	(0.0012)	(0.0010)
${\theta }_{D,d}$				− 0.037718				− 0.040424				− 0.036153				− 0.056477
				(0.0074)				(0.0065)				(0.0056)				(0.0093)
${\theta }_{F,d}$				− 0.031286				− 0.053169				− 0.036759				− 0.083891
				(0.0072)				(0.0092)				(0.0070)				(0.0109)
${\theta }_{X,d}$				− 0.065160				− 0.115084				− 0.113685				− 0.171123
				(0.0083)				(0.0137)				(0.0120)				(0.0154)
${\theta }_{T,d}$				− 0.026578				− 0.021690				− 0.016061				− 0.010816
				(0.0041)				(0.0031)				(0.0017)				(0.0014)
${\nu }_{D,u}\left({\nu }_{D}\right)$		0.335811	0.314102	0.204786		0.337551	0.291981	0.169110		0.292838	0.356751	0.244913		0.340109	0.353738	0.200328
		(0.0192)	(0.0157)	(0.0159)		(0.0200)	(0.0140)	(0.0138)		(0.0184)	(0.0150)	(0.0166)		(0.0203)	(0.0167)	(0.0183)
${\nu }_{F,u}\left({\nu }_{F}\right)$		0.259790	0.268501	0.202711		0.280429	0.323837	0.196639		0.255154	0.277162	0.213445		0.279514	0.326765	0.169572
		(0.0167)	(0.0130)	(0.0148)		(0.0167)	(0.0144)	(0.0162)		(0.0161)	(0.0110)	(0.0142)		(0.0164)	(0.0128)	(0.0150)
${\nu }_{X,u}\left({\nu }_{X}\right)$		0.348739	0.286168	0.185695		0.374289	0.354920	0.192929		0.379360	0.328660	0.220082		0.328770	0.337435	0.167851
		(0.0145)	(0.0111)	(0.0130)		(0.0156)	(0.0140)	(0.0151)		(0.0173)	(0.0134)	(0.0153)		(0.0153)	(0.0140)	(0.0146)
${\nu }_{T,u}$		0.385073	0.214064	0.117978		0.348324	0.157044	0.077925		0.327029	0.153397	0.071872		0.314040	0.112876	0.046910
		(0.0115)	(0.0094)	(0.0094)		(0.0100)	(0.0076)	(0.0066)		(0.0087)	(0.0088)	(0.0071)		(0.0097)	(0.0065)	(0.0051)
${\nu }_{D,d}$				0.178138				0.202540				0.245294				0.261442
				(0.0158)				(0.0172)				(0.0177)				(0.0179)
${\nu }_{F,d}$				0.145932				0.189175				0.196922				0.265231
				(0.0136)				(0.0161)				(0.0138)				(0.0159)
${\nu }_{X,d}$				0.148396				0.198658				0.204077				0.243379
				(0.0120)				(0.0158)				(0.0153)				(0.0158)
${\nu }_{T,d}$				0.122344				0.111278				0.113663				0.091415
				(0.0104)				(0.0094)				(0.0085)				(0.0070)
${\phi }_{DF,u}\left({\phi }_{DF}\right)$			0.056727	0.023693			− 0.010389	− 0.029232			0.070793	0.027077			0.092833	0.045615
			(0.0340)	(0.0266)			(0.0362)	(0.0286)			(0.0379)	(0.0299)			(0.0384)	(0.0254)
${\phi }_{DX,u}\left({\phi }_{DX}\right)$			− 0.021682	− 0.033227			0.017727	0.001234			0.033392	− 0.015567			0.047684	− 0.022359
			(0.0330)	(0.0242)			(0.0350)	(0.0285)			(0.0367)	(0.0311)			(0.0352)	(0.0258)
${\phi }_{FX,u}\left({\phi }_{FX}\right)$			− 0.021208	− 0.044786			− 0.037041	− 0.079530			0.009425	− 0.025997			0.085253	− 0.023325
			(0.0324)	(0.0257)			(0.0329)	(0.0256)			(0.0383)	(0.0297)			(0.0358)	(0.0260)
${\phi }_{DF,d}$				− 0.016848				0.065124				− 0.008434				0.042699
				(0.0250)				(0.0279)				(0.0309)				(0.0327)
${\phi }_{DX,d}$				− 0.004270				0.013855				0.041967				− 0.007523
				(0.0247)				(0.0276)				(0.0280)				(0.0309)
${\phi }_{FX,d}$				0.032736				0.043400				0.024660				0.063930
				(0.0208)				(0.0256)				(0.0289)				(0.0301)
${\lambda }_{u}\left(\lambda \right)$		0.280881	0.206799	0.106294		0.295578	0.220862	0.096217		0.299466	0.255834	0.100483		0.322897	0.285843	0.076891
		(0.0101)	(0.0054)	(0.0069)		(0.0090)	(0.0076)	(0.0072)		(0.0100)	(0.0093)	(0.0070)		(0.0114)	(0.0106)	(0.0073)
${\lambda }_{d}$				0.105518				0.109510				0.129113				0.169682
				(0.0084)				(0.0096)				(0.0104)				(0.0123)
${h}_{D,u}\left({h}_{D}\right)$		0.001281	− 0.062000	− 0.034035		− 0.005769	− 0.044923	− 0.023695		0.005560	− 0.038939	− 0.031663		0.011681	− 0.041468	− 0.025064
		(0.0092)	(0.0076)	(0.0059)		(0.0087)	(0.0076)	(0.0064)		(0.0087)	(0.0092)	(0.0077)		(0.0088)	(0.0085)	(0.0076)
${h}_{F,u}\left({h}_{F}\right)$		− 0.005464	0.028080	0.024562		0.016891	0.032475	0.047101		0.008289	− 0.003961	0.032792		− 0.026714	− 0.090545	− 0.003138
		(0.0113)	(0.0111)	(0.0086)		(0.0116)	(0.0115)	(0.0108)		(0.0110)	(0.0119)	(0.0093)		(0.0114)	(0.0109)	(0.0087)
${h}_{X,u}\left({h}_{X}\right)$		0.006704	− 0.006453	− 0.011616		0.013229	0.012234	0.017305		0.015738	0.050872	0.048587		0.013443	0.026627	0.012721
		(0.0020)	(0.0039)	(0.0036)		(0.0024)	(0.0074)	(0.0058)		(0.0037)	(0.0094)	(0.0070)		(0.0034)	(0.0092)	(0.0063)
${h}_{D,d}$				− 0.022063				− 0.014611				0.014805				− 0.008055
				(0.0060)				(0.0073)				(0.0081)				(0.0077)
${h}_{F,d}$				0.003424				− 0.003981				− 0.019386				− 0.057708
				(0.0082)				(0.0095)				(0.0100)				(0.0102)
${h}_{X,d}$				0.004931				0.005036				0.027671				0.033705
				(0.0029)				(0.0054)				(0.0061)				(0.0072)
*Panel (c): Log-* *Likelihood*																
LL	2600.65	2784.55	3061.16	3123.21	2477.30	2518.48	3024.49	3118.30	2226.84	2385.18	2751.96	2903.53	2028.43	2278.30	2572.47	2675.10
LRT		367.81	553.22	124.10		82.37	1012.01	187.64		316.69	733.56	303.14		499.74	588.35	205.26

The results are calibrated from the sample shown in Table 1. This table lists the average and standard error of the mean of parametric estimation results for EUR/USD. The results are divided into the continuous part (Panel (a)) and the jump part (Panel (b)). For the jump part’s parameters in CB–ISJ and CB–CSJ models, the notations are shown in the brackets. For example, the mean of jump amplitude for the domestic interest rate in CB–ISJ and CB–CSJ models is ${\uptheta }_{\mathrm{D}}$ in the bracket

**Table 3:** Parametric estimation results for GBP/USD

	1-Month				3-Month				6-Month				12-Month			
	CB	CB–ISJ	CB–CSJ	CB–CAJ	CB	CB–ISJ	CB–CSJ	CB–CAJ	CB	CB–ISJ	CB–CSJ	CB–CAJ	CB	CB–ISJ	CB–CSJ	CB–CAJ
*Panel (a): Continuous Part*																
${\mu }_{D}$	0.000023	0.001735	− 0.000021	− 0.001128	− 0.000032	0.001538	− 0.000243	− 0.001272	0.000011	0.004428	− 0.000516	− 0.002100	0.004191	0.004534	− 0.000329	− 0.001700
	(0.0005)	(0.0010)	(0.0005)	(0.0003)	(0.0002)	(0.0013)	(0.0005)	(0.0004)	(0.0000)	(0.0012)	(0.0010)	(0.0004)	(0.0008)	(0.0015)	(0.0008)	(0.0003)
${\mu }_{F}$	− 0.001692	0.003022	− 0.001683	− 0.001395	− 0.002882	0.003913	− 0.002120	− 0.002537	− 0.005926	0.002633	− 0.003001	− 0.002559	− 0.007078	0.003409	− 0.006399	− 0.006178
	(0.0005)	(0.0011)	(0.0009)	(0.0009)	(0.0006)	(0.0012)	(0.0010)	(0.0009)	(0.0009)	(0.0014)	(0.0011)	(0.0010)	(0.0010)	(0.0015)	(0.0011)	(0.0010)
${\mu }_{X}$	0.008704	0.081062	0.036673	0.035420	0.009652	0.074079	0.037610	0.034647	0.008114	0.053493	0.032838	0.036395	0.014268	0.046245	0.016597	0.016590
	(0.0015)	(0.0065)	(0.0039)	(0.0036)	(0.0016)	(0.0072)	(0.0050)	(0.0048)	(0.0010)	(0.0063)	(0.0055)	(0.0050)	(0.0018)	(0.0081)	(0.0055)	(0.0051)
${\sigma }_{D}$	0.001164	0.039900	0.015375	0.004566	0.000317	0.047942	0.015131	0.003470	0.000461	0.060355	0.016918	0.002468	0.040012	0.054911	0.011378	0.001465
	(0.0010)	(0.0057)	(0.0031)	(0.0016)	(0.0002)	(0.0068)	(0.0031)	(0.0014)	(0.0002)	(0.0072)	(0.0042)	(0.0015)	(0.0078)	(0.0074)	(0.0034)	(0.0008)
${\sigma }_{F}$	0.001397	0.009865	0.002928	0.002541	0.000722	0.015150	0.002637	0.000405	0.001467	0.014908	0.003232	0.000142	0.021055	0.017009	0.001202	0.000231
	(0.0011)	(0.0037)	(0.0016)	(0.0015)	(0.0002)	(0.0040)	(0.0017)	(0.0003)	(0.0002)	(0.0043)	(0.0019)	(0.0000)	(0.0037)	(0.0045)	(0.0006)	(0.0000)
${\sigma }_{X}$	0.006481	0.251397	0.147092	0.132927	0.005605	0.249127	0.089347	0.074064	0.006120	0.204447	0.080414	0.059903	0.045350	0.163749	0.061711	0.048265
	(0.0005)	(0.0125)	(0.0092)	(0.0085)	(0.0001)	(0.0138)	(0.0067)	(0.0055)	(0.0002)	(0.0140)	(0.0069)	(0.0049)	(0.0077)	(0.0118)	(0.0054)	(0.0038)
${\rho }_{DF}$	0.117911	− 0.120885	0.168473	0.129840	0.057794	− 0.114276	0.052936	0.052605	0.036240	0.024893	0.063506	0.105965	0.044221	0.129292	0.179325	0.197009
	(0.0201)	(0.0452)	(0.0393)	(0.0403)	(0.0211)	(0.0416)	(0.0335)	(0.0326)	(0.0233)	(0.0405)	(0.0312)	(0.0300)	(0.0225)	(0.0378)	(0.0314)	(0.0289)
${\rho }_{DX}$	− 0.044294	0.239232	0.347351	0.338430	− 0.106519	0.217913	0.222447	0.209593	− 0.127541	0.202069	0.267598	0.244220	− 0.265086	0.233772	0.208486	0.189261
	(0.0181)	(0.0280)	(0.0237)	(0.0237)	(0.0197)	(0.0309)	(0.0285)	(0.0281)	(0.0198)	(0.0296)	(0.0280)	(0.0265)	(0.0251)	(0.0276)	(0.0283)	(0.0272)
${\rho }_{FX}$	0.036204	− 0.196205	− 0.348180	− 0.351234	− 0.018109	− 0.175236	− 0.250914	− 0.228900	0.019705	− 0.086937	− 0.270286	− 0.228922	0.111136	− 0.081577	− 0.208031	− 0.203972
	(0.0171)	(0.0293)	(0.0244)	(0.0237)	(0.0224)	(0.0286)	(0.0286)	(0.0278)	(0.0267)	(0.0296)	(0.0293)	(0.0281)	(0.0290)	(0.0289)	(0.0291)	(0.0278)
${h}_{1,D}$	0.058792	− 0.129914	− 0.093029	− 0.079904	0.031523	− 0.106096	− 0.041526	− 0.033477	0.032963	− 0.085967	− 0.039834	− 0.030325	0.066753	− 0.069152	− 0.041629	− 0.034419
	(0.0052)	(0.0107)	(0.0120)	(0.0120)	(0.0107)	(0.0117)	(0.0129)	(0.0132)	(0.0122)	(0.0119)	(0.0142)	(0.0145)	(0.0132)	(0.0117)	(0.0126)	(0.0128)
${h}_{1,F}$	0.014723	− 0.124973	− 0.042283	− 0.039590	0.000310	− 0.074357	− 0.017283	− 0.005943	− 0.021301	− 0.048779	0.015095	0.021575	− 0.048193	− 0.002592	0.022504	0.026745
	(0.0064)	(0.0121)	(0.0127)	(0.0128)	(0.0112)	(0.0125)	(0.0124)	(0.0123)	(0.0114)	(0.0133)	(0.0138)	(0.0141)	(0.0146)	(0.0131)	(0.0131)	(0.0134)
${h}_{1,X}$	0.025533	0.004156	0.004580	0.004003	0.037424	0.001015	0.017462	0.011677	0.044192	0.004052	0.005683	0.006648	0.071481	0.012248	− 0.014873	− 0.017806
	(0.0054)	(0.0114)	(0.0109)	(0.0108)	(0.0047)	(0.0123)	(0.0119)	(0.0121)	(0.0062)	(0.0135)	(0.0130)	(0.0126)	(0.0111)	(0.0135)	(0.0133)	(0.0132)
*Panel (b): Jump Part*																
${\theta }_{D,u}\left({\theta }_{D}\right)$		− 0.103941	− 0.078482	− 0.035271		− 0.127322	− 0.063849	− 0.036474		− 0.117015	− 0.105996	− 0.034718		− 0.105821	− 0.087733	− 0.032088
		(0.0116)	(0.0077)	(0.0055)		(0.0129)	(0.0092)	(0.0062)		(0.0141)	(0.0097)	(0.0069)		(0.0116)	(0.0082)	(0.0060)
${\theta }_{F,u}\left({\theta }_{F}\right)$		− 0.063852	− 0.079472	− 0.040376		− 0.074380	− 0.094229	− 0.043811		− 0.081537	− 0.085240	− 0.044208		− 0.073420	− 0.106547	− 0.032464
		(0.0113)	(0.0080)	(0.0061)		(0.0092)	(0.0090)	(0.0073)		(0.0104)	(0.0069)	(0.0066)		(0.0082)	(0.0103)	(0.0072)
${\theta }_{X,u}\left({\theta }_{X}\right)$		0.025426	− 0.020360	0.062743		− 0.008211	− 0.010144	0.078574		− 0.025338	− 0.016671	0.094697		− 0.012192	− 0.079394	0.061559
		(0.0168)	(0.0143)	(0.0087)		(0.0185)	(0.0158)	(0.0100)		(0.0179)	(0.0194)	(0.0132)		(0.0207)	(0.0199)	(0.0101)
${\theta }_{T,u}\left({\theta }_{T}\right)$		− 0.129356	− 0.049246	− 0.019262		− 0.095806	− 0.026469	− 0.010297		− 0.078990	− 0.019028	− 0.011279		− 0.069946	− 0.010386	− 0.006005
		(0.0083)	(0.0056)	(0.0028)		(0.0064)	(0.0029)	(0.0014)		(0.0053)	(0.0022)	(0.0017)		(0.0049)	(0.0016)	(0.0013)
${\theta }_{D,d}$				− 0.045663				− 0.041517				− 0.045810				− 0.057510
				(0.0065)				(0.0067)				(0.0089)				(0.0080)
${\theta }_{F,d}$				− 0.033269				− 0.043087				− 0.043641				− 0.071454
				(0.0069)				(0.0078)				(0.0067)				(0.0103)
${\theta }_{X,d}$				− 0.085976				− 0.097170				− 0.099785				− 0.142869
				(0.0099)				(0.0117)				(0.0106)				(0.0145)
${\theta }_{T,d}$				− 0.032791				− 0.020401				− 0.015589				− 0.007249
				(0.0054)				(0.0026)				(0.0018)				(0.0011)
${\nu }_{D,u}\left({\nu }_{D}\right)$		0.349882	0.303397	0.170164		0.386543	0.291435	0.179542		0.363802	0.358900	0.194860		0.340497	0.328360	0.158263
		(0.0200)	(0.0154)	(0.0156)		(0.0211)	(0.0153)	(0.0161)		(0.0230)	(0.0165)	(0.0160)		(0.0208)	(0.0169)	(0.0161)
${\nu }_{F,u}\left({\nu }_{F}\right)$		0.296938	0.287969	0.181850		0.295188	0.308004	0.189774		0.305827	0.298842	0.185792		0.274907	0.323946	0.150969
		(0.0195)	(0.0141)	(0.0146)		(0.0175)	(0.0143)	(0.0156)		(0.0197)	(0.0138)	(0.0143)		(0.0171)	(0.0141)	(0.0142)
${\nu }_{X,u}\left({\nu }_{X}\right)$		0.374565	0.310624	0.165345		0.368033	0.353823	0.194037		0.371654	0.340854	0.213995		0.348479	0.330604	0.150092
		(0.0159)	(0.0126)	(0.0131)		(0.0146)	(0.0131)	(0.0149)		(0.0139)	(0.0135)	(0.0154)		(0.0159)	(0.0132)	(0.0143)
${\nu }_{T,u}$		0.391352	0.222043	0.107949		0.364365	0.175146	0.081825		0.338708	0.155643	0.080438		0.324222	0.122733	0.055311
		(0.0112)	(0.0097)	(0.0094)		(0.0107)	(0.0085)	(0.0071)		(0.0107)	(0.0082)	(0.0075)		(0.0104)	(0.0072)	(0.0063)
${\nu }_{D,d}$				0.195819				0.190442				0.226936				0.240378
				(0.0169)				(0.0168)				(0.0178)				(0.0189)
${\nu }_{F,d}$				0.153181				0.176707				0.184648				0.224546
				(0.0142)				(0.0151)				(0.0143)				(0.0165)
${\nu }_{X,d}$				0.183590				0.204909				0.221071				0.225019
				(0.0147)				(0.0161)				(0.0159)				(0.0155)
${\nu }_{T,d}$				0.134098				0.116588				0.109734				0.084406
				(0.0110)				(0.0095)				(0.0087)				(0.0068)
${\phi }_{DF,u}\left({\phi }_{DF}\right)$			0.091636	0.017486			− 0.004755	0.023974			0.040313	0.022774			0.052980	0.024230
			(0.0352)	(0.0268)			(0.0380)	(0.0298)			(0.0403)	(0.0313)			(0.0393)	(0.0268)
${\phi }_{DX,u}\left({\phi }_{DX}\right)$			− 0.030493	− 0.060249			− 0.000962	− 0.030938			0.021785	− 0.020295			0.017621	0.003663
			(0.0322)	(0.0216)			(0.0340)	(0.0258)			(0.0347)	(0.0272)			(0.0350)	(0.0236)
${\phi }_{FX,u}\left({\phi }_{FX}\right)$			− 0.007026	− 0.018610			− 0.064709	− 0.063282			− 0.068927	− 0.072276			0.029509	− 0.040974
			(0.0307)	(0.0232)			(0.0338)	(0.0263)			(0.0361)	(0.0272)			(0.0340)	(0.0231)
${\phi }_{DF,d}$				0.018147				0.014756				0.013551				0.041549
				(0.0257)				(0.0286)				(0.0318)				(0.0314)
${\phi }_{DX,d}$				0.026804				− 0.010328				0.009262				0.010109
				(0.0233)				(0.0252)				(0.0266)				(0.0296)
${\phi }_{FX,d}$				0.059836				− 0.010387				0.001933				0.109213
				(0.0229)				(0.0249)				(0.0277)				(0.0264)
${\lambda }_{u}\left(\lambda \right)$		0.273904	0.217572	0.094420		0.296214	0.222632	0.100983		0.307797	0.257508	0.098539		0.344920	0.273139	0.089324
		(0.0085)	(0.0065)	(0.0069)		(0.0099)	(0.0074)	(0.0073)		(0.0096)	(0.0095)	(0.0072)		(0.0127)	(0.0092)	(0.0084)
${\lambda }_{d}$				0.120259				0.120065				0.140570				0.176357
				(0.0094)				(0.0098)				(0.0117)				(0.0121)
${h}_{D,u}\left({h}_{D}\right)$		− 0.016247	− 0.048444	− 0.033923		− 0.024678	− 0.045467	− 0.023176		− 0.002077	− 0.050795	− 0.027759		− 0.004745	− 0.037503	− 0.015255
		(0.0090)	(0.0072)	(0.0060)		(0.0089)	(0.0070)	(0.0052)		(0.0084)	(0.0089)	(0.0079)		(0.0086)	(0.0086)	(0.0065)
${h}_{F,u}\left({h}_{F}\right)$		0.019391	0.016288	0.012627		0.012761	0.026561	0.028398		− 0.018653	− 0.009754	0.009051		− 0.018498	− 0.049899	− 0.006722
		(0.0117)	(0.0108)	(0.0082)		(0.0108)	(0.0119)	(0.0103)		(0.0105)	(0.0114)	(0.0087)		(0.0104)	(0.0114)	(0.0084)
${h}_{X,u}\left({h}_{X}\right)$		0.005524	− 0.011550	− 0.013180		0.009795	0.011827	0.009233		0.021382	0.048543	0.035173		0.013918	0.016387	0.011592
		(0.0021)	(0.0042)	(0.0034)		(0.0023)	(0.0066)	(0.0055)		(0.0037)	(0.0090)	(0.0066)		(0.0033)	(0.0090)	(0.0063)
${h}_{D,d}$				− 0.020069				− 0.009306				0.003550				− 0.020309
				(0.0058)				(0.0067)				(0.0076)				(0.0065)
${h}_{F,d}$				0.004452				− 0.009602				− 0.019633				− 0.031435
				(0.0081)				(0.0094)				(0.0093)				(0.0093)
${h}_{X,d}$				0.001473				0.007825				0.030325				0.015973
				(0.0030)				(0.0049)				(0.0064)				(0.0067)
*Panel (c): Log-Likelihood*																
LL	2670.12	2800.71	3121.44	3172.58	2576.88	2627.62	3041.28	3122.81	2251.78	2387.07	2795.53	2882.96	1950.40	2225.34	2607.67	2670.89
LRT		261.18	641.46	102.29		101.49	827.30	163.07		270.58	816.91	174.86		549.88	764.65	126.45

The results are calibrated from the sample shown in Table 1. This table lists the average and standard error of the mean of parametric estimation results for GBP/USD. The results are divided into the continuous part (Panel (a)) and the jump part (Panel (b)). For the jump part’s parameters in CB–ISJ and CB–CSJ models, the notations are shown in the brackets. For example, the mean of jump amplitude for domestic interest rate in CB–ISJ and CB–CSJ models is ${\uptheta }_{\mathrm{D}}$ in the bracket

**Table 4:** Parametric estimation results for JPY/USD

	1-Month				3-Month				6-Month				12-Month			
	CB	CB–ISJ	CB–CSJ	CB–CAJ	CB	CB–ISJ	CB–CSJ	CB–CAJ	CB	CB–ISJ	CB–CSJ	CB–CAJ	CB	CB–ISJ	CB–CSJ	CB–CAJ
*Panel (a): Continuous Part*																
${\mu }_{D}$	− 0.000724	0.002790	0.000202	− 0.000345	− 0.000140	0.003769	0.000163	− 0.000201	− 0.000025	0.006647	0.001585	− 0.000752	0.002471	0.004365	0.003167	− 0.001568
	(0.0006)	(0.0011)	(0.0004)	(0.0002)	(0.0003)	(0.0012)	(0.0005)	(0.0004)	(0.0001)	(0.0015)	(0.0011)	(0.0005)	(0.0006)	(0.0014)	(0.0017)	(0.0004)
${\mu }_{F}$	− 0.002540	− 0.013534	− 0.013198	− 0.013227	− 0.003591	− 0.014064	− 0.014798	− 0.014493	− 0.005207	− 0.014572	− 0.016581	− 0.016151	− 0.005799	− 0.017797	− 0.018464	− 0.018679
	(0.0007)	(0.0015)	(0.0011)	(0.0011)	(0.0007)	(0.0013)	(0.0013)	(0.0012)	(0.0009)	(0.0015)	(0.0014)	(0.0013)	(0.0009)	(0.0016)	(0.0016)	(0.0016)
${\mu }_{X}$	0.012065	0.074566	0.037860	0.037697	0.012294	0.062645	0.037402	0.034098	0.008886	0.058460	0.037401	0.032931	0.011999	0.020883	0.009416	0.014147
	(0.0019)	(0.0064)	(0.0032)	(0.0032)	(0.0018)	(0.0067)	(0.0037)	(0.0035)	(0.0011)	(0.0079)	(0.0051)	(0.0046)	(0.0017)	(0.0091)	(0.0047)	(0.0034)
${\sigma }_{D}$	0.001283	0.045123	0.010410	0.004763	0.000387	0.050116	0.012271	0.006201	0.000643	0.062721	0.027526	0.007445	0.027741	0.050773	0.032656	0.007195
	(0.0010)	(0.0061)	(0.0028)	(0.0017)	(0.0002)	(0.0066)	(0.0032)	(0.0022)	(0.0002)	(0.0074)	(0.0051)	(0.0024)	(0.0067)	(0.0070)	(0.0067)	(0.0021)
${\sigma }_{F}$	0.001508	0.016329	0.001445	0.001331	0.000761	0.014872	0.004829	0.001694	0.001221	0.018566	0.004784	0.000251	0.014728	0.012520	0.004372	0.001172
	(0.0011)	(0.0049)	(0.0006)	(0.0006)	(0.0002)	(0.0042)	(0.0019)	(0.0009)	(0.0002)	(0.0047)	(0.0019)	(0.0001)	(0.0032)	(0.0038)	(0.0017)	(0.0007)
${\sigma }_{X}$	0.006340	0.249889	0.117325	0.109459	0.005559	0.213643	0.090310	0.074485	0.006006	0.181977	0.112538	0.059714	0.033455	0.156570	0.076310	0.039318
	(0.0005)	(0.0128)	(0.0066)	(0.0066)	(0.0001)	(0.0132)	(0.0070)	(0.0061)	(0.0002)	(0.0138)	(0.0098)	(0.0063)	(0.0067)	(0.0142)	(0.0080)	(0.0042)
${\rho }_{DF}$	0.033618	− 0.046936	0.117929	0.103691	0.027450	− 0.069172	0.033432	0.070554	0.015880	− 0.163274	− 0.008536	− 0.078817	0.014507	− 0.078124	− 0.015887	− 0.015277
	(0.0234)	(0.0439)	(0.0364)	(0.0367)	(0.0209)	(0.0415)	(0.0326)	(0.0326)	(0.0232)	(0.0405)	(0.0356)	(0.0344)	(0.0222)	(0.0405)	(0.0324)	(0.0301)
${\rho }_{DX}$	− 0.100361	0.245872	0.319529	0.325771	− 0.135105	0.272584	0.251802	0.246466	− 0.146818	0.184796	0.221529	0.196845	− 0.254027	0.197142	0.235278	0.200726
	(0.0199)	(0.0310)	(0.0297)	(0.0297)	(0.0202)	(0.0307)	(0.0318)	(0.0313)	(0.0196)	(0.0337)	(0.0334)	(0.0337)	(0.0248)	(0.0320)	(0.0308)	(0.0311)
${\rho }_{FX}$	0.030153	− 0.203839	− 0.360288	− 0.387780	− 0.064767	− 0.219899	− 0.353414	− 0.368244	− 0.016641	− 0.117808	− 0.343492	− 0.348256	0.062092	− 0.215963	− 0.355031	− 0.331898
	(0.0173)	(0.0303)	(0.0258)	(0.0250)	(0.0232)	(0.0315)	(0.0289)	(0.0266)	(0.0239)	(0.0332)	(0.0312)	(0.0295)	(0.0286)	(0.0311)	(0.0284)	(0.0264)
${h}_{1,D}$	0.048652	− 0.144661	− 0.048426	− 0.041649	0.034390	− 0.111108	− 0.033412	− 0.024636	0.049456	− 0.091031	− 0.049862	− 0.033178	0.090390	− 0.089294	− 0.037697	− 0.030640
	(0.0049)	(0.0099)	(0.0129)	(0.0129)	(0.0107)	(0.0108)	(0.0126)	(0.0125)	(0.0123)	(0.0117)	(0.0138)	(0.0140)	(0.0137)	(0.0121)	(0.0123)	(0.0127)
${h}_{1,F}$	0.015571	− 0.111249	− 0.036274	− 0.028987	− 0.012991	− 0.097861	− 0.018851	− 0.015554	− 0.021189	− 0.068303	− 0.012708	− 0.016123	− 0.034405	− 0.065312	− 0.021774	− 0.014086
	(0.0060)	(0.0132)	(0.0130)	(0.0127)	(0.0117)	(0.0125)	(0.0127)	(0.0127)	(0.0112)	(0.0131)	(0.0137)	(0.0137)	(0.0151)	(0.0139)	(0.0133)	(0.0136)
${h}_{1,X}$	0.026452	0.015471	0.018559	0.018534	0.039785	− 0.017453	0.009041	0.007072	0.035143	0.009824	− 0.003340	0.005323	0.082119	0.033215	0.009578	0.005886
	(0.0054)	(0.0121)	(0.0111)	(0.0110)	(0.0049)	(0.0120)	(0.0109)	(0.0107)	(0.0057)	(0.0125)	(0.0120)	(0.0119)	(0.0103)	(0.0129)	(0.0124)	(0.0127)
*Panel (b): Jump Part*																
${\theta }_{D,u}\left({\theta }_{D}\right)$		− 0.077217	− 0.040962	− 0.026035		− 0.096232	− 0.046493	− 0.020441		− 0.095027	− 0.092940	− 0.047002		− 0.096997	− 0.091239	− 0.039089
		(0.0113)	(0.0053)	(0.0044)		(0.0127)	(0.0046)	(0.0038)		(0.0117)	(0.0100)	(0.0079)		(0.0115)	(0.0088)	(0.0079)
${\theta }_{F,u}\left({\theta }_{F}\right)$		− 0.139962	− 0.118199	− 0.067298		− 0.149719	− 0.152991	− 0.081523		− 0.120970	− 0.163119	− 0.088605		− 0.130385	− 0.132831	− 0.062873
		(0.0122)	(0.0104)	(0.0093)		(0.0138)	(0.0112)	(0.0095)		(0.0119)	(0.0113)	(0.0102)		(0.0113)	(0.0110)	(0.0094)
${\theta }_{X,u}\left({\theta }_{X}\right)$		0.016231	0.018971	0.064418		0.003326	0.019707	0.064708		− 0.018151	− 0.015685	0.065297		− 0.088590	− 0.077671	0.040334
		(0.0178)	(0.0102)	(0.0079)		(0.0188)	(0.0123)	(0.0081)		(0.0213)	(0.0161)	(0.0101)		(0.0245)	(0.0144)	(0.0084)
${\theta }_{T,u}\left({\theta }_{T}\right)$		− 0.106679	− 0.031547	− 0.021507		− 0.101126	− 0.032968	− 0.019479		− 0.080597	− 0.023948	− 0.008753		− 0.065351	− 0.012870	− 0.006549
		(0.0073)	(0.0033)	(0.0032)		(0.0066)	(0.0038)	(0.0035)		(0.0056)	(0.0032)	(0.0014)		(0.0055)	(0.0016)	(0.0012)
${\theta }_{D,d}$				− 0.018009				− 0.021483				− 0.048310				− 0.049949
				(0.0039)				(0.0041)				(0.0080)				(0.0068)
${\theta }_{F,d}$				− 0.042573				− 0.060715				− 0.075859				− 0.074966
				(0.0078)				(0.0085)				(0.0093)				(0.0111)
${\theta }_{X,d}$				− 0.046338				− 0.061940				− 0.091672				− 0.125535
				(0.0058)				(0.0077)				(0.0095)				(0.0115)
${\theta }_{T,d}$				− 0.015228				− 0.018851				− 0.020360				− 0.014544
				(0.0021)				(0.0030)				(0.0024)				(0.0018)
${\nu }_{D,u}\left({\nu }_{D}\right)$		0.307731	0.235797	0.151557		0.365046	0.249631	0.161195		0.336846	0.354567	0.232892		0.347175	0.340592	0.186919
		(0.0203)	(0.0128)	(0.0134)		(0.0202)	(0.0119)	(0.0121)		(0.0205)	(0.0185)	(0.0181)		(0.0197)	(0.0168)	(0.0172)
${\nu }_{F,u}\left({\nu }_{F}\right)$		0.269488	0.231884	0.152615		0.283391	0.264876	0.180132		0.262703	0.292638	0.216453		0.283342	0.289277	0.176559
		(0.0179)	(0.0112)	(0.0127)		(0.0179)	(0.0124)	(0.0141)		(0.0158)	(0.0135)	(0.0153)		(0.0160)	(0.0137)	(0.0149)
${\nu }_{X,u}\left({\nu }_{X}\right)$		0.341559	0.264691	0.169215		0.350813	0.305284	0.201426		0.354049	0.318202	0.207832		0.348720	0.299622	0.153921
		(0.0149)	(0.0097)	(0.0124)		(0.0154)	(0.0115)	(0.0133)		(0.0167)	(0.0129)	(0.0146)		(0.0163)	(0.0126)	(0.0134)
${\nu }_{T,u}$		0.372766	0.189095	0.120676		0.368955	0.188932	0.115260		0.341030	0.154653	0.074490		0.307825	0.140148	0.056371
		(0.0110)	(0.0088)	(0.0099)		(0.0106)	(0.0106)	(0.0105)		(0.0110)	(0.0093)	(0.0063)		(0.0120)	(0.0078)	(0.0061)
${\nu }_{D,d}$				0.126384				0.131621				0.248069				0.240878
				(0.0125)				(0.0126)				(0.0176)				(0.0175)
${\nu }_{F,d}$				0.122497				0.143187				0.206913				0.218347
				(0.0117)				(0.0132)				(0.0145)				(0.0149)
${\nu }_{X,d}$				0.129250				0.143826				0.193444				0.199804
				(0.0111)				(0.0128)				(0.0140)				(0.0139)
${\nu }_{T,d}$				0.096627				0.098558				0.120820				0.113042
				(0.0089)				(0.0098)				(0.0097)				(0.0084)
${\phi }_{DF,u}\left({\phi }_{DF}\right)$			0.063737	0.028103			0.075084	0.015236			0.088882	0.039773			0.096012	0.018120
			(0.0317)	(0.0251)			(0.0318)	(0.0255)			(0.0357)	(0.0277)			(0.0364)	(0.0248)
${\phi }_{DX,u}\left({\phi }_{DX}\right)$			− 0.002817	− 0.006861			0.022876	0.014827			0.001231	− 0.037128			0.056436	− 0.020936
			(0.0321)	(0.0243)			(0.0339)	(0.0253)			(0.0359)	(0.0287)			(0.0333)	(0.0244)
${\phi }_{FX,u}\left({\phi }_{FX}\right)$			− 0.039602	− 0.032789			− 0.077907	− 0.053047			− 0.050654	− 0.113436			− 0.010334	− 0.083805
			(0.0304)	(0.0229)			(0.0312)	(0.0244)			(0.0333)	(0.0258)			(0.0347)	(0.0241)
${\phi }_{DF,d}$				0.020391				0.010796				0.020712				0.035938
				(0.0232)				(0.0247)				(0.0285)				(0.0325)
${\phi }_{DX,d}$				− 0.025167				− 0.020794				0.000596				0.016750
				(0.0224)				(0.0232)				(0.0282)				(0.0288)
${\phi }_{FX,d}$				0.003330				0.021847				0.023164				0.045226
				(0.0227)				(0.0224)				(0.0275)				(0.0291)
${\lambda }_{u}\left(\lambda \right)$		0.275868	0.199275	0.102557		0.284816	0.192973	0.104484		0.300577	0.246232	0.101458		0.333489	0.277182	0.088177
		(0.0083)	(0.0062)	(0.0073)		(0.0095)	(0.0066)	(0.0069)		(0.0091)	(0.0089)	(0.0076)		(0.0119)	(0.0077)	(0.0074)
${\lambda }_{d}$				0.096512				0.085225				0.120717				0.166303
				(0.0085)				(0.0077)				(0.0090)				(0.0108)
${h}_{D,u}\left({h}_{D}\right)$		− 0.006271	− 0.049757	− 0.030428		− 0.006466	− 0.052215	− 0.034054		− 0.011305	− 0.048651	− 0.022894		− 0.014017	− 0.046253	− 0.023847
		(0.0086)	(0.0070)	(0.0060)		(0.0088)	(0.0072)	(0.0064)		(0.0090)	(0.0094)	(0.0074)		(0.0092)	(0.0079)	(0.0073)
${h}_{F,u}\left({h}_{F}\right)$		0.009905	0.052307	0.043514		0.024171	0.024264	0.042562		0.032573	-0.009302	0.014825		− 0.013567	− 0.029517	0.019235
		(0.0108)	(0.0097)	(0.0082)		(0.0111)	(0.0104)	(0.0094)		(0.0107)	(0.0114)	(0.0090)		(0.0118)	(0.0124)	(0.0091)
${h}_{X,u}\left({h}_{X}\right)$		0.000244	− 0.016493	− 0.015157		0.010492	0.017190	− 0.003271		0.016237	0.047250	0.016391		0.011176	− 0.004335	0.002691
		(0.0022)	(0.0044)	(0.0030)		(0.0026)	(0.0057)	(0.0048)		(0.0035)	(0.0080)	(0.0064)		(0.0038)	(0.0091)	(0.0063)
${h}_{D,d}$				− 0.020347				− 0.006948				− 0.003349				− 0.016719
				(0.0044)				(0.0067)				(0.0081)				(0.0074)
${h}_{F,d}$				0.018355				− 0.007953				0.011487				− 0.045573
				(0.0064)				(0.0082)				(0.0092)				(0.0098)
${h}_{X,d}$				− 0.005902				0.015773				0.035390				0.010974
				(0.0035)				(0.0040)				(0.0057)				(0.0063)
*Panel (c): Log-Likelihood*																
LL	2409.87	2646.89	3080.72	3131.02	2423.00	2585.50	3003.17	3058.59	2400.09	2465.06	2628.32	2809.77	2372.92	2393.51	2563.52	2679.95
LRT		474.05	867.66	100.59		325.00	835.34	110.84		129.95	326.52	362.89		41.17	340.03	232.86

The results are calibrated form the sample shown as Table 1. This table lists the average and standard error of the mean of parametric estimation results for JPY/USD. The results are divided into the continuous part (Panel (a)) and the jump part (Panel (b)). For the jump part’s parameters in CB–ISJ and CB–CSJ models, the notations are shown in the brackets. For example, the mean of jump amplitude for domestic interest rate in CB–ISJ and CB–CSJ models is ${\uptheta }_{\mathrm{D}}$ in the bracket

**Table 5:** Pricing performance of EUR/USD options

	In-sample					Out-of-sample				
	1-Month	3-Month	6-Month	12-Month	Average	1-Month	3-Month	6-Month	12-Month	Average
*10-delta*										
GK	0.000350	0.000328	0.000350	0.000434	0.000366	0.000564	0.000491	0.000469	0.000529	0.000513
CB	0.001074	0.001147	0.001582	0.001150	0.001239	0.001179	0.001192	0.001527	0.001083	0.001245
CB–ISJ	**0.000091**	**0.000087**	**0.000087**	**0.000102**	**0.000092**	**0.000232**	**0.000176**	**0.000149**	**0.000139**	**0.000174**
CB–CSJ	0.000102	0.000093	0.000120	0.000206	0.000131	0.000241	0.000179	0.000172	0.000257	0.000212
CB–CAJ	0.000096	0.000096	0.000100	0.000131	0.000106	0.000239	0.000193	0.000156	0.000181	0.000192
*25-delta*										
GK	0.000395	0.000371	0.000393	0.000429	0.000397	0.000603	0.000525	0.000504	0.000512	0.000536
CB	0.001175	0.001263	0.001787	0.001404	0.001407	0.001283	0.001309	0.001739	0.001341	0.001418
CB–ISJ	**0.000076**	**0.000078**	**0.000082**	**0.000093**	**0.000082**	**0.000221**	**0.000166**	**0.000140**	**0.000126**	**0.000163**
CB–CSJ	0.000091	0.000081	0.000109	0.000202	0.000121	0.000229	0.000166	0.000157	0.000251	0.000201
CB–CAJ	0.000081	0.000079	0.000091	0.000129	0.000095	0.000227	0.000173	0.000143	0.000176	0.000180
*50-delta*										
GK	0.000411	0.000385	0.000419	0.000429	0.000411	0.000633	0.000549	0.000532	0.000506	0.000555
CB	0.001213	0.001323	0.001817	0.001428	0.001445	0.001329	0.001361	0.001770	0.001367	0.001457
CB–ISJ	**0.000065**	0.000058	**0.000056**	**0.000063**	**0.000060**	**0.000224**	0.000152	**0.000117**	**0.000096**	**0.000147**
CB–CSJ	0.000086	0.000058	0.000082	0.000170	0.000099	0.000234	**0.000149**	0.000132	0.000223	0.000185
CB–CAJ	0.000069	**0.000056**	0.000063	0.000102	0.000072	0.000230	0.000155	0.000117	0.000151	0.000163
*75-delta*										
GK	0.000386	0.000381	0.000451	0.000462	0.000420	0.000639	0.000570	0.000576	0.000541	0.000581
CB	0.001157	0.001333	0.001675	0.001235	0.001350	0.001285	0.001362	0.001620	0.001168	0.001359
CB–ISJ	**0.000072**	0.000053	**0.000043**	**0.000044**	**0.000053**	**0.000255**	0.000161	0.000113	**0.000083**	**0.000153**
CB–CSJ	0.000102	**0.000052**	0.000077	0.000140	0.000093	0.000271	**0.000158**	0.000134	0.000203	0.000191
CB–CAJ	0.000075	0.000056	0.000049	0.000081	0.000065	0.000260	0.000169	**0.000110**	0.000136	0.000169
*90-delta*										
GK	0.000434	0.000549	0.000752	0.000866	0.000650	0.000729	0.000775	0.000900	0.000955	0.000840
CB	0.001146	0.001482	0.001682	0.001216	0.001382	0.001288	0.001503	0.001618	0.001141	0.001387
CB–ISJ	**0.000174**	0.000209	**0.000247**	**0.000272**	**0.000225**	**0.000383**	0.000334	0.000330	**0.000322**	**0.000343**
CB–CSJ	0.000211	**0.000206**	0.000287	0.000353	0.000264	0.000406	**0.000331**	0.000358	0.000431	0.000381
CB–CAJ	0.000174	0.000220	0.000249	0.000304	0.000237	0.000388	0.000354	**0.000323**	0.000369	0.000358

This table lists the MSEs of different maturity, delta levels, and models with in-sample and out-of-sample results for the currency pair EUR/USD. The left panel shows the in-sample test results and the right panel shows the out-of-sample test results. The bold text shows the results of the best-performing model among the five models with the same maturity and delta level

**Table 6:** Pricing performance of GBP/USD options

	In-sample					Out-of-sample				
	1-Month	3-Month	6-Month	12-Month	Average	1-Month	3-Month	6-Month	12-Month	Average
*10-delta*										
GK	0.000830	0.000814	0.000722	**0.000600**	0.000741	0.001244	0.001007	0.000853	**0.000692**	0.000949
CB	0.001670	0.000988	0.001453	0.001242	0.001338	0.001809	0.001040	0.001462	0.001266	0.001394
CB–ISJ	**0.000203**	**0.000413**	0.036214	0.025415	0.015561	**0.000414**	**0.000520**	0.033950	0.024638	0.014880
CB–CSJ	0.000409	0.000528	0.000704	0.000915	0.000639	0.000559	0.000589	0.000763	0.000983	0.000723
CB–CAJ	0.000399	0.000495	**0.000556**	0.000755	**0.000551**	0.000570	0.000586	**0.000601**	0.000823	**0.000645**
*25-delta*										
GK	0.000904	0.000919	0.000848	**0.000713**	0.000846	0.001330	0.001120	0.000978	**0.000796**	0.001056
CB	0.001770	0.001087	0.001606	0.001461	0.001481	0.001912	0.001133	0.001616	0.001483	0.001536
CB–ISJ	**0.000197**	**0.000410**	0.036412	0.025534	0.015638	**0.000427**	**0.000526**	0.034061	0.024750	0.014941
CB–CSJ	0.000424	0.000548	0.000741	0.000969	0.000670	0.000579	0.000603	0.000797	0.001028	0.000752
CB–CAJ	0.000414	0.000519	**0.000581**	0.000807	**0.000580**	0.000592	0.000601	**0.000627**	0.000866	**0.000671**
*50-delta*										
GK	0.000885	0.000898	0.000837	**0.000690**	0.000828	0.001354	0.001126	0.000980	**0.000776**	0.001059
CB	0.001779	0.001084	0.001530	0.001385	0.001444	0.001923	0.001126	0.001535	0.001409	0.001498
CB–ISJ	**0.000183**	**0.000368**	0.036189	0.025489	0.015557	**0.000452**	**0.000500**	0.033851	0.024692	0.014874
CB–CSJ	0.000449	0.000537	0.000696	0.000935	0.000654	0.000617	0.000594	0.000756	0.000992	0.000740
CB–CAJ	0.000437	0.000510	**0.000543**	0.000762	**0.000563**	0.000630	0.000594	**0.000591**	0.000819	**0.000659**
*75-delta*										
GK	0.000788	0.000824	0.000786	**0.000652**	0.000763	0.001316	0.001097	0.000954	**0.000750**	0.001029
CB	0.001692	0.001051	0.001309	0.001128	0.001295	0.001834	0.001083	0.001293	0.001156	0.001342
CB–ISJ	**0.000215**	**0.000369**	0.035539	0.025311	0.015359	**0.000535**	**0.000522**	0.033317	0.024484	0.014714
CB–CSJ	0.000527	0.000575	0.000655	0.000911	0.000667	0.000708	0.000640	0.000725	0.000968	0.000760
CB–CAJ	0.000511	0.000548	**0.000532**	0.000716	**0.000577**	0.000722	0.000640	**0.000586**	0.000775	**0.000681**
*90-delta*										
GK	0.000798	0.000973	0.001055	0.001042	0.000967	0.001395	0.001302	0.001258	0.001162	0.001279
CB	0.001709	0.001254	0.001352	0.001181	0.001374	0.001853	0.001281	0.001308	0.001215	0.001414
CB–ISJ	**0.000397**	**0.000612**	0.034955	0.025407	0.015343	**0.000773**	**0.000784**	0.032937	0.024553	0.014762
CB–CSJ	0.000750	0.000841	0.000888	0.001200	0.000919	0.000948	0.000925	0.000973	0.001265	0.001028
CB–CAJ	0.000730	0.000813	**0.000810**	**0.000979**	**0.000833**	0.000962	0.000925	**0.000873**	**0.001047**	**0.000952**

This table lists the MSEs of different maturity, delta levels, and models with in-sample and out-of-sample results for the currency pair GBP/USD. The left panel shows the in-sample test results and the right panel shows the out-of-sample test results. The bold text shows the results of the best-performing model among the five models with the same maturity and delta level

**Table 7:** Pricing performance of JPY/USD options

	In-sample					Out-of-sample				
	1-Month	3-Month	6-Month	12-Month	Average	1-Month	3-Month	6-Month	12-Month	Average
*10-delta*										
GK	0.000669	**0.000723**	0.000759	**0.000743**	**0.000724**	0.000995	**0.000855**	0.000818	**0.000763**	**0.000858**
CB	0.001250	0.001200	0.001755	0.001384	0.001397	0.001434	0.001246	0.001781	0.001369	0.001457
CB–ISJ	**0.000227**	0.002385	0.000337	0.002112	0.001265	**0.000432**	0.002578	0.000369	0.002043	0.001355
CB–CSJ	0.000534	0.009267	**0.000263**	0.005966	0.004008	0.000707	0.009782	**0.000324**	0.005458	0.004068
CB–CAJ	0.000427	0.009372	0.000265	0.005825	0.003972	0.000577	0.009875	0.000332	0.005338	0.004030
*25-delta*										
GK	0.000669	**0.000716**	0.000736	**0.000711**	**0.000708**	0.001021	**0.000855**	0.000795	**0.000733**	**0.000851**
CB	0.001240	0.001151	0.001750	0.001420	0.001390	0.001450	0.001211	0.001772	0.001411	0.001461
CB–ISJ	**0.000205**	0.002349	0.000321	0.002210	0.001271	**0.000437**	0.002548	0.000358	0.002139	0.001370
CB–CSJ	0.000536	0.009268	0.000262	0.006045	0.004028	0.000742	0.009787	**0.000331**	0.005553	0.004103
CB–CAJ	0.000433	0.009370	**0.000262**	0.005894	0.003990	0.000611	0.009888	0.000333	0.005416	0.004062
*50-delta*										
GK	0.000564	**0.000524**	0.000482	**0.000434**	**0.000501**	0.000972	**0.000702**	0.000560	**0.000475**	**0.000677**
CB	0.001062	0.000874	0.001286	0.000910	0.001033	0.001303	0.000962	0.001336	0.000929	0.001133
CB–ISJ	**0.000140**	0.002194	0.000188	0.002150	0.001168	**0.000423**	0.002407	0.000250	0.002086	0.001291
CB–CSJ	0.000533	0.009125	0.000146	0.005922	0.003931	0.000810	0.009637	0.000244	0.005469	0.004040
CB–CAJ	0.000444	0.009230	**0.000138**	0.005766	0.003894	0.000684	0.009757	**0.000241**	0.005320	0.004001
*75-delta*										
GK	0.000504	**0.000396**	0.000331	**0.000329**	**0.000390**	0.000980	**0.000625**	0.000434	**0.000392**	**0.000608**
CB	0.000907	0.000674	0.000840	0.000419	0.000710	0.001183	0.000789	0.000920	0.000474	0.000842
CB–ISJ	**0.000179**	0.002123	0.000199	0.002153	0.001163	**0.000522**	0.002352	0.000289	0.002103	0.001317
CB–CSJ	0.000652	0.009038	0.000174	0.005857	0.003930	0.001017	0.009543	0.000305	0.005443	0.004077
CB–CAJ	0.000581	0.009144	**0.000149**	0.005719	0.003898	0.000899	0.009687	**0.000289**	0.005308	0.004046
*90-delta*										
GK	0.000786	**0.000780**	0.000885	0.001239	0.000923	0.001332	**0.001078**	0.001027	0.001338	0.001194
CB	0.001067	0.000966	0.000924	**0.000681**	**0.000910**	0.001365	0.001107	0.001042	**0.000790**	**0.001076**
CB–ISJ	**0.000552**	0.002510	0.000802	0.002852	0.001679	**0.000957**	0.002755	**0.000930**	0.002825	0.001867
CB–CSJ	0.001123	0.009391	0.000792	0.006461	0.004442	0.001595	0.009886	0.000966	0.006097	0.004636
CB–CAJ	0.001074	0.009515	**0.000743**	0.006345	0.004419	0.001488	0.010075	0.000936	0.005978	0.004619

This table lists the MSEs of different maturity, delta levels, and models with in-sample and out-of-sample results for the currency pair JPY/USD. The left panel shows the in-sample test results and the right panel shows the out-of-sample test results. The bold text shows the results of the best-performing model among the five models with the same maturity and delta level

**Table 8:** Risk premia regression results for EUR/USD

Year	Maturity	Adj.${R}^{2}$	$\alpha$	${\beta }_{1}$	${\beta }_{2}$	${\beta }_{3}$	${\beta }_{4}$	Year	Maturity	Adj.${R}^{2}$	$\alpha$	${\beta }_{1}$	${\beta }_{2}$	${\beta }_{3}$	${\beta }_{4}$
2007	1 M	0.09	0.0001***	− 0.0822***	0.0001***	0.0004***	− 0.0008***	2013	1 M	0.00	0.0004***	− 0.1419***	0.0004***	0.0049***	− 0.0020***
			(0.59)	(0.00)	(0.73)	(0.51)	(0.48)				(0.13)	(0.47)	(0.40)	(0.24)	(0.10)
	3 M	0.51	− 0.0001***	− 0.2260***	0.0000***	− 0.0001***	0.0009***		3 M	0.02	0.0003***	− 0.3522***	0.0011***	0.0000***	0.0000***
			(0.49)	(0.00)	(0.93)	(0.84)	(0.51)				(0.09)	(0.03)	(0.64)	(0.93)	(0.93)
	6 M	0.83	0.0002***	− 0.4853***	− 0.0004***	0.0005***	− 0.0009***		6 M	0.13	0.0004***	− 0.5343***	0.0001***	− 0.0001***	− 0.0035***
			(0.32)	(0.00)	(0.30)	(0.80)	(0.62)				(0.01)	(0.00)	(0.84)	(0.82)	(0.41)
	12 M	0.95	− 0.0004***	− 0.8766***	− 0.0005***	− 0.0008***	0.0009***		12 M	0.08	0.0009***	− 0.7232***	− 0.0061***	0.0086***	− 0.0273***
			(0.00)	(0.00)	(0.26)	(0.23)	(0.39)				(0.53)	(0.19)	(0.15)	(0.00)	(0.04)
2008	1 M	0.08	− 0.0006***	− 0.1158***	− 0.0004***	0.0018***	0.0107***	2014	1 M	− 0.01	0.0001***	− 0.0086***	0.0003***	− 0.0014***	0.0026***
			(0.13)	(0.00)	(0.65)	(0.25)	(0.09)				(0.08)	(0.89)	(0.41)	(0.30)	(0.46)
	3 M	0.31	− 0.0005***	− 0.2504***	− 0.0013***	0.0035***	0.0002***		3 M	0.07	0.0001***	− 0.2125***	− 0.0006***	0.0001***	− 0.0013***
			(0.33)	(0.00)	(0.25)	(0.00)	(0.96)				(0.15)	(0.00)	(0.16)	(0.80)	(0.37)
	6 M	0.50	− 0.0031***	− 0.5847***	0.0074***	− 0.0045***	0.0009***		6 M	0.26	0.0000***	− 0.5593***	0.0009***	− 0.0009***	− 0.0036***
			(0.00)	(0.00)	(0.00)	(0.01)	(0.80)				(0.99)	(0.00)	(0.15)	(0.07)	(0.17)
	12 M	0.64	− 0.0066***	− 1.2075***	− 0.0002***	0.0110***	− 0.0134***		12 M	0.44	− 0.0026***	− 3.2653***	− 0.0156***	− 0.0132***	− 0.0003***
			(0.00)	(0.00)	(0.91)	(0.00)	(0.00)				(0.00)	(0.00)	(0.00)	(0.01)	(0.97)
2009	1 M	0.02	0.0000***	− 0.0733***	− 0.0001***	0.0017***	− 0.0027***	2015	1 M	0.03	0.0007***	− 0.2706***	0.0011***	− 0.0020***	0.0005***
			(0.95)	(0.02)	(0.92)	(0.06)	(0.56)				(0.10)	(0.03)	(0.29)	(0.09)	(0.85)
	3 M	0.09	− 0.0007***	− 0.3060***	− 0.0045***	0.0074***	− 0.0017***		3 M	0.06	0.0003***	− 0.4154***	0.0001***	− 0.0001***	− 0.0002***
			(0.06)	(0.00)	(0.12)	(0.06)	(0.34)				(0.40)	(0.00)	(0.94)	(0.96)	(0.98)
	6 M	0.21	− 0.0009***	− 0.4702***	− 0.0013***	0.0007***	0.0092***		6 M	0.22	− 0.0002***	− 0.6391***	0.0017***	− 0.0002***	0.0045***
			(0.01)	(0.00)	(0.12)	(0.53)	(0.01)				(0.57)	(0.00)	(0.01)	(0.86)	(0.04)
	12 M	0.09	0.0009***	− 0.7458***	− 0.0040***	0.0134***	0.0106***		12 M	0.46	− 0.0010***	− 1.6720***	0.0629***	− 0.0601***	0.0487***
			(0.17)	(0.00)	(0.21)	(0.02)	(0.13)				(0.73)	(0.00)	(0.00)	(0.00)	(0.00)
2010	1 M	0.00	0.0000***	− 0.0311***	− 0.0002***	− 0.0004***	0.0053***	2016	1 M	− 0.01	0.0007***	− 0.0805***	0.0001***	0.0022***	− 0.0017***
			(0.86)	(0.62)	(0.83)	(0.77)	(0.21)				(0.26)	(0.29)	(0.92)	(0.60)	(0.38)
	3 M	0.00	− 0.0002***	− 0.1039***	0.0000***	0.0014***	0.0021***		3 M	0.00	− 0.0002***	− 0.0231***	− 0.0012***	0.0000***	− 0.0001***
			(0.33)	(0.05)	(0.99)	(0.42)	(0.27)				(0.79)	(0.69)	(0.10)	(0.99)	(0.97)
	6 M	0.05	− 0.0009***	− 0.1920***	0.0000***	0.0008***	− 0.0012***		6 M	0.07	0.0071***	− 0.8105***	0.0005***	− 0.0001***	0.0054***
			(0.01)	(0.00)	(0.96)	(0.28)	(0.40)				(0.00)	(0.00)	(0.84)	(0.96)	(0.48)
	12 M	0.21	− 0.0046***	− 0.6581***	0.0009***	0.0025***	0.0312***		12 M	0.18	0.0396***	− 4.0744***	0.0010***	− 0.0140***	0.0073***
			(0.00)	(0.00)	(0.71)	(0.26)	(0.00)				(0.00)	(0.00)	(0.91)	(0.26)	(0.52)
2011	1 M	0.03	− 0.0005***	− 0.1130***	− 0.0002***	0.0017***	− 0.0033***	2017	1 M	− 0.01	0.0000***	− 0.0014***	0.0001***	0.0005***	− 0.0014***
			(0.46)	(0.14)	(0.81)	(0.15)	(0.23)				(0.96)	(0.97)	(0.89)	(0.79)	(0.79)
	3 M	0.11	− 0.0021***	− 0.3506***	− 0.0017***	0.0014***	− 0.0011***		3 M	0.00	− 0.0016***	0.0558***	− 0.0004***	− 0.0003***	0.0017***
			(0.00)	(0.00)	(0.04)	(0.26)	(0.42)				(0.11)	(0.34)	(0.60)	(0.78)	(0.10)
	6 M	0.39	− 0.0071***	− 1.0330***	0.0033***	− 0.0003***	0.0117***		6 M	0.02	− 0.0017***	0.0970***	− 0.0017***	− 0.0155***	0.0070***
			(0.00)	(0.00)	(0.00)	(0.82)	(0.00)				(0.83)	(0.83)	(0.68)	(0.01)	(0.41)
	12 M	0.31	0.0100***	0.0615***	0.0220***	0.0026***	− 0.0314***		12 M	0.35	− 0.3021***	15.4227***	− 0.0274***	− 0.0222***	− 0.0509***
			(0.00)	(0.82)	(0.00)	(0.55)	(0.00)				(0.00)	(0.00)	(0.02)	(0.32)	(0.02)
2012	1 M	− 0.01	− 0.0001***	− 0.0163***	− 0.0007***	− 0.0004***	− 0.0022***	2018	1 M	0.20	0.0051***	− 0.2499***	0.0005***	− 0.0022***	− 0.0079***
			(0.52)	(0.71)	(0.20)	(0.64)	(0.50)				(0.00)	(0.00)	(0.47)	(0.10)	(0.01)
	3 M	0.01	− 0.0005***	− 0.0726***	0.0008***	− 0.0007***	0.0024***		3 M	0.33	0.0176***	− 0.8007***	0.0007***	0.0015***	− 0.0073***
			(0.00)	(0.03)	(0.18)	(0.63)	(0.54)				(0.00)	(0.00)	(0.57)	(0.33)	(0.00)
	6 M	0.09	− 0.0013***	− 0.1153***	− 0.0015***	0.0010***	− 0.0034***		6 M	0.13	0.0311***	− 1.4204***	0.0221***	0.0031***	− 0.0451***
			(0.00)	(0.00)	(0.01)	(0.11)	(0.00)				(0.00)	(0.00)	(0.02)	(0.49)	(0.00)
	12 M	0.04	− 0.0019***	0.3377***	0.0163***	0.0073***	0.0021***		12 M	0.58	0.3508***	− 10.5717***	− 0.0005***	0.1834***	− 0.0038***
			(0.05)	(0.11)	(0.01)	(0.10)	(0.72)				(0.00)	(0.00)	(0.97)	(0.00)	(0.89)

This table lists the results of the regressions for EUR/USD. The values in parentheses are the p-values of the coefficients. In the expression of significance, “***” means the coefficient is significant at $p<0.01$, “**” means the coefficient is significant at $p<0.05$, and “*” means the coefficient is significant at $p<0.10$

## Appendix 2: Dynamic process of the relative price

By assuming the jump amplitudes of the dynamic process of the exchange rate in Eq. (3) follow Normal distribution, ${Y}_{X,u}\sim Normal\left({\theta }_{X,u},{\nu }_{X,u}^{2}\right)$ and ${Y}_{X,d}\sim Normal\left({\theta }_{X,d},{\nu }_{X,d}^{2}\right)$, it has

85 $$\frac{dX\left(t\right)}{X\left(t-\right)}=\left\{{\mu }_{X}-{\lambda }_{u}\left[\mathrm{exp}\left({\theta }_{X,u}+\frac{{\nu }_{X,u}^{2}}{2}\right)-1\right]-{\lambda }_{d}\left[\mathrm{exp}\left({\theta }_{X,d}+\frac{{\nu }_{X,d}^{2}}{2}\right)-1\right]\right\}dt+{\sigma }_{X}d{W}_{X}^{\mathbb{P}}\left(t\right)+{Y}_{X,u}d{N}_{u}\left(t\right)+{Y}_{X,d}d{N}_{d}\left(t\right)$$

Let the relative price to be $\frac{X\left(t\right){P}_{F}\left(t,T\right)}{{P}_{D}\left(t,T\right)}$. By applying Itô’s lemma, the dynamic process of the relative price is as follows.

86 $$d\frac{X\left(t\right){P}_{F}\left(t,T\right)}{{P}_{D}\left(t,T\right)}=\frac{X\left(t\right){P}_{F}\left(t,T\right)}{{P}_{D}\left(t,T\right)}\left\{\frac{dX\left(t\right)}{X\left(t-\right)}+\frac{d{P}_{F}\left(t,T\right)}{{P}_{F}\left(t -,T\right)}-\frac{d{P}_{D}\left(t,T\right)}{{P}_{D}\left(t -,T\right)}+\frac{dX\left(t\right)}{X\left(t-\right)}\frac{d{P}_{F}\left(t,T\right)}{{P}_{F}\left(t -,T\right)}-\frac{dX\left(t\right)}{X\left(t-\right)}\frac{d{P}_{D}\left(t,T\right)}{{P}_{D}\left(t -,T\right)}-\frac{d{P}_{F}\left(t,T\right)}{{P}_{F}\left(t -,T\right)}\frac{d{P}_{D}\left(t,T\right)}{{P}_{D}\left(t -,T\right)}+{\left[\frac{d{P}_{D}\left(t,T\right)}{{P}_{D}\left(t -,T\right)}\right]}^{2}\right\}$$

87 $$=\frac{X\left(t\right){P}_{F}\left(t,T\right)}{{P}_{D}\left(t,T\right)}\left\{U\left(t,T\right)dt+\xi \left(t,T\right)d{\widetilde{W}}^{\mathbb{P}}\left(t\right)+{J}_{u}d{N}_{u}\left(t\right)+{J}_{d}\left(t\right)d{N}_{d}\left(t\right)+{J}_{u,d}\left(t\right)d{N}_{u,d}\left(t\right)\right\}$$

where

$$U\left(t,T\right)={\mu }_{X}-{r}_{D}\left(t\right)+{r}_{F}\left(t\right)+{\alpha }_{D}^{*}\left(t,T\right)-{\alpha }_{F}^{*}\left(t,T\right)-\frac{1}{2}{\sigma }_{X}^{2}+\frac{1}{2}{\xi }^{2}\left(t,T\right)-{\lambda }_{u}\left[\mathrm{exp}\left({\theta }_{X,u}+\frac{{\nu }_{X,u}^{2}}{2}\right)-1\right]-{\lambda }_{d}\left[\mathrm{exp}\left({\theta }_{X,d}+\frac{{\nu }_{X,d}^{2}}{2}\right)-1\right]{\xi }^{2}\left(t,T\right)={\sigma }_{X}^{2}+{{\sigma }_{D}^{*}}^{2}\left(t,T\right)+{{\sigma }_{F}^{*}}^{2}\left(t,T\right)-2{\rho }_{XF}{\sigma }_{X}{\sigma }_{F}^{*}\left(t,T\right)+2{\rho }_{XD}{\sigma }_{X}{\sigma }_{D}^{*}\left(t,T\right)-2{\rho }_{DF}{\sigma }_{F}^{*}\left(t,T\right){\sigma }_{D}^{*}\left(t,T\right)\xi \left(t,T\right)d{\widetilde{W}}^{\mathbb{P}}\left(t\right)=\left[\begin{array}{ccc}{\sigma }_{X}& -{\widehat{\sigma }}_{F}& {\widehat{\sigma }}_{D}\end{array}\right]\left[\begin{array}{c}d{W}_{X}^{\mathbb{P}}\left(t\right)\\ d{W}_{F}^{\mathbb{P}}\left(t\right)\\ d{W}_{D}^{\mathbb{P}}\left(t\right)\end{array}\right]={\sigma }_{X}d{W}_{X}^{\mathbb{P}}\left(t\right)-{\sigma }_{F}^{*}\left(t,T\right)d{W}_{F}^{\mathbb{P}}\left(t\right)+{\sigma }_{D}^{*}\left(t,T\right)d{W}_{D}^{\mathbb{P}}\left(t\right){J}_{u}={Y}_{X,u}-{Y}_{F,u}^{*}+{Y}_{D,u}^{*}-{Y}_{X,u}{Y}_{F,u}^{*}+{Y}_{X,u}{Y}_{D,u}^{*}-{Y}_{F,u}^{*}{Y}_{D,u}^{*}+{{Y}_{D,u}^{*}}^{2}{J}_{d}={Y}_{X,d}-{Y}_{F,d}^{*}+{Y}_{D,d}^{*}-{Y}_{X,d}{Y}_{F,d}^{*}+{Y}_{X,d}{Y}_{D,d}^{*}-{Y}_{F,d}^{*}{Y}_{D,d}^{*}+{{Y}_{D,d}^{*}}^{2}{J}_{u,d}=-{Y}_{X,d}{Y}_{F,u}^{*}-{Y}_{X,u}{Y}_{F,d}^{*}+{Y}_{X,u}{Y}_{D,d}^{*}+{Y}_{X,d}{Y}_{D,u}^{*}-{Y}_{F,u}^{*}{Y}_{D,d}^{*}-{Y}_{F,d}^{*}{Y}_{D,u}^{*}+2{Y}_{D,u}^{*}{Y}_{D,d}^{*}$$

In the dynamic model, this research considers a large number of partitions of the time interval to get the differential of $t$. The occurrence of the Poisson process is a rare event as the intensity $\lambda dt$ is small, so the probability of the event that an upside jump and a downside jump occur simultaneously will be negligibly small. The quadratic Poisson $d{N}_{u}\left(t\right)d{N}_{d}\left(t\right)$ is ignored, so that it leads to ${J}_{u,d}d{N}_{u,d}\left(t\right)=0$.

Let ${Y}_{T,u}$ and ${Y}_{T,d}$ be the total upside jump size and downside jump size. The aggregate jump size is a mixed distribution composed by the jump sizes in the foreign exchange rate model and interest rate models.

88 $${J}_{u}d{N}_{u}\left(t\right)=\left({Y}_{X,u}-{Y}_{F,u}^{*}+{Y}_{D,u}^{*}-{Y}_{X,u}{Y}_{F,u}^{*}+{Y}_{X,u}{Y}_{D,u}^{*}-{Y}_{F,u}^{*}{Y}_{D,u}^{*}+{{Y}_{D,u}^{*}}^{2}\right)d{N}_{u}\left(t\right)\approx {Y}_{T,u} d{N}_{u}\left(t\right)$$

89 $${J}_{d}d{N}_{d}\left(t\right)=\left({Y}_{X,d}-{Y}_{F,d}^{*}+{Y}_{D,d}^{*}-{Y}_{X,d}{Y}_{F,d}^{*}+{Y}_{X,d}{Y}_{D,d}^{*}-{Y}_{F,d}^{*}{Y}_{D,d}^{*}+{{Y}_{D,d}^{*}}^{2}\right)d{N}_{d}\left(t\right)\approx {Y}_{T,d} d{N}_{d}\left(t\right)$$

90 $$d\frac{X\left(t\right){P}_{F}\left(t,T\right)}{{P}_{D}\left(t,T\right)}=\frac{X\left(t\right){P}_{F}\left(t,T\right)}{{P}_{D}\left(t,T\right)}\left\{U\left(t,T\right)dt+\xi \left(t,T\right)d{\widetilde{W}}^{\mathbb{P}}\left(t\right)+{Y}_{T,u} d{N}_{u}\left(t\right)+{Y}_{T,d} d{N}_{d}\left(t\right)\right\}$$

91 $$\frac{X\left(t\right){P}_{F}\left(t,T\right)}{{P}_{D}\left(t,T\right)}=\frac{X\left(0\right){P}_{F}\left(0,T\right)}{{P}_{D}\left(0,T\right)}\cdot \mathrm{exp}\left\{{\int }_{0}^{t}\left[U\left({t}_{\alpha },T\right)-\frac{1}{2}{\xi }^{2}\left({t}_{\alpha },T\right)\right]d{t}_{\alpha }+{\int }_{0}^{t}\xi \left({t}_{\alpha },T\right)d{\widetilde{W}}^{\mathbb{P}}\left({t}_{\alpha }\right)\right\}\cdot \mathrm{exp}\left[{Y}_{T,u}d{N}_{u}\left(t\right)\right]\cdot \mathrm{exp}\left[{Y}_{T,d}d{N}_{d}\left(t\right)\right]$$

92 $$d\mathrm{ln}\frac{X\left(t\right){P}_{F}\left(t,T\right)}{{P}_{D}\left(t,T\right)}=\left[U\left(t,T\right)-\frac{1}{2}{\xi }^{2}\left(t,T\right)\right]dt+\xi \left(t,T\right)d{\widetilde{W}}^{\mathbb{P}}\left(t\right)+{Y}_{T,u} d{N}_{u}\left(t\right)+{Y}_{T,d} d{N}_{d}\left(t\right)$$

In this paper, the Normal distribution are employed as the target to approximate the aggregate distribution of the total jump sizes. That is, the distributions of ${Y}_{T,u}$ and ${Y}_{T,d}$ are assumed to be Normal distributions, ${Y}_{T,u}\sim Normal\left({\theta }_{T,u},{\nu }_{T,u}^{2}\right)$ and ${Y}_{T,d}\sim Normal\left({\theta }_{T,d},{\nu }_{T,d}^{2}\right)$.

Similar to the MGF of interest rates, the MGF of the compound Poisson process ${J}_{X,u}={Y}_{T,u} d{N}_{u}\left(t\right)$ with parameter ${h}_{X,u}$ is

93 $${M}_{{J}_{X,u}}\left({h}_{X,u}\right)=\mathrm{exp}\left\{{\lambda }_{u}dt\left[{M}_{{Y}_{T,u}}\left({h}_{X,u}\right)-1\right]\right\}$$

where ${M}_{{Y}_{T,u}}\left({h}_{X,u}\right)=\mathrm{exp}\left({h}_{X,u}{\theta }_{T,u}+\frac{1}{2}{h}_{X,u}^{2}{\nu }_{T,u}^{2}\right)$ is the MGF of the distribution $Normal ({\theta }_{T,u},{\nu }_{T,u}^{2})$.

The MGF of the compound Poisson process ${J}_{X,d}={Y}_{T,d} d{N}_{d}\left(t\right)$ with parameter ${h}_{X,d}$ is

94 $${M}_{{J}_{d}}\left({h}_{X,d}\right)=\mathrm{exp}\left\{{\lambda }_{d}dt\left[{M}_{{Y}_{T,d}}\left({h}_{X,d}\right)-1\right]\right\}$$

where ${M}_{{Y}_{T,d}}\left({h}_{X,d}\right)=\mathrm{exp}\left({h}_{X,d}{\theta }_{T,d}+\frac{1}{2}{h}_{X,d}^{2}{\nu }_{T,d}^{2}\right)$ is the MGF of the distribution $Normal \left({\theta }_{T,d},{\nu }_{T,d}^{2}\right)$.

The Radon-Nikodým derivative of the Esscher transform with respect to the dynamic process of relative price is given by:

95 $${\xi }^{{h}_{X}}\left(t\right)=\left.\frac{d{\mathbb{Q}}^{{h}_{X}}}{d{\mathbb{P}}}\right|\mathcal{F}\left(t\right)$$

96 $$=\frac{\mathrm{exp}\left\{{h}_{1,X}\cdot \xi \left(t,T\right)d{\widetilde{W}}^{\mathbb{P}}\left(t\right)\right\}}{{\mathbb{E}}^{\mathbb{P}}\left\{\mathrm{exp}\left[{h}_{1,X}\cdot \xi \left(t,T\right)d{\widetilde{W}}^{\mathbb{P}}\left(t\right)\right]\right\}}\cdot \frac{\mathrm{exp}\left\{{h}_{X,u}\cdot {Y}_{T,u} d{N}_{u}\left(t\right) \right\}}{{\mathbb{E}}^{\mathbb{P}}\left\{\mathrm{exp}\left[{h}_{X,u}\cdot {Y}_{T,u} d{N}_{u}\left(t\right)\right]\right\}}\cdot \frac{\mathrm{exp}\left\{{h}_{X,d}\cdot {Y}_{T,d} d{N}_{d}\left(t\right)\right\}}{{\mathbb{E}}^{\mathbb{P}}\left\{\mathrm{exp}\left[{h}_{X,d}\cdot {Y}_{T,d} d{N}_{d}\left(t\right)\right]\right\}}$$

97 $$=\mathrm{exp}\left[{h}_{1,X}\xi \left(t,T\right)d{\widetilde{W}}^{\mathbb{P}}\left(t\right)-\frac{1}{2}{h}_{1,X}^{2}{\xi }^{2}\left(t,T\right)dt\right]\cdot \mathrm{exp}\left\{{h}_{X,u}\cdot {Y}_{T,u} d{N}_{u}\left(t\right)-{\lambda }_{u}dt\left[\mathrm{exp}\left({h}_{X,u}{\theta }_{T,u}+\frac{1}{2}{h}_{X,u}^{2}{\nu }_{T,u}^{2}\right)-1\right]\right\}\cdot \mathrm{exp}\left\{{h}_{X,d}\cdot {Y}_{T,d} d{N}_{d}\left(t\right)-{\lambda }_{d}dt\left[\mathrm{exp}\left({h}_{X,d}{\theta }_{T,d}+\frac{1}{2}{h}_{X,d}^{2}{\nu }_{T,d}^{2}\right)-1\right]\right\}$$

The distributions of jump size and jump intensity under ${\mathbb{Q}}$ measure are still need to be found. Let ${\Lambda }_{t}^{{h}_{X}}=\left.\frac{d{\mathbb{Q}}^{{h}_{X}}}{d{\mathbb{P}}}\right|\mathcal{F}\left(t\right)={\Lambda }_{t}^{{C}_{X}}\times {\Lambda }_{t}^{{J}_{X}}$, for the jump part, it has

98 $${\Lambda }_{t}^{{J}_{X}}=\left.\frac{d{\mathbb{Q}}^{{J}_{X}}}{d{\mathbb{P}}^{{J}_{X}}}\right|\mathcal{F}\left(t\right)=\frac{\mathrm{exp}\left\{{h}_{X,u}\cdot {Y}_{T,u} d{N}_{u}\left(t\right) \right\}}{{\mathbb{E}}^{\mathbb{P}}\left\{\mathrm{exp}\left[{h}_{X,u}\cdot {Y}_{T,u} d{N}_{u}\left(t\right)\right]\right\}}\cdot \frac{\mathrm{exp}\left\{{h}_{X,d}\cdot {Y}_{T,d} d{N}_{d}\left(t\right)\right\}}{{\mathbb{E}}^{\mathbb{P}}\left\{\mathrm{exp}\left[{h}_{X,d}\cdot {Y}_{T,d} d{N}_{d}\left(t\right)\right]\right\}}$$

99 $$d{\mathbb{Q}}^{{J}_{X}}={\sum }_{{n}_{u}=1}^{\infty }\frac{\mathrm{exp}\left[-{\lambda }_{u}dt{M}_{{Y}_{T,u}}\left({h}_{X,u}\right)\right]\cdot {\left[{\lambda }_{u}dt\cdot {M}_{{Y}_{T,u}}\left({h}_{X,u}\right)\right]}^{{n}_{u}}}{{n}_{u}!}\cdot {\prod }_{n=1}^{{n}_{u}}\frac{\mathrm{exp}\left({h}_{X,u}\cdot {Y}_{T,u,n}\right)\cdot {f}_{{Y}_{T,u}}^{\mathbb{P}}\left(x\right)}{{M}_{{Y}_{T,u}}\left({h}_{X,u}\right)} \cdot {\sum }_{{n}_{d}=1}^{\infty }\frac{\mathrm{exp}\left[-{\lambda }_{d}dt{M}_{{Y}_{T,d}}\left({h}_{X,d}\right)\right]\cdot {\left[{\lambda }_{d}dt\cdot {M}_{{Y}_{T,d}}\left({h}_{X,d}\right)\right]}^{{n}_{d}}}{{n}_{d}!}\cdot {\prod }_{n=1}^{{n}_{d}}\frac{\mathrm{exp}\left({h}_{X,d}\cdot {Y}_{T,d,n}\right)\cdot {f}_{{Y}_{T,d}}^{\mathbb{P}}\left(x\right)}{{M}_{{Y}_{T,d}}\left({h}_{X,d}\right)}$$

where $${f}_{{Y}_{T,u}}^{\mathbb{P}}\left(x\right)=Normal\left(x;{\theta }_{T,u},{\nu }_{T,u}^{2}\right)$$ and $${f}_{{Y}_{T,d}}^{\mathbb{P}}\left(x\right)=Normal\left(x;{\theta }_{T,d},{\nu }_{T,d}^{2}\right)$$.

Therefore, under the ${\mathbb{Q}}$ measure, the jump size of upside jump and downside jump are as follows:

$${f}_{{Y}_{T,u}}^{\mathbb{Q}}\left(x\right)=\frac{\mathrm{exp}\left({h}_{X,u}\cdot {Y}_{T,u,n}\right)\cdot {f}_{{Y}_{T,u}}^{\mathbb{P}}\left(x\right)}{{M}_{{Y}_{T,u}}\left({h}_{X,u}\right)}=\frac{1}{\sqrt{2\pi {v}_{T,u}^{2}}}\mathrm{exp}\left[-\frac{{\left(x-{\theta }_{T,u}-{h}_{X,u}{\nu }_{T,u}^{2}\right)}^{2}}{2{\nu }_{T,u}^{2}}\right]=Normal\left(x;{\theta }_{T,u}+{h}_{X,u}{\nu }_{T,u}^{2}, {\nu }_{T,u}^{2}\right){f}_{{Y}_{T,d}}^{\mathbb{Q}}\left(x\right)=\frac{\mathrm{exp}\left({h}_{X,d}\cdot {Y}_{T,d,n}\right)\cdot {f}_{{Y}_{T,d}}^{\mathbb{P}}\left(x\right)}{{M}_{{Y}_{T,d}}\left({h}_{X,d}\right)}=\frac{1}{\sqrt{2\pi {v}_{T,d}^{2}}}\mathrm{exp}\left[-\frac{{\left(x-{\theta }_{T,d}-{h}_{X,d}{\nu }_{T,d}^{2}\right)}^{2}}{2{\nu }_{T,d}^{2}}\right]=Normal\left(x;{\theta }_{T,d}+{h}_{X,d}{\nu }_{T,d}^{2},{\nu }_{T,d}^{2}\right)$$

Under the ${\mathbb{Q}}$ measure, the jump intensity of upside jump and downside jump are

$$d{N}_{u}\left(t\right)\sim Poisson\left({\lambda }_{u}dt{M}_{{Y}_{T,u}}\left({h}_{X,u}\right)\right)=Poisson\left({\lambda }_{u}dt\mathrm{exp}\left({h}_{X,u}{\theta }_{T,u}+\frac{1}{2}{h}_{X,u}^{2}{\nu }_{T,u}^{2}\right)\right)d{N}_{d}\left(t\right)\sim Poisson\left({\lambda }_{d}dt{M}_{{Y}_{T,d}}\left({h}_{X,d}\right)\right)=Poisson\left({\lambda }_{d}dt\mathrm{exp}\left({h}_{X,d}{\theta }_{T,d}+\frac{1}{2}{h}_{X,d}^{2}{\nu }_{T,d}^{2}\right)\right)$$

Since the domestic money market account is ${B}_{D}\left(t\right)=\mathrm{exp}\left[{\int }_{0}^{t}{r}_{D}\left({t}_{\alpha }\right)d{t}_{\alpha }\right]$, the martingale condition is

100 $${\mathbb{E}}^{{\mathbb{Q}}_{D}}\left\{\left.\frac{\frac{X\left(t\right){P}_{F}\left(t,T\right)}{{P}_{D}\left(t,T\right)}}{{B}_{D}\left(t\right)}\right|{\mathcal{F}}_{0}\right\}=\frac{\frac{X\left(0\right){P}_{F}\left(0,T\right)}{{P}_{D}\left(0,T\right)}}{{B}_{D}\left(0\right)}$$

101 $$\iff \left[U\left(t,T\right)-{r}_{D}\right]-\frac{1}{2}{h}_{1,X}^{2}{\xi }^{2}\left(t,T\right)-{\lambda }_{u}\left[\mathrm{exp}\left({h}_{X,u}{\theta }_{T,u}+\frac{1}{2}{h}_{X,u}^{2}{\nu }_{T,u}^{2}\right)-1\right]-{\lambda }_{d}\left[\mathrm{exp}\left({h}_{X,d}{\theta }_{T,d}+\frac{1}{2}{h}_{X,d}^{2}{\nu }_{T,d}^{2}\right)-1\right]=0$$

## Appendix 3: Pricing model

Consider a standard foreign exchange European call option with the exercise price K and the maturity date T. The payoff of this European call option at the maturity date T is as follows.

102 $$C\left(T\right)={\mathit{max}\left[X\left(T\right)-K\right]}^{+}.$$

Because ${P}_{D}\left(T,T\right)=1$ and ${P}_{F}\left(T,T\right)=1$, Eq. (102) can be rewritten as

103 $$C\left(T\right)=\left[\frac{X\left(T\right){P}_{F}\left(T,T\right)}{{P}_{D}\left(T,T\right)}-K\frac{{P}_{F}\left(T,T\right)}{{P}_{D}\left(T,T\right)}\right]\cdot {1}_{\left\{\frac{X\left(T\right){P}_{F}\left(T,T\right)}{{P}_{D}\left(T,T\right)}>K\right\}}.$$

Let ${n}_{u}$ and ${n}_{d}$ to be the number of upside jumps and downside jumps occurred during the time interval $\left[t,T\right]$. Applying the law of iterated expectation, under ${\mathbb{Q}}_{D}^{T}$ measure, the present value of the call option at time $t$ is

104 $$C\left(t\right)={P}_{D}\left(t,T\right){\mathbb{E}}^{{\mathbb{Q}}_{D}^{T}}\left\{{\mathbb{E}}^{{\mathbb{Q}}_{D}^{T}}\left[\left.C\left(T\right)\right|\mathcal{F}\left(t\right),{n}_{u},{n}_{d}\right]\right\}$$

105 $$={P}_{D}\left(t,T\right){\mathbb{E}}^{{\mathbb{Q}}_{D}^{T}}\left\{{\mathbb{E}}^{{\mathbb{Q}}_{D}^{T}}\left\{\left.\left[\frac{X\left(T\right){P}_{F}\left(T,T\right)}{{P}_{D}\left(T,T\right)}-K\right]\cdot {1}_{\left\{\frac{X\left(T\right){P}_{F}\left(T,T\right)}{{P}_{D}\left(T,T\right)}>K\right\}}\right|\mathcal{F}\left(t\right),{n}_{u},{n}_{d}\right\}\right\}$$

106 $$={P}_{D}\left(t,T\right){\mathbb{E}}^{{\mathbb{Q}}_{D}^{T}}\left\{{\mathbb{E}}^{{\mathbb{Q}}_{D}^{T}}\left\{\left.\frac{X\left(T\right){P}_{F}\left(T,T\right)}{{P}_{D}\left(T,T\right)}\cdot {1}_{\left\{\frac{X\left(T\right){P}_{F}\left(T,T\right)}{{P}_{D}\left(T,T\right)}>K\right\}}\right|\mathcal{F}\left(t\right),{n}_{u},{n}_{d}\right\}\right\}-K{P}_{D}\left(t,T\right){\mathbb{E}}^{{\mathbb{Q}}_{D}^{T}}\left\{{\mathbb{E}}^{{\mathbb{Q}}_{D}^{T}}\left\{\left.{1}_{\left\{\frac{X\left(T\right){P}_{F}\left(T,T\right)}{{P}_{D}\left(T,T\right)}>K\right\}}\right|\mathcal{F}\left(t\right),{n}_{u},{n}_{d}\right\}\right\}.$$

Let ${E}_{1}={\mathbb{E}}^{{\mathbb{Q}}_{D}^{T}}\left\{\left.\frac{X\left(T\right){P}_{F}\left(T,T\right)}{{P}_{D}\left(T,T\right)}\cdot {1}_{\left\{\frac{X\left(T\right){P}_{F}\left(T,T\right)}{{P}_{D}\left(T,T\right)}>K\right\}}\right|\mathcal{F}\left(t\right),{n}_{u},{n}_{d}\right\}$ and ${E}_{2}={\mathbb{E}}^{{\mathbb{Q}}_{D}^{T}}\left\{\left.{1}_{\left\{\frac{X\left(T\right){P}_{F}\left(T,T\right)}{{P}_{D}\left(T,T\right)}>K\right\}}\right|\mathcal{F}\left(t\right),{n}_{u},{n}_{d}\right\}$. The ${E}_{1}$ can be rewritten as

107 $${E}_{1}={\mathbb{E}}^{{\mathbb{Q}}_{D}^{T}}\left\{\left.\frac{X\left(t\right){P}_{F}\left(t,T\right)}{{P}_{D}\left(t,T\right)}\mathrm{exp}\left\{{\int }_{t}^{T}\left[U\left({t}_{\alpha },T\right)-\frac{1}{2}{\xi }^{2}\left({t}_{\alpha },T\right)\right]d{t}_{\alpha }+{\int }_{t}^{T}\xi \left({t}_{\alpha },T\right)d{\widetilde{W}}^{\mathbb{P}}\left({t}_{\alpha }\right)+{n}_{u}\cdot {Y}_{T,u}+{n}_{d}\cdot {Y}_{T,d}\right\}\cdot {1}_{\left\{\frac{X\left(T\right){P}_{F}\left(T,T\right)}{{P}_{D}\left(T,T\right)}>K\right\}}\right|\mathcal{F}\left(t\right),{n}_{u},{n}_{d}\right\},$$

where

$$\Theta \left({n}_{u},{n}_{d},{h}_{X,\mathrm{u}}\right)={n}_{u}\left({\theta }_{T,\mathrm{u}}+{h}_{X,u}{\nu }_{T,u}^{2}\right)+{n}_{d}\left({\theta }_{T,\mathrm{d}}+{h}_{X,\mathrm{d}}{\nu }_{T,d}^{2}\right),$$

and

$$\Phi \left({n}_{u},{n}_{d},t,T\right)={\xi }^{2}\left(t,T\right)+{n}_{u}^{2}{\nu }_{T,u}^{2}+{n}_{d}^{2}{\nu }_{T,d}^{2}.$$

Define a new random variable ${\varepsilon }^{{\mathbb{Q}}_{D}^{T}}$ follows the standard distribution, which is

$${\varepsilon }^{{\mathbb{Q}}_{D}^{T}}=\left.\frac{\left[{\int }_{t}^{T}\xi \left({t}_{\alpha },T\right)d{\widetilde{{\varvec{W}}}}^{{\mathbb{Q}}_{D}^{T}}\left({t}_{\alpha }\right)+{n}_{u}\cdot {Y}_{T,u,n}+{n}_{d}\cdot {Y}_{T,d,n}\right]-\Theta \left({n}_{u},{n}_{d},{h}_{X,u}\right)}{\sqrt{\Phi \left({n}_{u},{n}_{d},t,T\right)}}\right| {n}_{u},{n}_{d}\sim Normal\left(\mathrm{0,1}\right)$$

Therefore, the dynamic process of relative price under ${\mathbb{Q}}_{D}^{T}$ measure can be rewritten as

108 $$\frac{\mathrm{X}\left(\mathrm{T}\right){\mathrm{P}}_{\mathrm{F}}\left(T,T\right)}{{P}_{D}\left(T,T\right)}\stackrel{d}{=}\frac{X\left(t\right){P}_{F}\left(t,T\right)}{{P}_{D}\left(t,T\right)}\cdot \mathrm{exp}\left[\Gamma \left({n}_{u},{n}_{d},t,T\right)-\frac{1}{2}\Phi \left({n}_{u},{n}_{d},t,T\right)+\sqrt{\Phi \left({n}_{u},{n}_{d},t,T\right)}{\varepsilon }^{{\mathbb{Q}}_{D}^{T}}\right]$$

where

$$\Gamma \left({n}_{u},{n}_{d},t,T\right)=-{\lambda }_{u}\left[\mathrm{exp}\left({h}_{X,u}{\theta }_{T,u}+\frac{1}{2}{h}_{X,u}^{2}{\nu }_{T,u}^{2}\right)-1\right]\left(T-t\right)-{\lambda }_{d}\left[\mathrm{exp}\left({h}_{X,d}{\theta }_{T,d}+\frac{1}{2}{h}_{X,d}^{2}{\nu }_{T,d}^{2}\right)-1\right]\left(T-t\right)+{n}_{u}\left[{\theta }_{T,u}+{h}_{X,u}{\nu }_{T,u}^{2}+\frac{1}{2}{\nu }_{T,u}^{2}\right]+{n}_{d}\left[{\theta }_{T,d}+{h}_{X,d}{\nu }_{T,d}^{2}+\frac{1}{2}{\nu }_{T,d}^{2}\right].$$

Therefore, ${E}_{1}$ can be rewritten as

109 $${E}_{1}=\frac{X\left(t\right){P}_{F}\left(t,T\right)}{{P}_{D}\left(t,T\right)}\mathrm{exp}\left[\Gamma \left({n}_{u},{n}_{d},t,T\right)\right]\cdot {\mathbb{E}}^{{\mathbb{Q}}_{D}^{T}}\left\{\left.\mathrm{exp}\left[-\frac{1}{2}\Phi \left({n}_{u},{n}_{d},t,T\right)+\sqrt{\Phi \left({n}_{u},{n}_{d},t,T\right)}{\varepsilon }^{{\mathbb{Q}}_{D}^{T}}\right]\cdot {1}_{\left\{\frac{X\left(T\right){P}_{F}\left(T,T\right)}{{P}_{D}\left(T,T\right)}>K\right\}}\right|{n}_{u},{n}_{d}\right\}$$

110 $$=\frac{X\left(t\right){P}_{F}\left(t,T\right)}{{P}_{D}\left(t,T\right)}\mathrm{exp}\left[\Gamma \left({n}_{u},{n}_{d},t,T\right)\right]\cdot {\mathbb{E}}^{{\mathbb{R}}_{D}^{T}}\left\{\left.{1}_{\left\{\frac{X\left(T\right){P}_{F}\left(T,T\right)}{{P}_{D}\left(T,T\right)}>K\right\}}\right|{n}_{u},{n}_{d}\right\}$$

111 $$=\frac{X\left(t\right){P}_{F}\left(t,T\right)}{{P}_{D}\left(t,T\right)}\mathrm{exp}\left[\Gamma \left({n}_{u},{n}_{d},t,T\right)\right]N\left({d}_{1}\right)$$

where

$${d}_{1}=\frac{\mathrm{ln}\frac{X\left(t\right){P}_{F}\left(t,T\right)}{{P}_{D}\left(t,T\right)}+\Gamma \left({n}_{u},{n}_{d},t,T\right)+\frac{1}{2}\Phi \left({n}_{u},{n}_{d},t,T\right)}{\sqrt{\Phi \left({n}_{u},{n}_{d},t,T\right)}},$$

and $N\left(\cdot \right)$ is cumulative distribution function of standard normal distribution.

Similarly, ${E}_{2}$ can be rewritten as

112 $${E}_{2}={\mathbb{E}}^{{\mathbb{R}}_{D}^{T}}\left\{\left.{1}_{\left\{\frac{X\left(T\right){P}_{F}\left(T,T\right)}{{P}_{D}\left(T,T\right)}>K\right\}}\right|\mathcal{F}\left(t\right),{n}_{u},{n}_{d}\right\}=N\left({d}_{2}\right)$$

where

$${d}_{2}={d}_{1}-\sqrt{\Phi \left({n}_{u},{n}_{d},t,T\right)}.$$

Subtitling Eq. (111) and (112) into Eq. (106), the pricing formula of European currency call option with correlated jumps can be obtained.

113 $$C\left(t\right)=X\left(t\right){P}_{F}\left(t,T\right){\sum }_{{n}_{u}=0}^{\infty }{\sum }_{{n}_{d}=0}^{\infty }\frac{\mathrm{exp}\left[-{q}_{u}\left(t,T\right)\right]{\left[{q}_{u}\left(t,T\right)\right]}^{{n}_{u}}}{{n}_{u}!}\cdot \frac{\mathrm{exp}\left[-{q}_{d}\left(t,T\right)\right]{\left[{q}_{d}\left(t,T\right)\right]}^{{n}_{d}}}{{n}_{d}!}\mathrm{exp}\left[\Gamma \left({n}_{u},{n}_{d},t,T\right)\right] N\left({d}_{1}\right)-K{P}_{D}\left(t,T\right){\sum }_{{n}_{u}=0}^{\infty }{\sum }_{{n}_{d}=0}^{\infty }\frac{\mathrm{exp}\left[-{q}_{u}\left(t,T\right)\right]{\left[{q}_{u}\left(t,T\right)\right]}^{{n}_{u}}}{{n}_{u}!}\cdot \frac{\mathrm{exp}\left[-{q}_{d}\left(t,T\right)\right]{\left[{q}_{d}\left(t,T\right)\right]}^{{n}_{d}}}{{n}_{d}!}\mathrm{exp}\left[\Gamma \left({n}_{u},{n}_{d},t,T\right)\right]N\left({d}_{2}\right)$$

where

$${q}_{u}\left(t,T\right)={\lambda }_{u}\left(T-t\right)\mathrm{exp}\left({h}_{X,u}{\theta }_{T,u}+\frac{1}{2}{h}_{X,u}^{2}{\nu }_{T,u}^{2}\right),{q}_{d}\left(t,T\right)={\lambda }_{d}\left(T-t\right)\mathrm{exp}\left({h}_{X,d}{\theta }_{T,d}+\frac{1}{2}{h}_{X,d}^{2}{\nu }_{T,d}^{2}\right),$$

${q}_{u}\left(t,T\right)$ and ${q}_{d}\left(t,T\right)$ are the upside and downside jump intensity under ${\mathbb{Q}}_{D}^{T}$ measure, and $\Theta \left({n}_{u},{n}_{d},{h}_{X,u}\right)$ and $\Phi \left({n}_{u},{n}_{d},t,T\right)$ are the mean and variance of the relative price $\frac{X\left(t\right){P}_{F}\left(t,T\right)}{{P}_{D}\left(t,T\right)}$ under ${\mathbb{Q}}_{D}^{T}$ measure.

## Abbreviations

ATMV: Delta-neutral straddle
BF10: 10-Delta Butterfly Spreads
BF25: 25-Delta Butterfly Spreads
CB: Correlated Brownian
CB–CAJ: Correlated Brownian–Correlated Asymmetric Jump
CB–CSJ: Correlated Brownian–Correlated Symmetric Jump
CB–ISJ: Correlated Brownian–Independent Symmetric Jump
CDF: Cumulative Distribution Function
CHF: Swiss francs
DAX: Deutscher Aktienindex
DEM: German marks
DJI: Dow Jones Industrial Average Index
ECB: European Central Bank
EM: Expectation–Maximization
EU: European Union
EUR: Euros
Fed: Federal Reserve
FTSE 100: Financial Times Stock Exchange 100 Index
FX: Foreign Exchange
GBP: Great British pounds
HJM: Heath–Jarrow–Morton Model
IRP: Interest Rate Parity
IV: Implied Volatility
JPY: Japanese yen
LIBOR: London Interbank Offered Rate
LRT: Likelihood Ratio Test
MGF: Moment Generating Function
MLE: Maximum Likelihood Estimation
MSE: Means of Squares Pricing Error
NASDAQ 100: NASDAQ Composite 100 Index
OilGas: NYSE ARCA Oil & Gas Index
OIS: Overnight Indexed Swap
QE: Quantitative Easing
RMSE: Root Mean Square Error
RR10: 10-Delta Risk Reversals
RR25: 25-Delta Risk Reversals
S&P 500: Standard & Poor's 500 index
U.S.: United States
USD: United States dollars
ZCB: Zero-Coupon Bond

## Footnotes

1 In the statistics of the 1-month US LIBOR, 1-month EU LIBOR, and spot EUR/USD exchange rates from October 1, 2003, to December 31, 2020 (4502 observed days), on a total of 143 days, at least one of the three assets’ return was outside three standard deviations. On 5 days (0.11%) out of 4502 days, the returns of three assets were simultaneously outside three standard deviations.

2 According to Michaelides (2021), the appropriate significance level for a sample size of 4502 is calculated as $\mathrm{min}\left\{0.5,\frac{0.01}{\sqrt{4502/100}}\right\}=0.00149$.

## References

[CR1] Alexeev V, Urga G, Yao W (2019). Asymmetric jump beta estimation with implications for portfolio risk management. Int Rev Econ Financ.

[CR2] Aliber RZ (1973). The interest rate parity theorem: a reinterpretation. J Polit Econ.

[CR3] Amin KI, Jarrow RA (1991). Pricing foreign currency options under stochastic interest rates. J Int Money Finance.

[CR4] Bakshi G, Carr P, Wu L (2008). Stochastic risk premiums, stochastic skewness in currency options, and stochastic discount factors in international economies. J Financ Econ.

[CR5] Balke NS, Wohar ME (1998). Nonlinear dynamics and covered interest rate parity. Empir Econ.

[CR6] Bates DS (1992) Jumps and stochastic processes implicit in PHLX foreign currency options. Working paper, Wharton School, University of Pennsylvania

[CR7] Bates DS (1996). Dollar jump fears, 1984–1992: distributional abnormalities implicit in currency futures options. J Int Money Finance.

[CR8] Bates DS (1996). Jumps and stochastic volatility: exchange rate processes implicit in Deutsche mark options. Rev Financ Stud.

[CR9] Bo L, Wang Y, Yang X (2010) Markov-modulated jump–diffusions for currency option pricing. Insurance Math Econ 46(3):461–469

[CR10] Brigo D, Mercurio F (2006) Interest rate models-theory and practice: with smile, inflation and credit, vol 2. Springer, Berlin

[CR11] Britten-Jones M, Neuberger A (2000). Option prices, implied price processes, and stochastic volatility. J Finance.

[CR12] Byun SJ, Jeon BH, Min B, Yoon S-J (2015). The role of the variance premium in Jump-GARCH option pricing models. J Bank Finance.

[CR13] Carr P, Wu L (2007). Stochastic skew in currency options. J Financ Econ.

[CR14] Chan WH, Feng L (2012). Time-varying jump risk premia in stock index futures returns. J Futures Mark.

[CR15] Chang HL, Chang YC, Cheng HW, Peng PH, Tseng K (2019). Jump variance risk: evidence from option valuation and stock returns. J Futures Mark.

[CR16] Cheng H-W, Lo C-L, Tsai JT (2020). Model specification of conditional jump intensity: evidence from S&P 500 returns and option prices. N Am J Econ Finance.

[CR17] Chesney M, Scott L (1989). Pricing European currency options: a comparison of the modified Black-Scholes model and a random variance model. J Financ Quant Anal.

[CR18] Chinn MD (2006). The (partial) rehabilitation of interest rate parity in the floating rate era: longer horizons, alternative expectations, and emerging markets. J Int Money Finance.

[CR19] Christoffersen P, Jacobs K, Ornthanalai C (2012). Dynamic jump intensities and risk premiums: evidence from S&P 500 returns and options. J Financ Econ.

[CR20] Chuang M-C, Wen C-H, Lin S-K (2020). Valuation and empirical analysis of currency options. Int Rev Econ Finance.

[CR21] Cookson R (1992). Models of imperfection. Risk.

[CR22] Cosandier P-A, Lang BR (1981). Interest rate parity tests: Switzerland and some major western countries. J Bank Finance.

[CR23] Dahlquist M, Pénasse J (2022). The missing risk premium in exchange rates. J Financ Econ.

[CR24] De Santis G, Gerard B (1998). How big is the premium for currency risk?. J Financ Econ.

[CR25] Doffou A, Hilliard JE (2001). Pricing currency options under stochastic interest rates and jump-diffusion processes. J Financ Res.

[CR26] Doukas J, Hall PH, Lang LH (1999). The pricing of currency risk in Japan. J Bank Finance.

[CR27] Du W, Tepper A, Verdelhan A (2018). Deviations from covered interest rate parity. J Finance.

[CR28] Ekvall N, Jennergren LP, Näslund B (1997). Currency option pricing with mean reversion and uncovered interest parity: a revision of the Garman-Kohlhagen model. Eur J Oper Res.

[CR29] Esscher F (1932). On the probability function in the collective theory of risk. Skandinavisk Aktuarietidskrift.

[CR30] Frame SJ, Ramezani CA (2014). Bayesian estimation of asymmetric jump-diffusion processes. Ann Financ Econ.

[CR31] Garman MB, Kohlhagen SW (1983). Foreign currency option values. J Int Money Finance.

[CR32] Gerber HU, Shiu ESW (1994). Option pricing by Esscher transforms. Trans Soc Actuaries.

[CR33] Gerber HU, Shiu ESW (1996) Actuarial bridges to dynamic hedging and option pricing. Insurance Math Econ 18(3):183–218

[CR34] Harrison JM, Pliska SR (1981). Martingales and stochastic integrals in the theory of continuous trading. Stoch Process Appl.

[CR35] Heath D, Jarrow R, Morton A (1992). Bond pricing and the term structure of interest rates: a new methodology for contingent claims valuation. Econom.

[CR36] Heston SL (1993). A closed-form solution for options with stochastic volatility with applications to bond and currency options. Rev Financ Stud.

[CR37] Jiang GJ, Tian YS (2005). The model-free implied volatility and its information content. Rev Financ Stud.

[CR38] Jurek JW, & Xu Z (2014) Option-implied currency risk premia. Available at SSRN 2338585

[CR39] Kim K-H, Yun S, Kim N-U, Ri J-H (2019). Pricing formula for European currency option and exchange option in a generalized jump mixed fractional Brownian motion with time-varying coefficients. Physica A.

[CR40] Kou G, Peng Y, Wang G (2014). Evaluation of clustering algorithms for financial risk analysis using MCDM methods. Inf Sci.

[CR41] Kou G, Xu Y, Peng Y, Shen F, Chen Y, Chang K, Kou S (2021). Bankruptcy prediction for SMEs using transactional data and two-stage multiobjective feature selection. Decis Support Syst.

[CR42] Lau KJ, Goh YK, Lai AC (2019). An empirical study on asymmetric jump diffusion for option and annuity pricing. PLoS ONE.

[CR43] Li J, Zinna G (2018). The variance risk premium: components, term structures, and stock return predictability. J Bus Econ Stat.

[CR44] Li T, Kou G, Peng Y, Philip SY (2021). An integrated cluster detection, optimization, and interpretation approach for financial data. IEEE Trans Cybern.

[CR45] Lian Y-M, Chen J-H, Liao S-L (2021). Cojump risks and their impacts on option pricing. Q Rev Econ Finance.

[CR46] Lin S-K, Lian Y-M, Liao S-L (2014). Pricing gold options under Markov-modulated jump-diffusion processes. Appl Financ Econo.

[CR47] Low BS, Zhang S (2005). The volatility risk premium embedded in currency options. J Financ Quant Anal.

[CR48] Merton RC (1973). Theory of rational option pricing. Bell J Econ Manag Sci.

[CR49] Merton RC (1976). Option pricing when underlying stock returns are discontinuous. J Financ Econ.

[CR50] Michaelides M (2021). Large sample size bias in empirical finance. Financ Res Lett.

[CR51] Musiela M, Rutkowski M (1998). Martingale methods in financial modelling.

[CR52] Ornthanalai C (2014). Levy jump risk: evidence from options and returns. J Financ Econ.

[CR53] Pan J (2002). The jump-risk premia implicit in options: evidence from an integrated time-series study. J Financ Econ.

[CR54] Park Y-H (2016). The effects of asymmetric volatility and jumps on the pricing of VIX derivatives. J Econom.

[CR55] Sarwar G, Krehbiel T (2000). Empirical performance of alternative pricing models of currency options. J Futures Markets Futures Opt Other Deriv Prod.

[CR56] Shokrollahi F, Kılıçman A (2015). Actuarial approach in a mixed fractional Brownian motion with jumps environment for pricing currency option. Adv Differ Equ.

[CR57] Tai C-S (2003). Can currency risk be a source of risk premium in explaining forward premium puzzle? Evidence from Asia-Pacific forward exchange markets. J Int Financ Markets Inst Money.

[CR58] Wu L, Carr P (2009). Variance risk premiums. Rev Financ Stud.

